# Site-selective and metal-free C–H nitration of biologically relevant *N*-heterocycles

**DOI:** 10.1007/s12272-021-01351-5

**Published:** 2021-10-18

**Authors:** Junghyea Moon, Hyun Ku Ji, Nayoung Ko, Harin Oh, Min Seo Park, Suho Kim, Prithwish Ghosh, Neeraj Kumar Mishra, In Su Kim

**Affiliations:** School of Pharmacy, Sungkyunkwan University, Suwon, 16419 Republic of Korea

**Keywords:** *t*-Butyl nitrite, C–H functionalization, Regioselectivity, Heterocycles, Nitration

## Abstract

The site-selective and metal-free C–H nitration reaction of quinoxalinones and pyrazinones as biologically important *N*-heterocycles with *t*-butyl nitrite is described. A wide range of quinoxalinones were efficiently applied in this transformation, providing C7-nitrated quinoxalinones without undergoing C3-nitration. From the view of mechanistic point, the radical addition reaction exclusively occurred at the electron-rich aromatic region beyond electron-deficient *N*-heterocycle ring. This is a first report on the C7–H functionalization of quinoxalinones under metal-free conditions. In contrast, the nitration reaction readily takes place at the C3-position of pyrazinones. This transformation is characterized by the scale-up compatibility, mild reaction conditions, and excellent functional group tolerance. The applicability of the developed method is showcased by the selective reduction of NO_2_ functionality on the C7-nitrated quinoxalinone product, providing aniline derivatives. Combined mechanistic investigations aided the elucidation of a plausible reaction mechanism.

**Supplementary Information:**

The online version contains supplementary material available at 10.1007/s12272-021-01351-5.

## Introduction

**Fig. 1:**
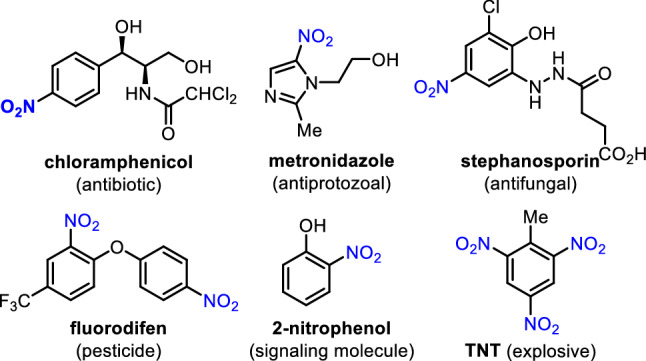
Nitro-containing pharmaceuticals and functional materials

Since the landmark discovery by Mitscherlich and Laurent in 1834 (Patel et al. 2021), nitro compounds are important and versatile building blocks in organic chemistry (Ono 2001), and their derivatives are widely utilized in various pharmaceuticals, agrochemicals, pigments, and dyes as well as a variety of fine chemicals such as solvents, perfumes, explosives, and polymers, as shown in Fig. 1 (Fan et al. 2004; McNamara et al. 2011; Nepali et al. 2019). For example, chloramphenicol (Rebstock et al. 1949) and metronidazole (Freeman et al. 1997) are well-known antibiotic and antiprotozoal drugs for the treatment of a number of infectious diseases. The biological properties of these molecules are closely related with (hetero)aryl motifs tethered with nitro functionality, but vary depending on the nature and position of substituents on (hetero)aryl rings.

Traditional methods for nitration reactions rely on an excess use of nitric acid or its mixture with sulfuric acid or dinitrogen pentoxide (Olah et al. 1990). However, from a synthetic point of view, these protocols represent the limited functional group tolerance and the generation of undesirable by-products as well as incomplete regioselectivity. To overcome these limitations, new nitrating agents such as nitrate (Manna et al. 2012; Zolfigol et al. 2012), nitrite salts (Fors et al. 2009; Li et al. 2013), and *tert*-butyl nitrite (TBN) have been intensively investigated (Wu and Neumann et al. 2011; Wu and Schranck et al. 2011; Shen et al. 2014).

**Fig. 2:**
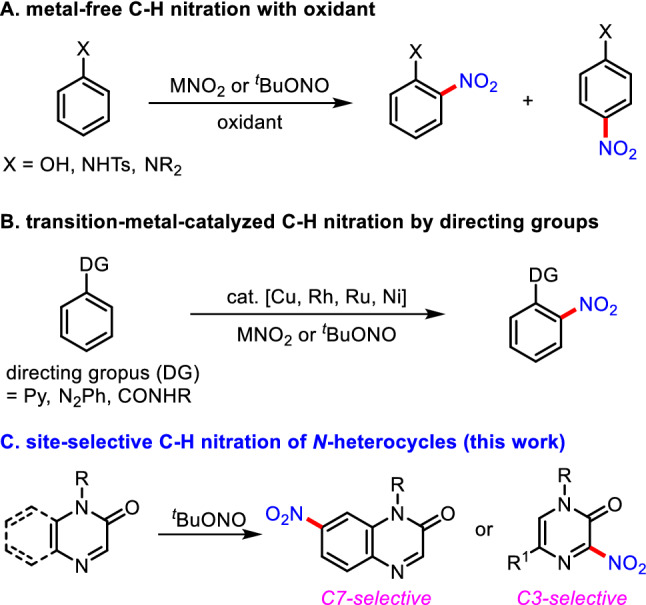
C–H nitration methods of (hetero)arenes using nitrating agents

With great advance on C–H functionalization reactions (Mishra et al. 2017, 2018; Pandey et al. 2018; Sambiagio et al. 2018; Lee et al. 2019), direct C–H nitration of (hetero)arenes has been recently developed. The metal-free oxidative C–H nitration of phenols or amines has been explored, as shown in Fig. 2 (Koley et al. 2009; Kilpatrick et al. 2013; Li et al. 2014). However, none of these protocols represent a general strategy to allow for complete site-selectivity between the *ortho*- and *para*-positions. A great deal of effort on site-selectivity of nitration has been devoted to the transition-metal-catalyzed *ortho*-C–H nitration of *N*-heterocycles. For example, Liu reported the Pd(II)-catalyzed *ortho*-C–H nitration of nitrogen-containing heterocycles with silver nitrite in the presence of K_2_S_2_O_8_ as an external oxidant (Liu et al. 2010). The *ortho*-C–H nitration of (hetero)arenes using nitrite salts was also realized with the Cu(II), Rh(III), Ru(0), and Ni(II) catalytic systems (Zhang et al. 2011; Xie et al. 2013; Katayev et al. 2014; Majhi et al. 2014; Fan and Ni 2016; Wan et al. 2017). In addition, the Pd(II)-catalyzed aerobic oxidative *ortho*-C–H nitration of arenes with *tert*-butyl nitrite and toluene as the radical precursors was demonstrated (Liang et al. 2015). The azaindole-assisted *ortho*-C–H nitration of arenes with *tert*-butyl nitrite affording various nitrated azaindole derivatives was disclosed (Chun et al. 2018).

Despite the compelling progress on the C–H nitration reaction of various *N*-heterocycles, the site-selective nitration reaction of quinoxalinones and pyrazinones under milder reaction conditions is still unexplored. Driven by our ongoing interest in the C−H functionalization of *N*-heterocycles (Han et al. 2018; Ghosh et al. 2019, 2021; An et al. 2020; Park et al. 2021), we herein describe the metal-free and site-selective C–H nitration reaction of quinoxalinones and pyrazinones with *tert*-butyl nitrite as a readily available nitrating agent. Notably, the gram-scale reaction, selective reduction of a nitro group, and thiocarbonylation demonstrate the synthetic utility of the developed method.

## Materials and methods

### General methods

Commercially available reagents were used without additional purification, unless otherwise stated. Quinoxalinones (**1a–1m**) and 5-aryl pyrazinones (**4a–4j**) were prepared according to the reported literature (Ghosh et al. 2021; Guo et al. 2021). *t*-Butyl nitrite was purchased from Aldrich, Switzerland. All the reactions were performed in an oil bath by using hot plate magnetic stirrer (IKA universal, Guangzhou city, China). Sealed tubes were purchased from Fischer Scientific (13 × 100 mm, 1495925A; Mexico) and dried in oven for overnight and cooled at room temperature prior to use. Thin layer chromatography was carried out using plates coated with silica gel 60 F_254_ (Merck KGaA, 64271 Darmstadt, Germany). For flash column chromatography, silica gel 60 Å (230–400 mesh, Merck, Germany) was used. Nuclear magnetic resonance spectra (^1^H, ^13^C, and ^19^F NMR) were recorded on a Bruker Unity 400, 500, and 700 MHz spectrometers in CDCl_3_, CD_3_COCD_3_, and DMSO-d_6_ solution and chemical shifts are reported as parts per million (ppm). Resonance patterns are reported with the notations s (singlet), br (broad), d (doublet), t (triplet), q (quartet), sext (sextet), dd (doublet of doublets), dt (doublet of triplets), dq (doublet of quartets), qd (quartet of doublets), td (triplet of doublets), tt (triplet of triplets), and m (multiplet). In addition, the notation br is used to indicate a broad signal. Coupling constants (*J*) are reported in hertz (Hz). IR spectra were recorded on a Varian 2000 Infrared spectrophotometer and are reported as cm^−1^. High-resolution mass spectra (HRMS) were recorded on a JEOL JMS-600 spectrometer.

### General procedure and characterization data for the C7-nitration of quinoxalinones (3a–3m)

To an oven-dried sealed tube charged with 1-methylquinoxalin-2(1*H*)-one (**1a**) (32.0 mg, 0.2 mmol, 100 mol %) was added *t*-butyl nitrite (**2a**) (71.4 µL, 0.6 mmol, 300 mol %) and CH_3_CN (2 mL) under O_2_ atmosphere at room temperature. After using O_2_ balloon, the reaction mixture was allowed to stir at 60 °C for 20 h. The reaction mixture was cooled to room temperature, diluted with EtOAc (4 mL) and concentrated in vacuo. The residue was purified by flash column chromatography (*n*-hexanes/EtOAc = 10:1 to 2:1) to afford **3a** (31.2 mg) in 76% yield.

#### 1-Methyl-7-nitroquinoxalin-2(1H)-one (3a)

31.2 mg (76%); eluent (*n*-hexanes/EtOAc = 10:1 to 2:1); brown solid; mp = 228.9–231.1 °C; ^1^H NMR (400 MHz, DMSO-d_6_) δ 8.43 (s, 1H), 8.32 (d, *J* = 2.4 Hz, 1H), 8.16 (dd, *J* = 8.8, 2.4 Hz, 1H), 8.06 (d, *J* = 8.8 Hz, 1H), 3.68 (s, 3H); ^13^C NMR (100 MHz, DMSO-d_6_) δ 154.1, 153.9, 147.9, 136.0, 133.9, 130.9, 117.7, 110.6, 28.9; IR (KBr) υ 2924, 2854, 1666, 1587, 1512, 1462, 1354 cm^−1^; HRMS (quadrupole, EI) m/z: [M]^+^ Calcd for C_9_H_7_N_3_O_3_ 205.0487; Found 205.0486.

#### 1-Ethyl-7-nitroquinoxalin-2(1H)-one (3b)

32.9 mg (75%); eluent (*n*-hexanes/acetone = 10:1 to 1:1); brown solid; mp = 165.5–167.9 °C; ^1^H NMR (400 MHz, CDCl_3_) δ 8.41 (s, 1H), 8.24 (d, *J* = 2.4 Hz, 1H), 8.18 (dd, *J* = 8.8, 2.4 Hz, 1H), 8.04 (d, *J* = 8.8 Hz, 1H), 4.36 (q, *J* = 7.2 Hz, 2H), 1.43 (t, *J* = 7.2 Hz, 3H); ^13^C NMR (100 MHz, CDCl_3_) δ 154.1, 153.9, 148.7, 136.9, 132.8, 132.1, 118.1, 109.7, 37.8, 12.6; IR (KBr) υ 2989, 2924, 1668, 1589, 1523, 1471, 1442, 1344, 1244, 1103 cm^−1^; HRMS (quadrupole, EI) m/z: [M]^+^ Calcd for C_10_H_9_N_3_O_3_ 219.0644; Found 219.0639.

#### 1-Isobutyl-7-nitroquinoxalin-2(1H)-one (3c)

36.6 mg (74%); eluent (*n*-hexanes/acetone = 10:1 to 1:1); light brown solid; mp = 121.8–124.0 °C; ^1^H NMR (400 MHz, CDCl_3_) δ 8.41 (s, 1H), 8.21 (d, *J* = 2.4 Hz, 1H), 8.15 (dd, *J* = 8.8, 2.4 Hz, 1H), 8.02 (d, *J* = 8.8 Hz, 1H), 4.15 (d, *J* = 7.6 Hz, 2H), 2.27 (sep, *J* = 6.4 Hz, 1H), 1.04 (s, 3H), 1.02 (s, 3H); ^13^C NMR (100 MHz, CDCl_3_) δ 154.8, 153.9, 148.5, 136.9, 133.4, 132.0, 118.1, 110.3, 49.3, 27.4, 20.2; IR (KBr) υ 3114, 2956, 2927, 2871, 1664, 1591, 1562, 1522, 1464, 1441, 1338, 1315, 1236, 1132, 1099, 1057 cm^−1^; HRMS (quadrupole, EI) m/z: [M]^+^ Calcd for C_12_H_13_N_3_O_3_ 247.0957; Found 247.0954.

#### 1-Butyl-7-nitroquinoxalin-2(1H)-one (3d)

35.2 mg (71%); eluent (*n*-hexanes/acetone = 10:1 to 3:1); yellow solid; mp = 112.3–114.3 °C; ^1^H NMR (400 MHz, CDCl_3_) δ 8.40 (s, 1H), 8.22 (d, *J* = 2.0 Hz, 1H), 8.16 (dd, *J* = 8.8, 2.0 Hz, 1H), 8.03 (d, *J* = 8.4 Hz, 1H), 4.28 (dd, *J* = 6.0 Hz, 2H), 1.80–1.73 (m, 2H), 1.51 (sext, *J* = 7.2 Hz, 2H), 1.02 (t, *J* = 7.6 Hz, 3H); ^13^C NMR (100 MHz, CDCl_3_) δ 154.3, 153.8, 148.6, 136.9, 133.1, 132.0, 118.1, 109.9, 42.5, 29.5, 20.3, 13.8; IR (KBr) υ 2956, 2925, 1668, 1593, 1527, 1462, 1346, 1265 cm^−1^; HRMS (quadrupole, EI) m/z: [M]^+^ Calcd for C_12_H_13_N_3_O_3_ 247.0957; Found 247.0954.

#### 1-(4-Bromobutyl)-7-nitroquinoxalin-2(1H)-one (3e)

51.2 mg (78%); eluent (*n*-hexanes/acetone = 10:1 to 1:1); yellow solid; mp = 126.3–128.5 °C; ^1^H NMR (400 MHz, CDCl_3_) δ 8.42 (s, 1H), 8.26 (d, *J* = 2.4 Hz, 1H), 8.18 (dd, *J* = 8.4, 2.0 Hz, 1H), 8.05 (d, *J* = 8.4 Hz, 1H), 4.33 (t, *J* = 7.6 Hz, 2H), 3.50 (t, *J* = 5.6 Hz, 2H), 2.08–1.95 (m, 4H); ^13^C NMR (100 MHz, CDCl_3_) δ 154.3, 153.7, 148.7, 136.9, 132.9, 132.2, 118.3, 109.7, 41.6, 32.6, 29.6, 25.9; IR (KBr) υ 2922, 2854, 1666, 1593, 1564, 1525, 1444, 1344, 1317, 1103 cm^−1^; HRMS (quadrupole, EI) m/z: [M]^+^ Calcd for C_12_H_12_BrN_3_O_3_ 325.0062; Found 325.0061.

#### 1-Benzyl-7-nitroquinoxalin-2(1H)-one (3f)

30.9 mg (55%); eluent (*n*-hexanes/EtOAc = 10:1 to 2:1); light brown solid; mp = 165.7–167.9 °C; ^1^H NMR (400 MHz, CDCl_3_) δ 8.52 (s, 1H), 8.23 (d, *J* = 2.0 Hz, 1H), 8.12 (dd, *J* = 8.8, 2.0 Hz, 1H), 8.03 (d, *J* = 8.4 Hz, 1H), 7.38–1.28 (m, 5H), 5.51 (s, 2H); ^13^C NMR (100 MHz, CDCl_3_) δ 154.6, 153.9, 148.5, 136.9, 134.2, 133.1, 131.9, 129.5, 128.5, 127.3, 118.4, 110.6, 46.1; IR (KBr) υ 2922, 2854, 1666, 1593, 1564, 1523, 1450, 1342, 1317, 1219, 1103 cm^−1^; HRMS (quadrupole, EI) m/z: [M]^+^ Calcd for C_15_H_11_N_3_O_3_ 281.0800; Found 281.0800.

#### 3-((7-Nitro-2-oxoquinoxalin-1(2H)-yl)methyl)benzonitrile (3g)

19.2 mg (31%); eluent (*n*-hexanes/acetone = 10:1 to 2:1); yellow solid; mp = 193.8–196.1 °C; ^1^H NMR (400 MHz, CDCl_3_) δ 8.53 (s, 1H), 8.17 (dd, *J* = 5.2, 1.2 Hz, 1H), 8.09 (d, *J* = 0.8 Hz, 1H), 8.08 (d, *J* = 2.8 Hz, 1H), 7.61 (dt, *J* = 4.4 Hz, 1H), 7.60–7.58 (m, 1H), 7.54–7.53 (m, 1H), 7.51 (t, *J* = 4.4 Hz, 1H), 5.53 (s, 2H); ^13^C NMR (100 MHz, CDCl_3_) δ 154.4, 153.7, 148.7, 136.9, 135.8, 132.8, 132.4, 132.3, 131.7, 130.6, 130.3, 118.8, 118.1, 113.9, 109.9, 45.4; IR (KBr) υ 2924, 2854, 2231, 1670, 1593, 1566, 1525, 1448, 1344 cm^−1^; HRMS (quadrupole, EI) m/z: [M]^+^ Calcd for C_16_H_10_N_4_O_3_ 306.0753; Found 306.0748.

#### 1-(4-Ethoxyphenyl)-7-nitroquinoxalin-2(1H)-one (3h)

34.4 mg (56%); eluent (*n*-hexanes/EtOAc = 10:1 to 2:1); orange solid; mp = 182.0–183.9 °C; ^1^H NMR (700 MHz, CD_3_COCD_3_) δ 8.40 (s, 1H), 8.14 (dd, *J* = 8.4, 2.1 Hz, 1H), 8.09 (d, *J* = 8.4 Hz, 1H), 7.56 (d, *J* = 2.8 Hz, 1H), 7.39 (dt, *J* = 9.1, 2.8 Hz, 2H), 7.20 (dt, *J* = 9.1, 3.5 Hz, 2H), 4.18 (q, *J* = 7.0 Hz, 2H), 1.44 (t, *J* = 7.0 Hz, 3H); ^13^C NMR (175 MHz, CD_3_COCD_3_) δ 160.9, 155.6, 155.1, 149.1, 137.3, 136.6, 131.9, 130.5, 128.2, 118.4, 116.8, 111.8, 64.6, 15.1; IR (KBr) υ 2925, 2854, 1676, 1593, 1523, 1508, 1477, 1435, 1344, 1302, 1246, 1043 cm^−1^; HRMS (quadrupole, EI) m/z: [M]^+^ Calcd for C_16_H_13_N_3_O_4_ 311.0906; Found 311.0904.

#### 7-Nitro-1-(4-nitrophenyl)quinoxalin-2(1H)-one (3i)

18.8 mg (30%); eluent (*n*-hexanes/EtOAc = 10:1 to 3:1); yellow solid; mp = 244.2–245.6 °C; ^1^H NMR (400 MHz, CDCl_3_) δ 8.55 (dt, *J* = 8.8, 2.8 Hz, 2H), 8.52 (s, 1H), 8.20 (dd, *J* = 8.8, 2.4 Hz, 1H), 8.12 (d, *J* = 8.8 Hz, 1H), 7.58–7.54 (m, 3H); ^13^C NMR (100 MHz, CDCl_3_) δ 154.0, 153.6, 148.9, 148.6, 139.6, 136.3, 133.6, 132.1, 129.8, 126.3, 119.2, 110.7; IR (KBr) υ 3111, 3082, 2924, 1680, 1595, 1523, 1435, 1348 cm^−1^; HRMS (quadrupole, EI) m/z: [M]^+^ Calcd for C_14_H_8_N_4_O_5_ 312.0495; Found 312.0490.

#### 7-Nitro-1-(m-tolyl)quinoxalin-2(1H)-one (3j)

33.8 mg (60%); eluent (*n*-hexanes/EtOAc = 10:1 to 3:1); yellow solid; mp = 183.1–185.6 °C; ^1^H NMR (400 MHz, CDCl_3_) δ 8.51 (s, 1H), 8.14 (dd, *J* = 8.8, 2.4 Hz, 1H), 8.07 (d, *J* = 8.8 Hz, 1H), 7.60 (d, *J* = 2.4 Hz, 1H), 7.56 (td, *J* = 8.0, 1.6 Hz, 1H), 7.43 (d, *J* = 7.6 Hz, 1H), 7.10–7.08 (m, 2H), 2.47 (s, 3H); ^13^C NMR (100 MHz, CDCl_3_) δ 154.4, 154.3, 148.5, 141.4, 136.3, 134.7, 134.1, 131.5, 130.8, 128.5, 124.9, 119.9, 118.5, 111.6, 21.6; IR (KBr) υ 2924, 2854, 1678, 1593, 1562, 1527, 1433, 1344 cm^−1^; HRMS (quadrupole, EI) m/z: [M]^+^ Calcd for C_15_H_11_N_3_O_3_ 281.0800; Found 281.0803.

#### Ethyl 3-(7-nitro-2-oxoquinoxalin-1(2H)-yl)benzoate (3k)

29.2 mg (43%); eluent (*n*-hexanes/acetone = 10:1 to 2:1); yellow oil; ^1^H NMR (400 MHz, CD_3_COCD_3_) δ 8.45 (s, 1H), 8.28 (dt, *J* = 7.6, 1.6 Hz, 1H), 8.19–8.12 (m, 3H), 7.88 (t, *J* = 7.6 Hz, 1H), 7.81 (dq, *J* = 7.6, 1.2 Hz, 1H), 7.47 (d, *J* = 2.4 Hz, 1H), 7.47 (qd, *J* = 7.2, 1.6 Hz, 2H), 1.36 (t, *J* = 7.2 Hz, 3H); ^13^C NMR (100 MHz, CDCl_3_) δ 165.7, 155.5, 154.9, 149.1, 137.2, 136.4, 135.9, 134.1, 133.9, 132.1, 131.7, 131.6, 130.6, 118.7, 111.5, 62.0, 14.5; IR (KBr) υ 3066, 2924, 2854, 1716, 1680, 1593, 1562, 1525, 1436, 1344, 1269, 1211, 1182, 1103, 1082, 1022 cm^−1^; HRMS (quadrupole, EI) m/z: [M]^+^ Calcd for C_17_H_13_N_3_O_5_ 339.0855; Found 339.0852.

#### 6-Bromo-1-methyl-7-nitroquinoxalin-2(1H)-one (3l)

19.9 mg (35%); eluent (*n*-hexanes/EtOAc = 10:1 to 2:1); light brown solid; mp = 229.4–231.2 °C; ^1^H NMR (400 MHz, CD_3_COCD_3_) δ 8.33 (s, 1H), 8.24 (s, 1H), 8.16 (s, 1H), 3.72 (s, 3H); ^13^C NMR (100 MHz, CD_3_COCD_3_) δ 155.3, 154.9, 147.8, 135.9, 134.9, 112.8, 106.2, 23.3; IR (KBr) υ 2922, 2852, 1664, 1585, 1554, 1533, 1456, 1344 cm^−1^; HRMS (quadrupole, EI) m/z: [M]^+^ Calcd for C_9_H_6_BrN_3_O_3_ 282.9593; Found 282.9593.

#### 7-Chloro-1-methyl-5-nitroquinoxalin-2(1H)-one (3m)

21.6 mg (45%); eluent (*n*-hexanes/EtOAc = 10:1 to 3:1); light brown solid; mp = 229.4–231.5 °C; ^1^H NMR (700 MHz, CD_3_COCD_3_) δ 8.24 (s, 1H), 7.91 (d, *J* = 1.2 Hz, 1H), 7.83 (d, *J* = 1.2 Hz, 1H), 3.74 (s, 3H); ^13^C NMR (175 MHz, CD_3_COCD_3_) δ 154.9, 153.5, 150.2, 137.0, 136.7, 124.3, 118.3, 117.4, 23.3; IR (KBr) υ 2918, 2861, 1676, 1603, 1537, 1454, 1379, 1267 cm^−1^; HRMS (quadrupole, EI) m/z: [M]^+^ Calcd for C_9_H_6_ClN_3_O_3_ 239.0098; Found 239.0096.

### General procedure and characterization data for the C3-nitration of 5-aryl pyrazinones (5a–5j)

To an oven-dried sealed tube charged with 1-methyl-5-phenylpyrazin-2(1*H*)-one (**4a**) (37.2 mg, 0.2 mmol, 100 mol %) was added *t*-butyl nitrite (**2a**) (119.0 µL, 1.0 mmol, 500 mol %) and CH_3_CN (2.5 mL) under O_2_ atmosphere at room temperature. After using O_2_ balloon, the reaction mixture was allowed to stir at 60 °C for 28 h. The reaction mixture was cooled to room temperature, diluted with EtOAc (4 mL) and concentrated in vacuo. The residue was purified by flash column chromatography (*n*-hexane/acetone = 10:1 to 3:1) to afford **5a** (33.4 mg) in 72% yield.

#### 1-Methyl-3-nitro-5-phenylpyrazin-2(1H)-one (5a)

33.4 mg (72%); eluent (*n*-hexane/acetone = 10:1 to 3:1); yellow solid; mp = 191.2–193.8 °C; ^1^H NMR (700 MHz, DMSO-d_6_) δ 8.93 (s, 1H), 7.83–7.81 (m, 2H), 7.50–7.47 (m, 2H), 7.39 (tt, *J* = 7.7, 0.7 Hz, 1H), 3.69 (s, 3H); ^13^C NMR (125 MHz, DMSO-d_6_) δ 148.4, 148.1, 135.9, 133.6, 128.9, 128.4, 127.9, 124.6, 38.3; IR (KBr) υ 3060, 2927, 1674, 1604, 1543, 1493, 1419, 1267 cm^−1^; HRMS (quadrupole, EI) m/z: [M]^+^ Calcd for C_11_H_9_N_3_O_3_ 231.0644; Found 231.0641.

#### 1-Methyl-3-nitro-5-(4-(trifluoromethoxy)phenyl)pyrazin-2(1H)-one (5b)

32.2 mg (51%); eluent (*n*-hexanes/acetone = 10:1 to 3:1); yellow oil; ^1^H NMR (500 MHz, CDCl_3_) δ 7.90 (s, 1H), 7.74 (dt, *J* = 7.2, 2.4 Hz, 2H), 7.31–7.28 (m, 2H), 3.79 (s, 3H); ^13^C NMR (125 MHz, CDCl_3_) δ 150.2, 149.9, 148.3, 131.8, 131.4, 129.2, 129.1 (q, *J*_C-F_ = 33.0 Hz), 126.8, 121.7, 39.1; ^19^F NMR (470 MHz, CD_3_COCD_3_) δ − 58.5 (s); IR (KBr) υ 3060, 2925, 1680, 1608, 1545, 1500, 1346, 1263, 1110 cm^−1^; HRMS (quadrupole, EI) m/z: [M]^+^ Calcd for C_12_H_8_F_3_N_3_O_4_ 315.0467; Found 315.0462.

#### 5-(4-Chlorophenyl)-1-methyl-3-nitropyrazin-2(1H)-one (5c)

23.9 mg (45%); eluent (*n*-hexanes/acetone = 10:1 to 3:1); yellow solid; mp = 238.1–241.4 °C; ^1^H NMR (400 MHz, CD_3_COCD_3_) δ 8.78 (s, 1H), 7.86 (dt, *J* = 8.8, 2.8 Hz, 2H), 7.50 (dt, *J* = 8.4, 2.8 Hz, 2H), 3.81 (s, 3H); ^13^C NMR (100 MHz, CD_3_COCD_3_) δ 159.5, 135.5, 134.7, 133.8, 129.9, 128.3, 127.3, 105.0, 38.8; IR (KBr) υ 2925, 2856, 1678, 1606, 1543, 1489, 1340, 1267, 1196, 1086, 739 cm^−1^; HRMS (quadrupole, EI) m/z: [M]^+^ Calcd for C_11_H_8_ClN_3_O_3_ 265.0254; Found 265.0250.

#### 1-Methyl-3-nitro-5-(3-(trifluoromethyl)phenyl)pyrazin-2(1H)-one (5d)

34.2 mg (57%); eluent (*n*-hexanes/acetone = 10:1 to 3:1); yellow solid; mp = 159.2–161.2 °C; ^1^H NMR (500 MHz, CD_3_COCD_3_) δ 8.93 (s, 1H), 8.17–8.13 (m, 2H), 7.73 (dd, *J* = 3.5, 1.5 Hz, 2H), 3.83 (s, 3H); ^13^C NMR (125 MHz, CD_3_COCD_3_) δ 150.7, 149.3, 142.5, 136.2, 131.6 (q, *J*_C-F_ = 31.2 Hz), 130.9, 129.4, 127.8, 126.3 (q, *J*_C-F_ = 270.5 Hz), 125.7 (q, *J*_C-F_ = 3.2 Hz), 122.2 (d, *J*_C-F_ = 3.2 Hz), 38.9; ^19^F NMR (470 MHz, CD_3_COCD_3_) δ − 63.1 (s); IR (KBr) υ 3060, 2927, 1680, 1608, 1545, 1325, 1271, 1180, 1036 cm^−1^; HRMS (quadrupole, EI) m/z: [M]^+^ Calcd for C_12_H_8_F_3_N_3_O_3_ 299.0518; Found 299.0515.

#### 1-(Methoxymethyl)-3-nitro-5-phenylpyrazin-2(1H)-one (5e)

27.8 mg (53%); eluent (*n*-hexanes/acetone = 10:1 to 1:1); yellow oil; ^1^H NMR (500 MHz, CD_3_COCD_3_) δ 8.06 (s, 1H), 7.88–7.85 (m, 2H), 7.50–7.46 (m, 2H), 7.39 (tt, *J* = 7.0, 1.5 Hz, 1H), 5.54 (s, 2H), 3.52 (s, 3H); ^13^C NMR (125 MHz, CD_3_COCD_3_) δ 149.1, 134.8, 131.6, 130.1, 129.9, 129.5, 125.9, 124.4, 80.9, 58.3; IR (KBr) υ 2924, 2854, 1684, 1604, 1545, 1460, 1273 cm^−1^; HRMS (quadrupole, EI) m/z: [M]^+^ Calcd for C_12_H_11_N_3_O_4_ 261.0750; Found 261.0747.

#### 1-Benzyl-5-(2-methoxyphenyl)-3-nitropyrazin-2(1H)-one (5f)

30.4 mg (45%); eluent (*n*-hexanes/acetone = 10:1 to 1:1); yellow oil; ^1^H NMR (400 MHz, CD_3_COCD_3_) δ 8.20 (s, 1H), 7.62 (dd, *J* = 7.6, 1.6 Hz, 1H), 7.58 (ddd, *J* = 9.2, 7.6, 2.0 Hz, 1H), 7.43–7.41 (m, 2H), 7.35 (td, *J* = 7.2, 2.0 Hz, 2H), 7.27 (tt, *J* = 6.8, 1.6 Hz, 1H), 7.14 (d, *J* = 8.4 Hz, 1H), 7.07 (td, *J* = 7.6, 0.8 Hz, 1H), 4.54 (d, *J* = 6.0 Hz, 2H), 3.73 (s, 3H); ^13^C NMR (100 MHz, CD_3_COCD_3_) δ 192.7, 165.9, 160.7, 140.0, 135.5, 131.1, 129.2, 128.6, 127.9, 126.1, 121.5, 113.3, 56.4, 43.2; IR (KBr) υ 3240, 3074, 2584, 2318, 1683, 1645, 1580, 1485, 1462, 1439, 1310, 1250, 1207, 1165, 1117, 1022, 928 cm^−1^; HRMS (quadrupole, EI) m/z: [M]^+^ Calcd for C_18_H_15_N_3_O_4_ 337.1063; Found 337.1062.

#### 2-((3-Nitro-2-oxo-5-phenylpyrazin-1(2H)-yl)methyl)benzonitrile (5g)

40.6 mg (61%); eluent (*n*-hexanes/acetone = 10:1 to 3:1); yellow oil; ^1^H NMR (400 MHz, CD_3_COCD_3_) δ 8.90 (s, 1H), 7.88–7.85 (m, 3H), 7.73–7.67 (m, 2H), 7.58 (td, *J* = 7.6, 2.0 Hz, 1H), 7.51–7.47 (m, 2H), 7.50 (tt, *J* = 7.2, 1.6 Hz, 1H), 5.66 (s, 2H); ^13^C NMR (100 MHz, CD_3_COCD_3_) δ 153.1, 148.9, 138.5, 134.8, 134.5, 134.3, 134.1, 130.4, 130.3, 129.9, 129.8, 129.5, 125.9, 117.9, 112.9, 53.7; IR (KBr) υ 2956, 2924, 2225, 1668, 1593, 1525, 1448, 1344, 1213 cm^−1^; HRMS (quadrupole, EI) m/z: [M]^+^ Calcd for C_18_H_12_N_4_O_3_ 332.0909; Found 332.0907.

#### 1-(4-Methoxyphenyl)-3-nitro-5-phenylpyrazin-2(1H)-one (5h)

33.2 mg (51%); eluent (*n*-hexanes/acetone = 10:1 to 3:1); yellow solid; mp = 149.4–150.6 °C; ^1^H NMR (400 MHz, CDCl_3_) δ 7.92 (s, 1H), 7.75–7.72 (m, 2H), 7.47–7.38 (m, 5H), 7.05 (dt, *J* = 8.8, 3.6 Hz, 2H), 3.88 (s, 3H); ^13^C NMR (100 MHz, CDCl_3_) δ 162.5, 160.9, 147.9, 133.1, 131.1, 130.5, 130.4, 129.3, 127.0, 125.3, 121.6, 115.2, 55.9; IR (KBr) υ 3060, 1682, 1606, 1512, 1467, 1265 cm^−1^; HRMS (quadrupole, EI) m/z: [M]^+^ Calcd for C_17_H_13_N_3_O_4_ 323.0906; Found 323.0906.

#### 1-(4-Acetylphenyl)-3-nitro-5-phenylpyrazin-2(1H)-one (5i)

30.2 mg (45%); eluent (*n*-hexanes/acetone = 10:1 to 4:1); yellow oil; ^1^H NMR (500 MHz, CD_3_COCD_3_) δ 8.63 (s, 1H), 8.21 (dt, *J* = 9.0, 2.0 Hz, 2H), 7.91 (dq, *J* = 8.5, 1.0 Hz, 2H), 7.88 (dt, *J* = 8.5, 2.5 Hz, 2H), 7.50–7.46 (m, 2H), 7.40 (tt, *J* = 7.5, 1.0 Hz, 1H), 2.68 (s, 3H); ^13^C NMR (125 MHz, CD_3_COCD_3_) δ 197.2, 148.6, 143.2, 138.9, 134.7, 132.9, 130.2, 130.1, 129.8, 129.5, 127.8 (two carbons overlap), 126.0, 26.9; IR (KBr) υ 2924, 2854, 1685, 1599, 1545, 1360, 1265, 1194 cm^−1^; HRMS (quadrupole, EI) m/z: [M]^+^ Calcd for C_18_H_13_N_3_O_4_ 335.0906; Found 335.0905.

#### 3-Nitro-5-phenyl-1-(m-tolyl)pyrazin-2(1H)-one (5j)

31.4 mg (51%); eluent (*n*-hexanes/acetone = 10:1 to 4:1); yellow solid; mp = 141.3–144.1 °C; ^1^H NMR (400 MHz, CD_3_COCD_3_) δ 8.56 (s, 1H), 7.93–7.90 (m, 2H), 7.53–7.52 (m, 1H), 7.50–7.49 (m, 2H), 7.47–7.44 (m, 2H), 7.39 (tt, *J* = 7.2, 1.2 Hz, 2H), 2.44 (s, 3H); ^13^C NMR (100 MHz, CD_3_COCD_3_) δ 150.2, 148.7, 140.4, 139.9, 134.8, 133.4, 131.2, 130.1, 129.8, 129.4, 127.7, 125.9, 124.8, 124.3, 21.2; IR (KBr) υ 3059, 2925, 2854, 1684, 1603, 1545, 1489, 1454, 1342, 1269, 1194 cm^−1^; HRMS (quadrupole, EI) m/z: [M]^+^ Calcd for C_17_H_13_N_3_O_3_ 307.0957; Found 307.0956.

### General procedure for the gram scale experiment of 1a

To an oven-dried round bottom flask charged with 1-methylquinoxalin-2(1*H*)-one (**1a**) (1.0 g, 6.3 mmol, 100 mol %) was added *t*-butyl nitrite (**2a**) (2.25 mL, 18.9 mmol, 300 mol %) and CH_3_CN (60 mL) under O_2_ atmosphere at room temperature. After using O_2_ balloon, the reaction mixture was allowed to stir at 60 °C for 20 h. The reaction mixture was cooled to room temperature, diluted with EtOAc (25 mL) and concentrated in vacuo. The residue was purified by flash column chromatography (*n*-hexanes/EtOAc = 10:1 to 2:1) to afford **3a** (0.69 g) in 68% yield.

### General procedure for the gram scale experiment of 4a

To an oven-dried sealed tube charged with 1-methyl-5-phenylpyrazin-2(1*H*)-one (**4a**) (1.0 g, 5.4 mmol, 100 mol %) was added *t*-butyl nitrite (**2a**) (3.2 mL, 27.0 mmol, 500 mol %) and CH_3_CN (65 mL) under O_2_ atmosphere at room temperature. After using O_2_ balloon, the reaction mixture was allowed to stir at 60 °C for 28 h. The reaction mixture was cooled to room temperature, diluted with EtOAc (25 mL) and concentrated in vacuo. The residue was purified by flash column chromatography (*n*-hexane/acetone = 10:1 to 3:1) to afford **5a** (0.81 g) in 65% yield.

### General procedure and characterization data for the reduction of nitro group on 3a

To an oven-dried sealed tube charged with 1-methyl-7-nitroquinoxalin-2(1*H*)-one (**3a**) (41.0 mg, 0.2 mmol, 100 mol %), iron (46.9 mg, 0.84 mmol, 420 mol %), ammonium chloride powder (71.7 mg, 1.34 mmol, 670 mol %) were added MeOH/THF/H_2_O (1:1:1, 4.5 mL) at room temperature. The reaction mixture was allowed to stir in an oil bath for 12 h at 60 °C. The reaction mixture was cooled to room temperature, filtered through Celite, rinsing with methanol, and the volatiles were removed under reduced pressure. The aqueous residue was diluted with water, saturated NaHCO_3_ solution, and extracted with EtOAc (3 × 15 mL). The combined organic layer was washed with brine, dried over MgSO_4_, and concentrated in vacuo. The residue was purified by flash column chromatography (*n*-hexanes/acetone = 10:1 to 2:1) to afford **6a** (22.8 mg) in 65% yield.

#### 7-Amino-1-methylquinoxalin-2(1H)-one (6a)

22.8 mg (65%); eluent (*n*-hexanes/acetone = 10:1 to 2:1); brown oil; ^1^H NMR (400 MHz, DMSO-d_6_) δ 7.74 (s, 1H), 7.44 (d, *J* = 8.4 Hz, 1H), 6.60 (dd, *J* = 8.8, 2.4 Hz, 1H), 6.47 (d, *J* = 2.4 Hz, 1H), 6.13 (brs, 2H), 3.46 (s, 3H); ^13^C NMR (100 MHz, DMSO-d_6_) δ 155.0, 152.1, 141.3, 135.4, 130.9, 125.2, 111.3, 95.6, 28.2; IR (KBr) υ 3367, 3197, 2924, 2854, 1732, 1604, 1535, 1462, 1379, 1342, 1267 cm^−1^; HRMS (quadrupole, EI) m/z: [M]^+^ Calcd for C_9_H_9_N_3_O 175.0746; Found 175.0744.

### General procedure and characterization data for the thiocarbonylation of 3a into 6b

To an oven-dried sealed tube charged with 1-methyl-7-nitroquinoxalin-2(1*H*)-one (**3a**) (41.0 mg, 0.2 mmol, 100 mol %) and Lawesson’s reagent (242.7 mg, 0.6 mmol, 300 mol %) was added toluene (2 mL) under air at room temperature. The reaction mixture was allowed to stir in an oil bath for 12 h at 120 °C. The reaction mixture was cooled to room temperature, diluted with EtOAc (5 mL) and concentrated in vacuo. The aqueous residue was extracted with EtOAc (3 × 15 mL). The combined organic layer was washed with brine, dried over MgSO_4_, and concentrated in vacuo. The residue was purified by flash column chromatography (*n*-hexanes/acetone = 10:1 to 1:2) to afford **6b** (25.8 mg) in 58% yield.

#### 1-Methyl-7-nitroquinoxaline-2(1H)-thione (6b)

25.8 mg (58%); eluent (*n*-hexanes/acetone = 10:1 to 1:2); orange oil; ^1^H NMR (400 MHz, CD_3_COCD_3_) δ 8.79 (s, 1H), 8.60 (d, *J* = 2.4 Hz, 1H), 8.27 (dd, *J* = 8.8, 2.4 Hz, 1H), 8.10 (d, *J* = 8.8 Hz, 1H), 4.30 (s, 3H); ^13^C NMR (100 MHz, CD_3_COCD_3_) δ 179.7, 160.1, 140.9, 132.3 (two carbons overlap), 120.5, 112.4 (two carbons overlap), 37.7; IR (KBr) υ 3060, 2924, 2854, 1684, 1550, 1516, 1460, 1350, 1267, 1103 cm^−1^; HRMS (quadrupole, EI) m/z: [M]^+^ Calcd for C_9_H_7_N_3_O_2_S 221.0259; Found 221.0256.

### General procedures for the control experiment using a radical scavenger TEMPO

To an oven-dried sealed tube charged with 1-methylquinoxalin-2(1*H*)-one (**1a**) (32.0 mg, 0.2 mmol, 100 mol %) and TEMPO (312.5 mg, 2.0 mmol, 10.0 equiv.) were added *t*-butyl nitrite (**2a**) (71.4 µL, 0.6 mmol, 300 mol %) and CH_3_CN (2 mL) under O_2_ atmosphere at room temperature. After using O_2_ balloon, the reaction mixture was allowed to stir in an oil bath for 8 h at 60 °C. The reaction mixture was cooled to room temperature, diluted with EtOAc (4 mL) and concentrated in vacuo. On TLC the desired product **3a** was not detected.

### General procedure and characterization data for the reaction of 1a and 2a with radical polymerization mediator 1,1-diphenylethylene (2b)

To an oven-dried sealed tube charged with 1-methylquinoxalin-2(1*H*)-one (**1a**) (32.0 mg, 0.2 mmol, 100 mol %) and 1,1-diphenylethylene (**2b**) (72.1 mg, 0.4 mmol, 200 mol %) were added *t*-butyl nitrite (**2a**) (71.4 µL, 0.6 mmol, 300 mol %) and CH_3_CN (2 mL) under O_2_ atmosphere at room temperature. After using O_2_ balloon, the reaction mixture was allowed to stir in an oil bath for 8 h at 60 °C. The reaction mixture was cooled to room temperature, diluted with EtOAc (5 mL) and concentrated in vacuo. The aqueous residue was extracted with EtOAc (3 × 15 mL). The combined organic layer was washed with brine, dried over MgSO_4_, and concentrated in vacuo. The residue was purified by flash column chromatography (*n*-hexanes/acetone = 10:1 to 4:1) to afford **7a** (25.2 mg) in 56% yield.

#### (2-Nitroethene-1,1-diyl)dibenzene (7a)

25.2 mg (56%); eluent (*n*-hexanes/acetone = 10:1 to 4:1); yellow oil; ^1^H NMR (400 MHz, CD_3_COCD_3_) δ 7.72 (s, 1H), 7.51–7.42 (m, 6H), 7.40–7.37 (m, 2H), 7.27–7.24 (m, 2H); ^13^C NMR (100 MHz, CD_3_COCD_3_) δ 149.9, 137.9, 136.9, 136.0, 131.6, 129.8, 129.7, 129.6, 129.5, 129.3; IR (KBr) υ 3059, 2925, 2854, 1610, 1574, 1510, 1495, 1444, 1308, 1267 cm^−1^; HRMS (quadrupole, EI) m/z: [M]^+^ Calcd for C_14_H_11_NO_2_ 225.0790; Found 225.0791.

## Results

Our optimization was performed by investigating the coupling reaction of 1-methylquinoxalin-2(1*H*)-one (**1a**) with *tert*-butyl nitrite (**2a**), as shown in Table 1.

**Table 1:** Selected optimization of the reaction conditions 
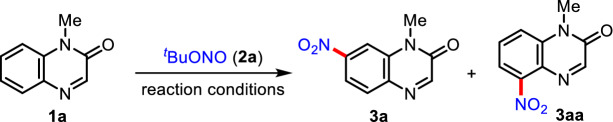

Entry	**2a** (Equiv.)	Additive (Equiv.)	*T* ^o^C	Solvent	Yield (%)^a^	
					**3a**	**3aa**
1	**2a** (2)	–	60	PhCl	12	N.R.
2	**2a** (2)	–	60	ClCH_2_CH_2_Cl	25	5
3	**2a** (2)	–	60	1,4-Dioxane	Trace	N.R.
4	**2a** (2)	–	60	CH_3_CN	40	8
5	**2a** (2)	K_2_S_2_O_8_ (1)	60	CH_3_CN	40	14
6	**2a** (2)	Na_2_S_2_O_8_ (1)	60	CH_3_CN	Trace	5
7	**2a** (2)	AgNO_2_ (1)	60	CH_3_CN	35	10
**8**	**2a (3)**	–	**60**	**CH**_**3**_**CN**	**76**	**9**
9	**2a** (3)	–	40	CH_3_CN	30	5
10	**2a** (3)	–	80	CH_3_CN	55	12
11^b^	**2a** (3)	–	60	CH_3_CN	22	4
12	**2a** (4)	–	60	CH_3_CN	68	9
13	**2a** (5)	–	60	CH_3_CN	65	9

Reaction conditions: **1a** (0.2 mmol), **2a** (quantity noted), additive (quantity noted), solvent (2 mL) under O_2_ atmosphere at indicated temperature for 20 h in reaction tubes

^a^Isolated yield by flash column chromatography

^b^The reaction was performed under N_2_ atmosphere. N.R. = no reaction

Entry 8 is the final optimized reaction conditions

**Fig. 3:**
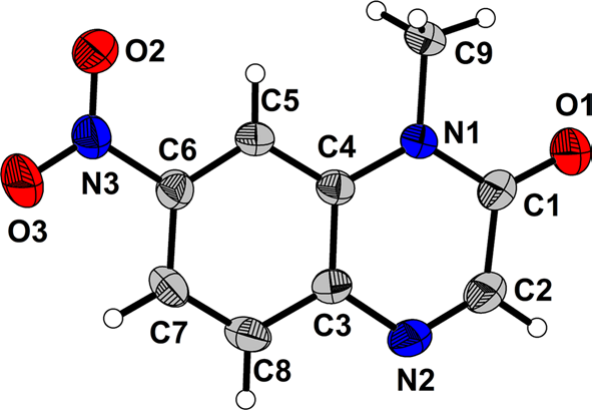
X-ray crystallographic data of **3a**

The nitration reaction of **1a** was initiated by using *tert*-butyl nitrite (**2a**) to deliver C7-nitrated quinoxalinone **3a** in 12% yield, and no formation of other regioisomers including C5-nitrated adduct **3aa** was observed (Table 1, entry 1). The chemical structure of C7-nitrated quinoxalinone **3a** (CCDC 2099185) was elucidated by the X-ray crystallographic analysis (Fig. 3).

Solvent screening revealed that this coupling reaction displayed the increased reactivity in CH_3_CN solvent to give **3a** in 40% yield (Table 1, entries 2 − 4). Addition of oxidants such as K_2_S_2_O_8_, Na_2_S_2_O_8_, and AgNO_2_ were found to be unsatisfactory in this transformation (Table 1, entries 5 − 7). To our delight, this reaction smoothly proceeded with three equiv. of **2a** to afford the desired product **3a** in 76% yield along with C5-nitrated compound **3aa** in 9% yield (Table 1, entry 8). The reaction temperature is quite pivotal for this transformation, as shown in entries 9 and 10. It should be noted that molecular oxygen was needed for the formation of both **3a** and **3aa** (Table 1, entry 11), revealing that a NO radical, derived from the decomposition of *tert*-butyl nitrite, could be readily oxidized into a reactive NO_2_ radical by molecular oxygen (O_2_ gas). Finally, when the reaction was performed with increased loading of **2a**, the lower formation of our desired product was observed (Table 1, entries 12 and 13).

With the optimal reaction conditions in hand, the scope of quinoxalinones was examined as shown in Table 2. The linear and branched *N*-alkylated quinoxalinones **1b** − **1e** were found to be suitable substrates for this coupling reaction to afford C7-nitrated quinoxalinones **3b** − **3e** in high yields. It is noteworthy that a linear alkyl halide **1e** was completely compatible under the current reaction conditions, and the tolerance of bromo moiety presents valuable opportunities for further versatile synthetic transformations. In addition, *N*-benzylated quinoxalinones **1f** and **1g** were also coupled with **2a** to provide the corresponding products **3f** (55%) and **3g** (31%). To our pleasure, the current protocol could be applied to *N*-arylated quinoxalinones **1h** − **1k**, producing the desired products **3h** − **3k** without undergoing the C−H nitration on the *N*-aryl ring. It is mentioned that electron-deficient NO_2_ (**1i**) and CO_2_Et (**1k**) groups on the *N*-aryl moiety were found to be comparatively less reactive in this transformation, presumably due to the destabilization of radical and carbocation intermediates. To observe the steric and electronic effects on the quinoxalinone framework, the reactions of **1l** with **2a** under the standard and modified reaction conditions were subjected to afford the C7-nitrated adduct **3l** in 20% and 35% yields. It should be mentioned that the nitration reaction of C7-substituted quinoxalinone **1m** preferentially occurred at the C5-position, affording the nitrated product **3m** as a single regioisomer in 45% yield.

**Table 2:**
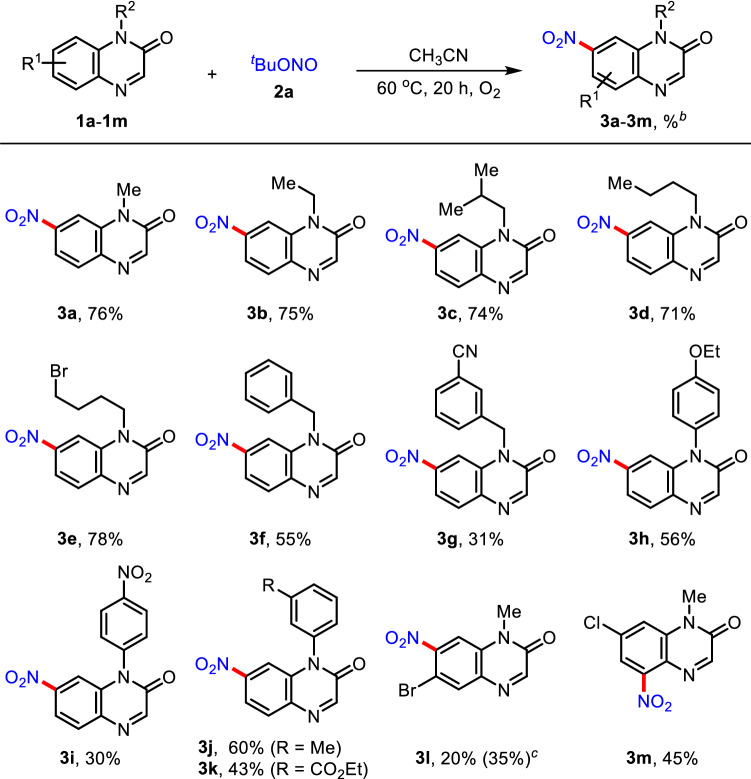
Scope of quinoxalinones^a^

^a^Reaction conditions: **1a** − **1m** (0.2 mmol), **2a** (0.6 mmol, 3 equiv.), CH_3_CN (2 mL) under O_2_ atmosphere at 60 °C for 20 h in reaction tubes

^b^Isolated yield by flash column chromatography

^c^**2a** (1.0 mmol, 5 equiv.) was used

With successfully screening results of quinoxalinone substrates, the substrate scope of various 5-aryl pyrazin-2-ones **4a** − **4j** was evaluated, as shown in Table 3. The reaction of 5-phenyl pyrazinone **4a** with **2a** under the modified reaction conditions (5 equiv. of **2a**, 28 h) provided **5a** in 72% yield. Additionally, *N*-alkyl-5-aryl-substituted pyrazinones **4b**–**4e** reacted with **2a** to afford C3-nitrated pyrazinone adducts **5b**–**5e** in moderate to good yields. In addition, *N*-benzyl-substituted pyrazinones **4f** and **4g** were also compatible under the current reaction conditions to give the corresponding products **5f** (45%) and **5g** (61%). The complete regioselectivity was observed in all cases. Finally, the C3-nitration reaction of *N*-aryl-substituted pyrazinones **4h**–**4j** smoothly proceeded, resulting in the formation of the desired products **5h**–**5j**. The functional group compatibility of nitrile and acetyl moieties (**5g** and **5i**) allows further synthetic elaboration of the products.

**Table 3:**
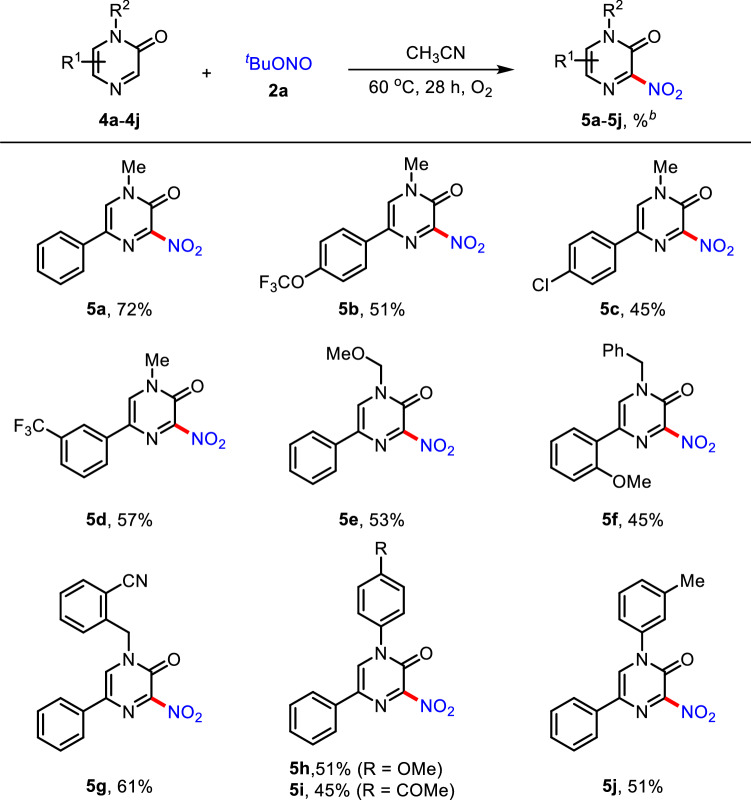
Scope of 5-aryl pyrazin-2-ones^a^

^a^Reaction conditions: **4a** − **4j** (0.2 mmol), **2a** (1.0 mmol, 5 equiv.), CH_3_CN (2.5 mL) under O_2_ atmosphere at 60 °C for 28 h in reaction tubes

^b^Isolated yield by flash column chromatography

**Fig. 4:**
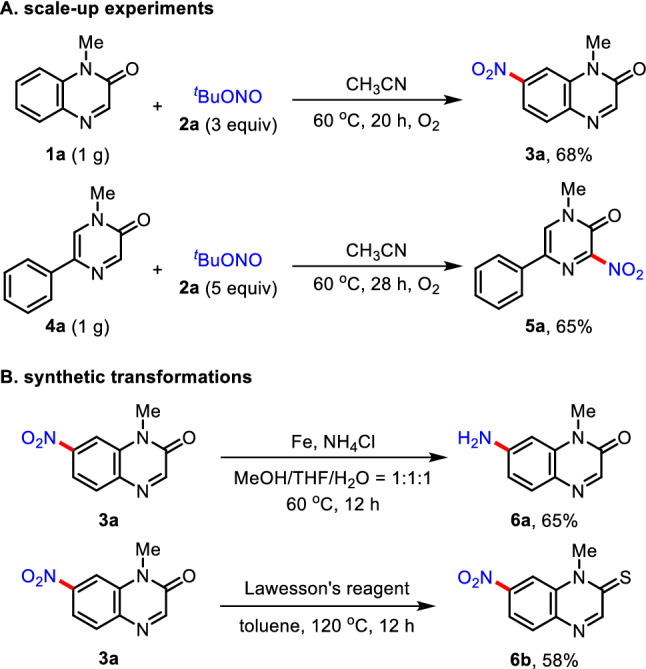
Scale-up experiments and synthetic transformations

To demonstrate the robustness and practicality of this process, the scale-up experiments and synthetic transformations were performed (Fig. 4). The nitration reaction of **1a** was readily scaled up to 1 g (6.3 mmol) for the formation of **3a** (0.69 g) in 68% yield. In addition, the gram-scale reaction of **4a** (1 g, 5.4 mmol) with **2a** was successfully achieved to afford 0.81 g of **5a** in 65% yield. Meanwhile, the selective reduction of a nitro moiety of the product **3a** was performed by the single electron reduction protocol using by Fe/NH_4_Cl to furnish the desired aniline adduct **6a** in 65% yield. Moreover, treatment of **3a** with Lawesson’s reagent resulted in the formation of 7-nitroquinoxaline-2(1*H*)-thione **6b** in 58% yield.

## Discussion

**Fig. 5:**
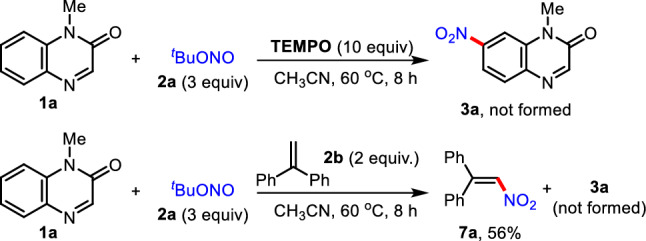
Mechanistic investigations

To support the mechanistic pathway for this reaction, the nitration reaction of **1a** and **2a** was performed in the presence of a radical scavenger TEMPO (Fig. 5). No formation of C7-nitrated quinoxalinone **3a** was observed. The nitration reaction was completely inhibited by 1,1-diphenylethylene (**2b**) as a radical polymerization mediator, and 1,1-diphenyl-2-nitroethylene (**7a**) was obtained in 56% yield. These results support that a radical pathway is involved in this process.

**Fig. 6:**
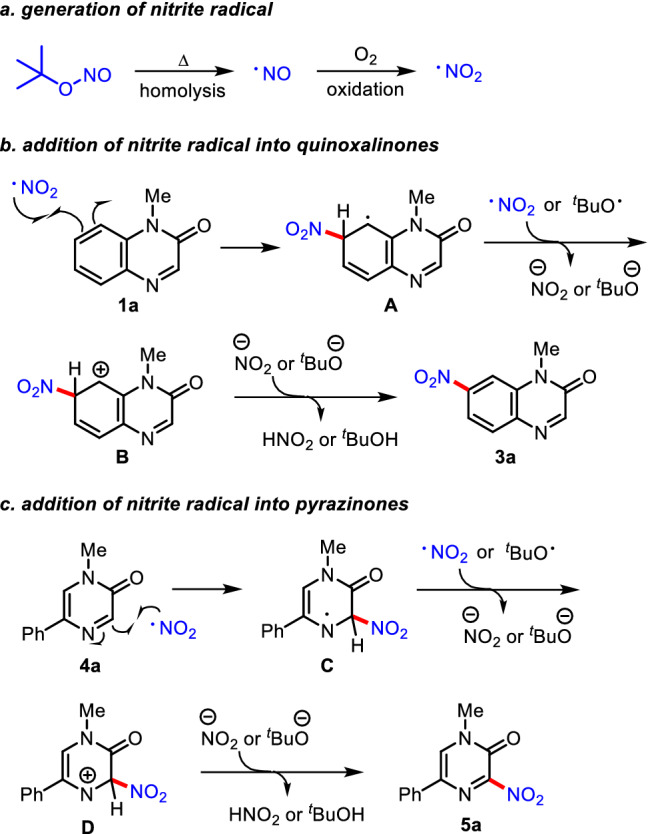
Proposed reaction mechanism for the site-selective C–H nitration

Based on preliminary mechanistic investigation and reported literatures (Liang et al. 2015; Chun et al. 2018), a proposed reaction mechanism is outlined in Fig. 6. In the presence of molecular oxygen, a reactive NO_2_ radical can be derived from the thermal decomposition of ^*t*^BuONO, followed by subsequent aerobic oxidation of a NO radical. A NO_2_ radical can undergo the radical addition into the C7-position on quinoxalinone **1a**, affording intermediate **A**. The single-electron transfer (SET) process by the assistance of NO_2_ or ^*t*^BuO radical followed by aromatization provides the C7-nitrated product **3a**. In case of the nitration of pyrazinone **4a**, the radical addition can occur at the C3-position, delivering a nitrogen radical species **C**, which further undergoes the SET reaction and elimination reaction to produce **6a**. The site-selectivity between the C7- and C3-positions of quinoxalinone **1a** can be rationalized by the electronic density between the electron-rich aromatic ring and electron-deficient *N*-heterocycle ring in the electrophilic radical addition step. Moreover, the C7-selectivity over C6,C8-positions on aromatic ring of **1a** can be explained by the relative stability of a radical intermediate **A**. However, the site-selectivity between C7- and C5-position still remains unclear, and the detailed mechanistic investigations on the site-selectivity of this process are underway.

In summary, we described the synthesis of biologically relevant C7-nitrated quinoxalinones and C3-nitrated pyrazinones through metal-free C–H nitration with *t*-butyl nitrite. From the mechanistic point of view, the radical addition to quinoxalinones with *t*-butyl nitrite exclusively occurred at the electron-rich aromatic region beyond electron-deficient *N*-heterocycle ring. In contrast, the nitration reaction of pyrazinones readily takes place at the C3-position via the single electron transfer process of a nitrogen radical intermediate followed by elimination reaction. This protocol is characterized by the scale-up compatibility, mild reaction conditions, and excellent functional group tolerance. The selective reduction of a NO_2_ group and thiocarbonylation on the synthesized products highlight the importance of the developed methodology.

## Supplementary Information

Below is the link to the electronic supplementary material.

Supplementary file1 (PDF 3470 kb)
