# Correction to: Site-selective and metal-free C–H nitration of biologically relevant N-heterocycles

**DOI:** 10.1007/s12272-021-01362-2

**Published:** 2021-11-13

**Authors:** Junghyea Moon, Hyun Ku Ji, Nayoung Ko, Harin Oh, Min Seo Park, Suho Kim, Prithwish Ghosh, Neeraj Kumar Mishra, In Su Kim

**Affiliations:** School of Pharmacy, Sungkyunkwan University, Suwon, 16419 Republic of Korea

## Correction to: Archives of Pharmacal Research 10.1007/s12272-021-01351-5

The article **Site-selective and metal-free C–H nitration of biologically relevant N-heterocycles** written by Junghyea Moon, Hyun Ku Ji, Nayoung Ko, Harin Oh, Min Seo Park, Suho Kim, Prithwish Ghosh, Neeraj Kumar Mishra & In Su Kim was originally published Online First without Open Access. After publication the author decided to opt for Open Choice and to make the article an Open Access publication. Therefore, the copyright of the article has been changed to **©The Author(s)** and the article is forthwith distributed under the terms of the Creative Commons Attribution.

