# Supplementary material for: Site-selective and metal-free C–H nitration of biologically relevant N-heterocycles
Source: Arch Pharm Res. 2021 Oct 18;44(11):1012–23. doi: 10.1007/s12272-021-01351-5 (PMC8685193; doi:10.1007/s12272-021-01351-5)

Supplementary Material

Site-selective and metal-free C–H nitration of biologically relevant *N*-heterocycles

Junghyea Moon,† Hyun Ku Ji,† Nayoung Ko, Harin Oh, Min Seo Park, Suho Kim, Prithwish Ghosh, Neeraj Kumar Mishra,* and In Su Kim*

School of Pharmacy, Sungkyunkwan University, Suwon 16419, Republic of Korea

* Corresponding authors. neerajchemistry@gmail.com (N.K.M), insukim@skku.edu (I.S.K.)

‡ These authors equally contributed.

List of the Contents

X-ray crystallographic data of compound 3a -- S2–S11

^{1}H NMR and ^{13}C NMR spectra of all compounds -- S12–S37

^{19}F NMR of F-containing compounds 5b and 5d -- S38

X-ray crystallographic data of 3a (CCDC 2099185)

Sample preparation (solvent evaporation)

Compound **3a** (10 mg) was dissolved with 1 mL of $CHCl_3$ in opened inner vessel, and *n*-pentane (5 mL) as an anti-solvent has been employed in closed outer vessel. After vapor diffusion for 4 days, the single crystals of compound **3a** were obtained.

Detailed experimental description for the crystal measurement of 3a

Crystals grew as colorless plate-like in CH_2Cl_2 by slow evaporation from *n*-pentane. The crystal structures of compound **3a** were determined by standard crystallographic methods. A colorless crystal of $C_9H_7N_3O_3$ with approximate dimensions 0.020 x 0.100 x 0.100 mm^3 was used for single-crystal X-ray diffraction. The data were collected at 223(2) K using a Bruker D8 Venture equipped with a graphite monochromator with CuK_α radiation (λ = 0.71073 Å) and a PHOTON III M14 detector in Western Seoul Center of Korea Basic Science Institute. Data collection and integration were performed with SMART APEX3 software package (SAINT).[4] Absorption correction was performed by multi-scan method implemented in SADABS.[5] The structure was solved by direct methods and refined by full-matrix least-squares on F^2 using SHELXTL program package (version 6.14).[6] All the non-hydrogen atoms were refined anisotropically, and hydrogen atoms were added to their geometrically ideal positions.

Details of crystal data, data collection and structure refinement are listed in Table S2. Further details of the individual structures can be obtained from the Cambridge Crystallographic Data Centre by quoting **CCDC 2099185**.

ORTEP diagram of 3a (CCDC 2099185)

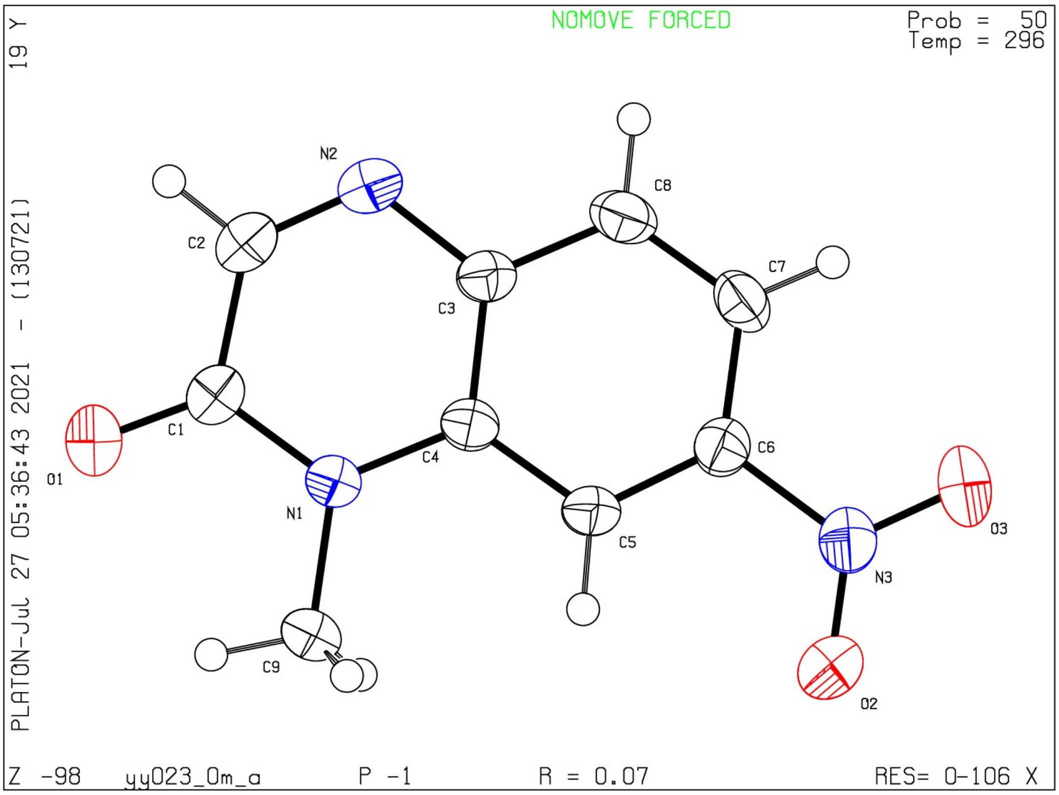

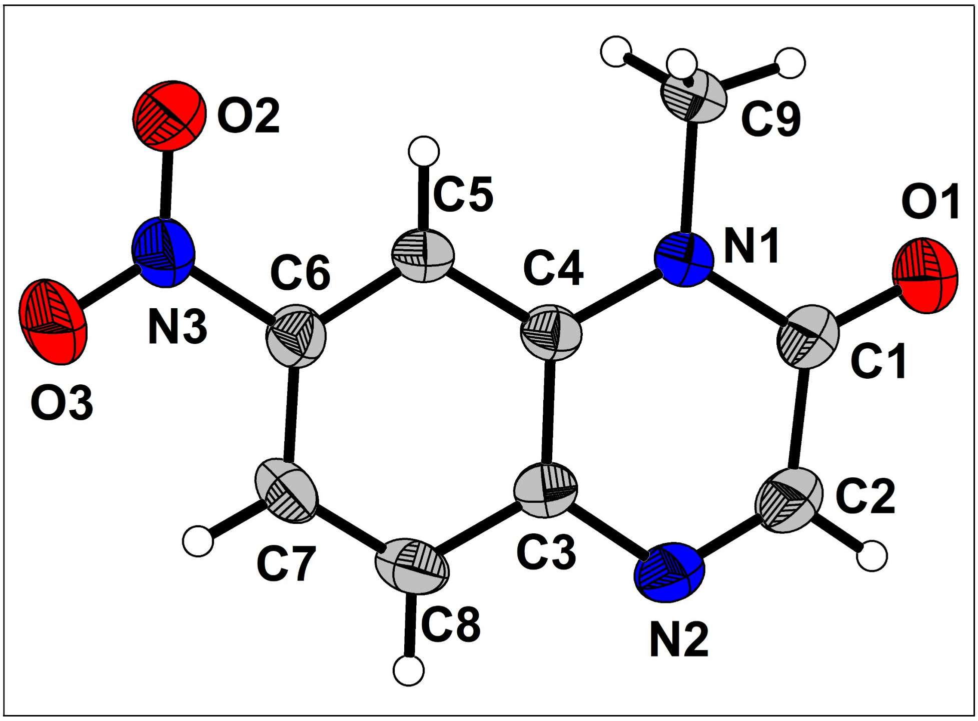

A colorless plate-like specimen of $C_9H_7N_3O_3$, approximate dimensions 0.020 m x 0.100 mm x 0.100 mm, was used for the X-ray crystallographic analysis. The X-ray intensity data were measured (λ = 0.71073 Å).

Table S1: Data collection details for 3a.

Axis	dx/mm	2θ/°	ω/°	φ/°	χ/°	Width/°	Frames	Time/s	Wavelength/Å	Voltage/kV	Current/mA	Temperature/K
Omega	60.587	18.54	-174.46	-156.00	54.74	1.00	206	10.00	0.71073	50	30.0	n/a
Omega	60.587	27.81	-165.19	0.00	54.74	1.00	206	10.00	0.71073	50	30.0	n/a
Omega	60.587	18.54	-174.46	102.00	54.74	1.00	206	10.00	0.71073	50	30.0	n/a
Phi	60.587	18.54	31.54	0.00	54.74	1.00	360	10.00	0.71073	50	30.0	n/a
Omega	60.587	18.54	-174.46	153.00	54.74	1.00	206	10.00	0.71073	50	30.0	n/a
Omega	60.587	18.54	-174.46	0.00	54.74	1.00	206	10.00	0.71073	50	30.0	n/a
Phi	60.587	0.00	0.00	0.00	54.74	360.00	1	108.00	0.71073	50	30.0	n/a

A total of 1391 frames were collected. The total exposure time was 3.89 hours. The frames were integrated with the Bruker SAINT software package using a narrow-frame algorithm. The integration of the data using a triclinic unit cell yielded a total of 15866 reflections to a maximum θ angle of 28.35 (0.75 Å resolution), of which 2190 were independent (average redundancy 7.245, completeness = 99.9%, R_{int} = 6.73%, R_{sig} = 4.78%) and 1364 (62.28%) were greater than $2\sigma(F^2)$. The final cell constants of a = 7.0535(7) Å, b = 7.1264(7) Å, c = 9.2329(9) Å, α = 77.936(3), β = 75.463(4), γ = 80.201(4), volume = 435.91(7) $Å^3$, are based upon the refinement of the XYZ-centroids of 2548 reflections above 20 σ(I) with 4.628 < 2θ < 54.07. Data were corrected for absorption effects using the Multi-Scan method (SADABS). The ratio of minimum to maximum apparent transmission was 0.865. The calculated minimum and maximum transmission coefficients (based on crystal size) are 0.9880 and 0.9980.

The structure was solved and refined using the Bruker SHELXTL Software Package, using the space group P -1, with Z = 2 for the formula unit, $C_9H_7N_3O_3$. The final anisotropic full-matrix least-squares refinement on F^2 with 137 variables converged at R1 = 7.10%, for the observed data and wR2 = 18.72% for all data. The goodness-of-fit was 1.038. The largest peak in the final difference electron density synthesis was 0.261 $e^-/Å^3$ and the largest hole was -0.321 $e^-/Å^3$ with an RMS deviation of 0.051 $e^-/Å^3$. On the basis of the final model, the calculated density was 1.563 g/cm^3 nd F(000), 212 e^-.

Table S2. Sample and crystal data for 3a.

**Chemical formula**	$C_9H_7N_3O_3$	
**Formula weight**	205.18 g/mol	
**Temperature**	296(2) K	
**Wavelength**	0.71073 Å	
**Crystal size**	0.020 x 0.100 x 0.100 mm	
**Crystal habit**	colorless plate	
**Crystal system**	triclinic	
**Space group**	P -1	
**Unit cell dimensions**	a = 7.0535(7) Å	α = 77.936(3)°
	b = 7.1264(7) Å	β = 75.463(4)°
	c = 9.2329(9) Å	γ = 80.201(4)°
**Volume**	435.91(7) $Å^3$	
**Z**	2	
**Density (calculated)**	1.563 g/cm^3	
**Absorption coefficient**	0.121 mm^{-1}	
**F(000)**	212	

Table S3. Data collection and structure refinement for 3a.

**Theta range for data collection**	2.31 to 28.35°	
**Index ranges**	$-9 \leq h \leq 9$, $-9 \leq k \leq 9$, $-12 \leq l \leq 12$	
**Reflections collected**	15866	
**Independent reflections**	2190 [R(int) = 0.0673]	
**Coverage of independent reflections**	99.9%	
**Absorption correction**	Multi-Scan	
**Max. and min. transmission**	0.9980 and 0.9880	
**Structure solution technique**	direct methods	
**Structure solution program**	SHELXT 2018/2 (Sheldrick, 2018)	
**Refinement method**	Full-matrix least-squares on F^2	
**Refinement program**	SHELXL-2018/3 (Sheldrick, 2018)	
**Function minimized**	$\Sigma w(F_o^2 - F_c^2)^2$	
**Data / restraints / parameters**	2190 / 0 / 137	
**Goodness-of-fit on F^2**	1.038	
**Final R indices**	1364 data; $I>2\sigma(I)$	R_1 = 0.0710, wR_2 = 0.1627
	all data	R_1 = 0.1197, wR_2 = 0.1872
**Weighting scheme**	$w=1/[\sigma^2(F_o^2)+(0.0808P)^2+0.1839P]$ where $P=(F_o^2+2F_c^2)/3$	
**Largest diff. peak and hole**	0.261 and -0.321 eÅ^{-3}	
**R.M.S. deviation from mean**	0.051 eÅ^{-3}	

Table S4. Atomic coordinates and equivalent isotropic atomic displacement parameters (Å^2) for 3a.

U(eq) is defined as one third of the trace of the orthogonalized U_{ij} tensor

	x/a	y/b	z/c	U(eq)
C1	0.7037(3)	0.1764(4)	0.1130(3)	0.0379(6)
C2	0.8772(4)	0.1204(4)	0.1799(3)	0.0437(6)
C3	0.7057(3)	0.1802(3)	0.4130(3)	0.0301(5)
C4	0.5301(3)	0.2413(3)	0.3609(2)	0.0284(5)
C5	0.3573(3)	0.3004(3)	0.4624(2)	0.0315(5)
C6	0.3662(3)	0.2968(3)	0.6096(2)	0.0327(5)
C7	0.5365(4)	0.2365(3)	0.6644(3)	0.0353(6)
C8	0.7053(4)	0.1788(3)	0.5653(3)	0.0380(6)
C9	0.3529(4)	0.2963(5)	0.1522(3)	0.0499(7)
N1	0.5332(3)	0.2392(3)	0.2102(2)	0.0332(5)
N2	0.8813(3)	0.1203(3)	0.3174(2)	0.0410(5)
N3	0.1830(3)	0.3633(3)	0.7132(2)	0.0407(5)
O1	0.7104(3)	0.1654(3)	0.9814(2)	0.0579(6)
O2	0.0320(3)	0.4068(3)	0.6670(2)	0.0606(6)
O3	0.1904(3)	0.3749(4)	0.8408(2)	0.0763(8)

Table S5. Bond lengths (Å) for 3a.

C1-O1	1.222(3)	C1-N1	1.376(3)
C1-C2	1.466(3)	C2-N2	1.276(3)
C2-H2	0.93	C3-N2	1.387(3)
C3-C8	1.403(3)	C3-C4	1.408(3)
C4-N1	1.389(3)	C4-C5	1.399(3)
C5-C6	1.371(3)	C5-H5	0.93
C6-C7	1.386(3)	C6-N3	1.473(3)
C7-C8	1.367(3)	C7-H7	0.93
C8-H8	0.93	C9-N1	1.464(3)
C9-H9A	0.96	C9-H9B	0.96
C9-H9C	0.96	N3-O3	1.213(3)
N3-O2	1.214(3)		

Table S6. Bond angles (°) for 3a.

O1-C1-N1	122.9(2)	O1-C1-C2	122.2(2)
N1-C1-C2	114.9(2)	N2-C2-C1	126.2(2)
N2-C2-H2	116.9	C1-C2-H2	116.9
N2-C3-C8	118.5(2)	N2-C3-C4	121.7(2)
C8-C3-C4	119.8(2)	N1-C4-C5	121.91(19)
N1-C4-C3	118.88(19)	C5-C4-C3	119.2(2)
C6-C5-C4	118.3(2)	C6-C5-H5	120.8
C4-C5-H5	120.8	C5-C6-C7	123.8(2)
C5-C6-N3	117.1(2)	C7-C6-N3	119.1(2)
C8-C7-C6	117.9(2)	C8-C7-H7	121.0
C6-C7-H7	121.0	C7-C8-C3	120.9(2)
C7-C8-H8	119.5	C3-C8-H8	119.5
N1-C9-H9A	109.5	N1-C9-H9B	109.5
H9A-C9-H9B	109.5	N1-C9-H9C	109.5
H9A-C9-H9C	109.5	H9B-C9-H9C	109.5
C1-N1-C4	121.01(19)	C1-N1-C9	118.19(19)
C4-N1-C9	120.76(18)	C2-N2-C3	117.3(2)
O3-N3-O2	122.8(2)	O3-N3-C6	118.4(2)
O2-N3-C6	118.85(19)		

Table S7. Anisotropic atomic displacement parameters (Å^2) for 3a.

The anisotropic atomic displacement factor exponent takes the form: $-2\pi^2[h^2 a^{*2} U_{11} + ... + 2 h k a^* b^* U_{12}]$

	U_{11}	U_{22}	U_{33}	U_{23}	U_{13}	U_{12}
C1	0.0299(12)	0.0478(15)	0.0344(13)	-0.0110(11)	-0.0035(10)	-0.0011(11)
C2	0.0295(13)	0.0581(17)	0.0420(14)	-0.0175(12)	-0.0040(11)	0.0032(11)
C3	0.0264(12)	0.0302(12)	0.0335(12)	-0.0054(10)	-0.0087(9)	-0.0002(9)
C4	0.0285(12)	0.0267(11)	0.0299(12)	-0.0028(9)	-0.0092(9)	-0.0015(9)
C5	0.0265(11)	0.0358(13)	0.0310(12)	-0.0040(10)	-0.0097(9)	0.0020(9)
C6	0.0325(12)	0.0339(12)	0.0299(12)	-0.0063(10)	-0.0056(10)	-0.0004(10)
C7	0.0442(14)	0.0373(13)	0.0266(12)	-0.0059(10)	-0.0147(10)	0.0000(11)
C8	0.0365(13)	0.0414(14)	0.0399(13)	-0.0074(11)	-0.0198(11)	0.0017(11)
C9	0.0318(13)	0.086(2)	0.0324(13)	-0.0137(14)	-0.0142(11)	0.0053(13)
N1	0.0273(10)	0.0440(12)	0.0279(10)	-0.0063(8)	-0.0084(8)	0.0000(8)
N2	0.0287(11)	0.0516(13)	0.0423(12)	-0.0134(10)	-0.0093(9)	0.0047(9)
N3	0.0415(12)	0.0472(13)	0.0316(11)	-0.0091(9)	-0.0071(9)	0.0006(10)
O1	0.0466(12)	0.0922(16)	0.0345(10)	-0.0231(10)	-0.0068(8)	0.0042(10)
O2	0.0386(11)	0.0926(16)	0.0461(11)	-0.0224(11)	-0.0083(9)	0.0156(10)
O3	0.0655(14)	0.129(2)	0.0333(11)	-0.0333(12)	-0.0107(10)	0.0128(13)

Table S8. Hydrogen atomic coordinates and isotropic atomic displacement parameters (Å^2) for 3a.

	x/a	y/b	z/c	U(eq)
H2	0.9956	0.0811	0.1162	0.052
H5	0.2394	0.3411	0.4309	0.038
H7	0.5359	0.2353	0.7654	0.042
H8	0.8216	0.1379	0.5992	0.046
H9A	0.3792	0.2742	0.0495	0.075
H9B	0.3094	0.4310	0.1545	0.075
H9C	0.2518	0.2211	0.2145	0.075

^{1}H and ^{13}C NMR spectra of all compounds

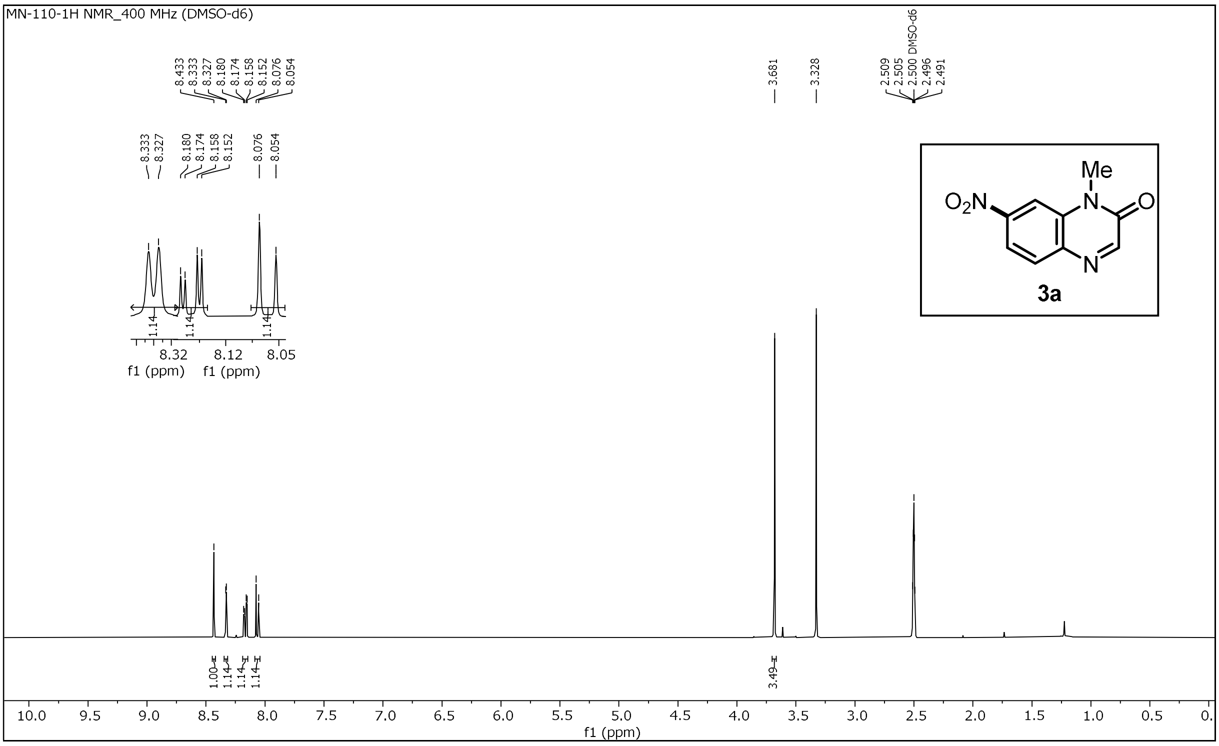

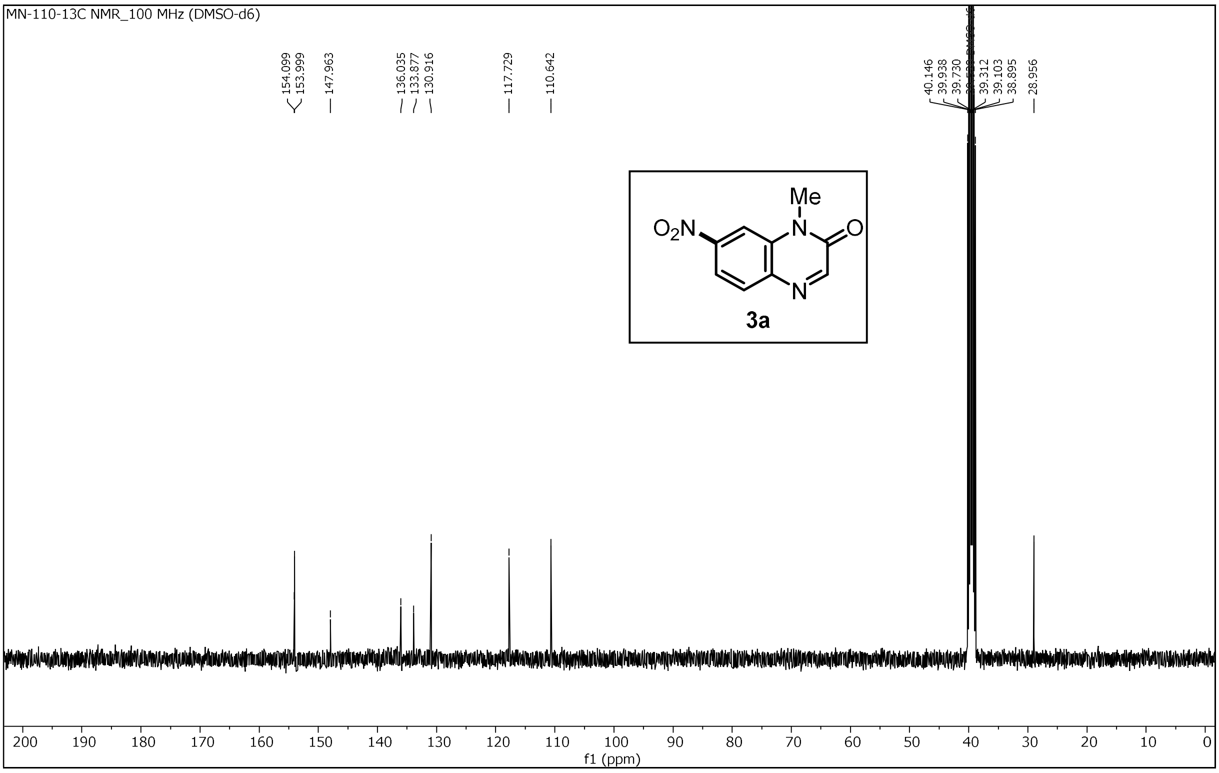

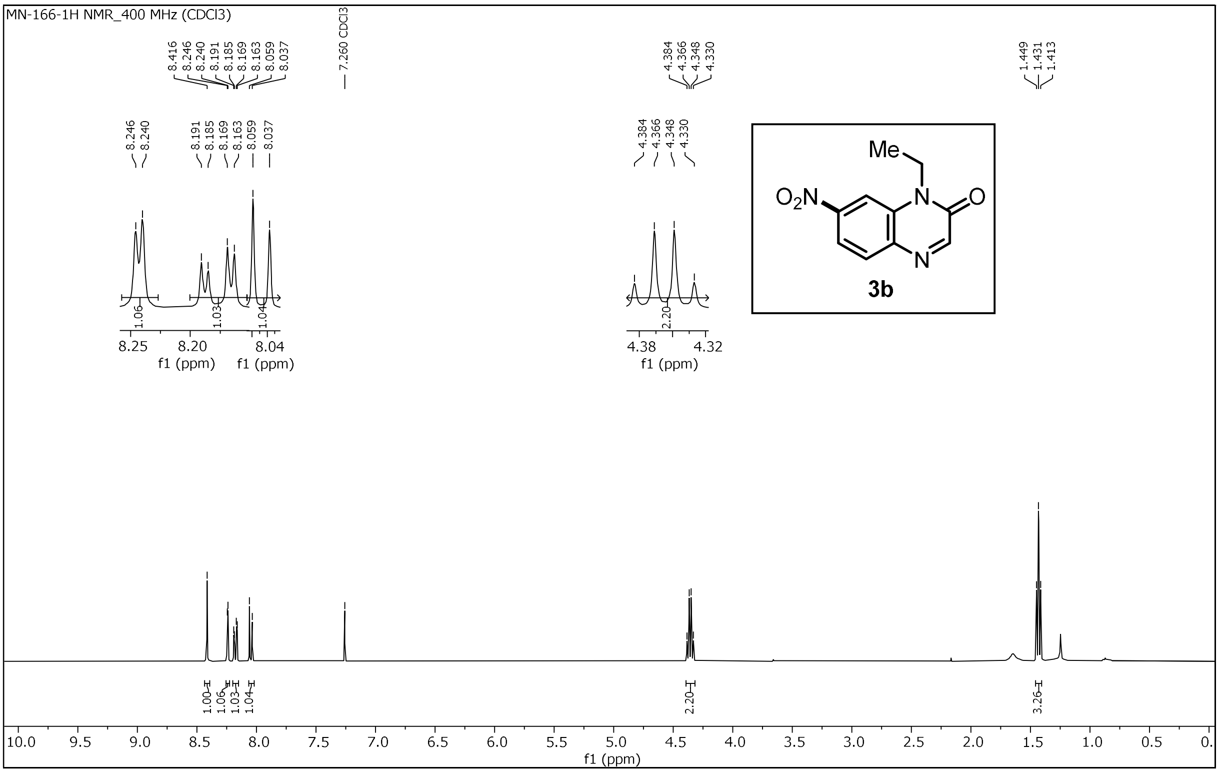
MN-166-1H NMR_400 MHz (CDCl3)
3b
f1 (ppm)

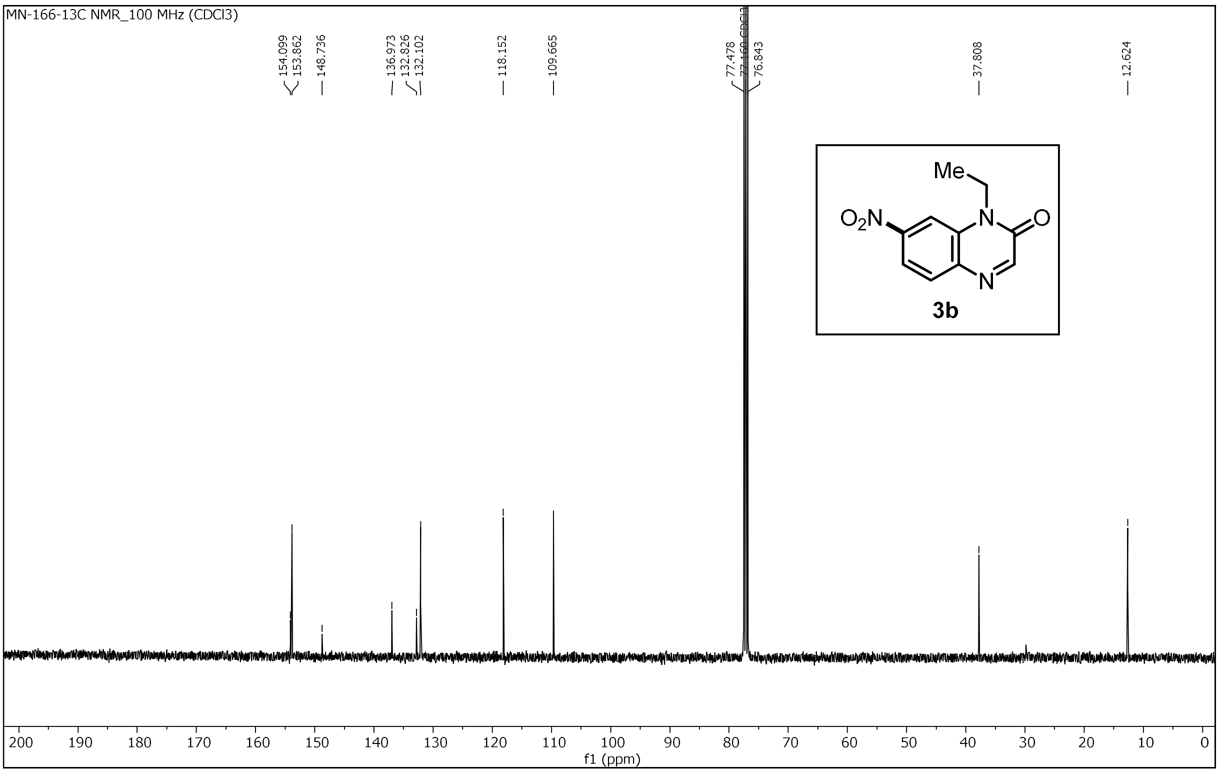
MN-166-13C NMR_100 MHz (CDCl3)
3b
f1 (ppm)

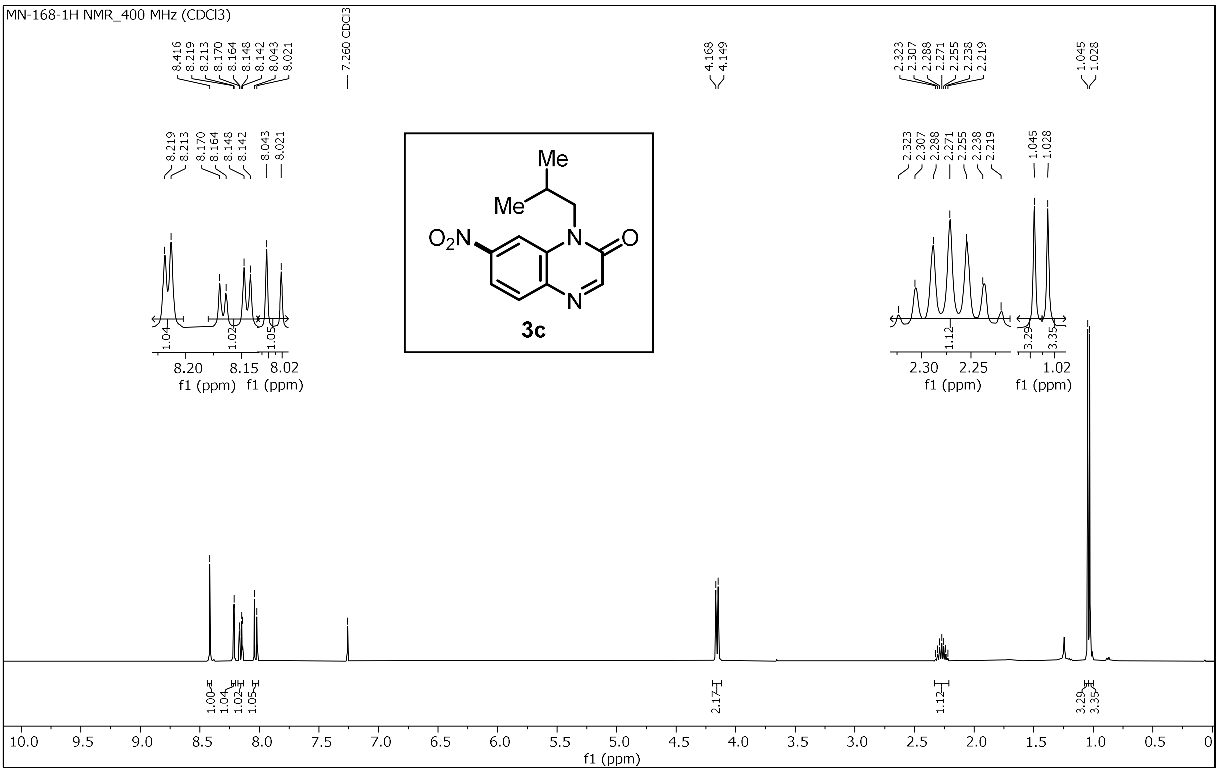
MN-168-1H NMR_400 MHz (CDCl3)
7.260 CDCl3
3c
f1 (ppm)

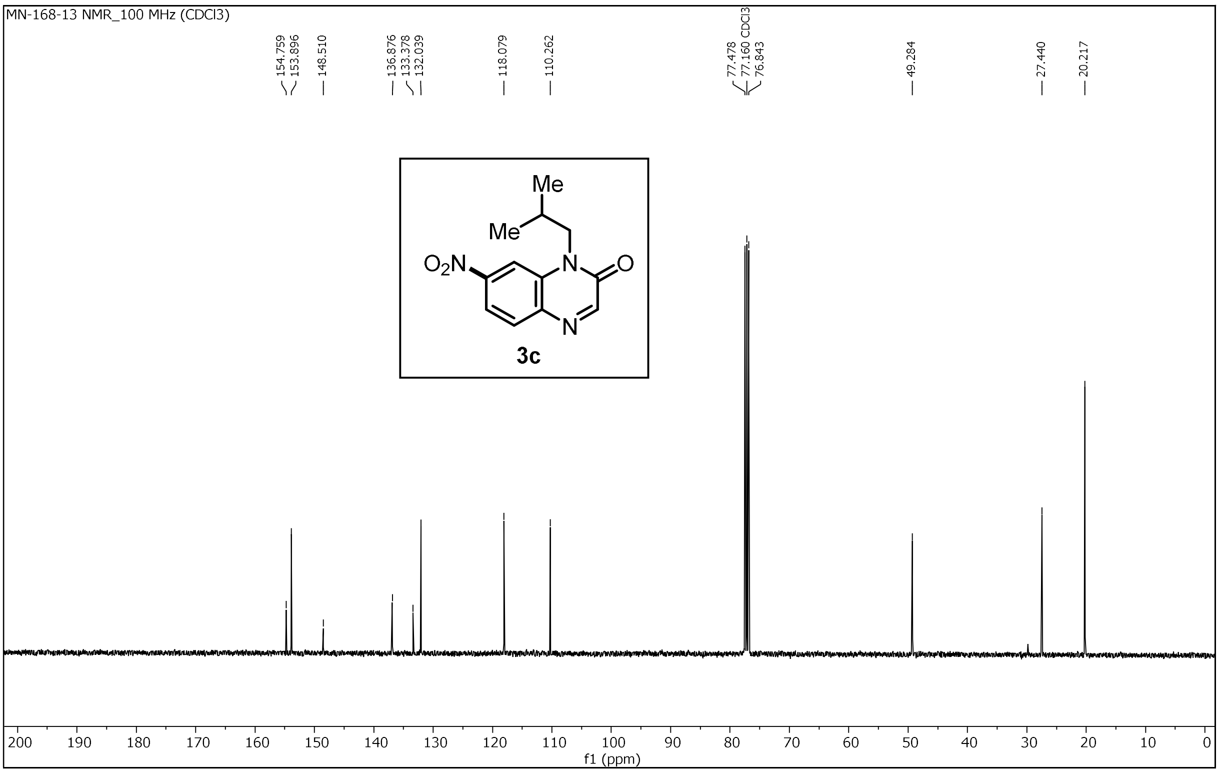
MN-168-13 NMR_100 MHz (CDCl3)
154.759
153.896
148.510
136.876
133.378
132.039
118.079
110.262
77.478
77.160 CDCl3
76.843
49.284
27.440
20.217
3c
f1 (ppm)

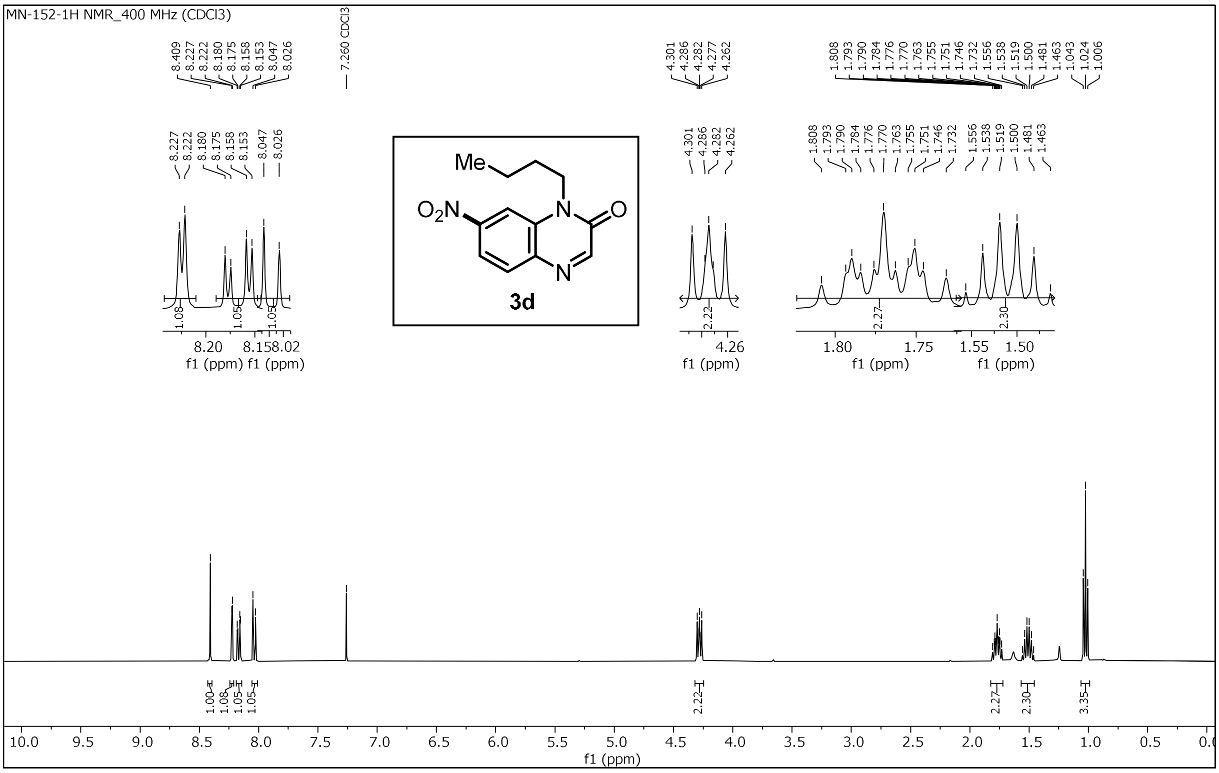
MN-152-1H NMR_400 MHz (CDCl3)
7.260 CDCl3
3d
f1 (ppm)

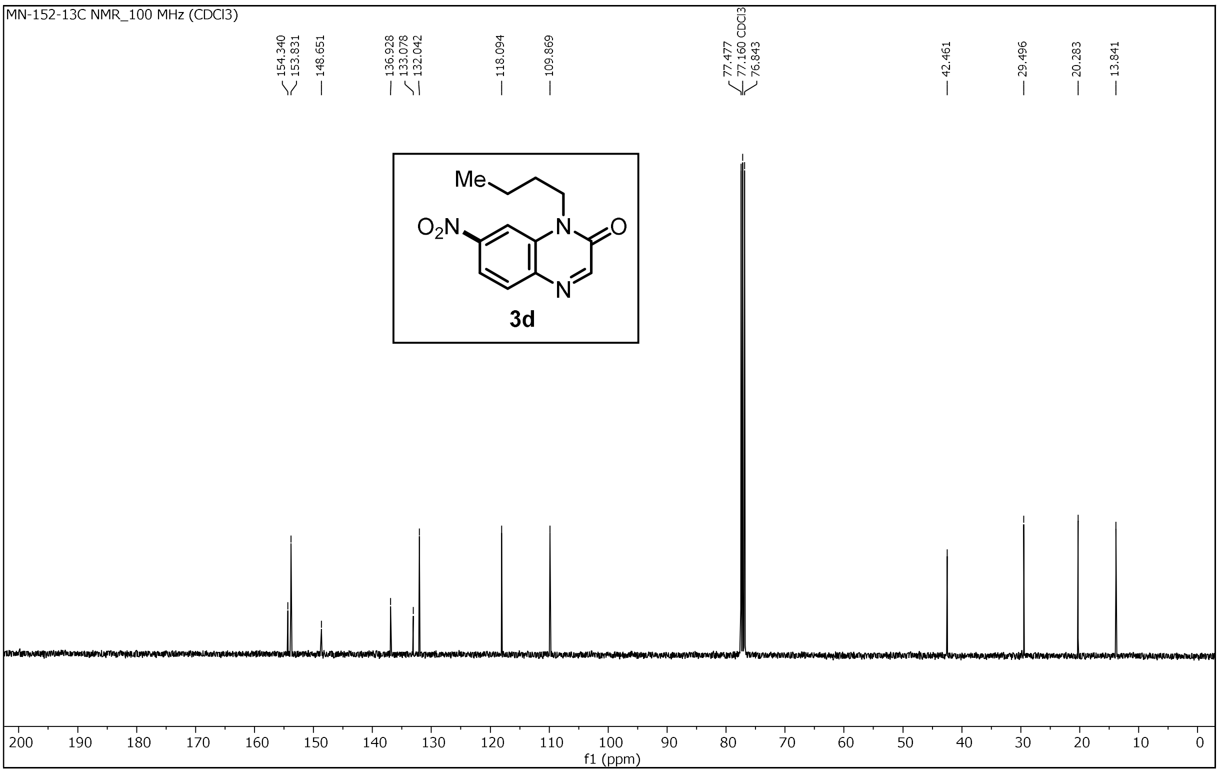
MN-152-13C NMR_100 MHz (CDCl3)
154.340
153.831
148.651
136.928
133.078
132.042
118.094
109.869
77.477
77.160 CDCl3
76.843
42.461
29.496
20.283
13.841
3d
f1 (ppm)

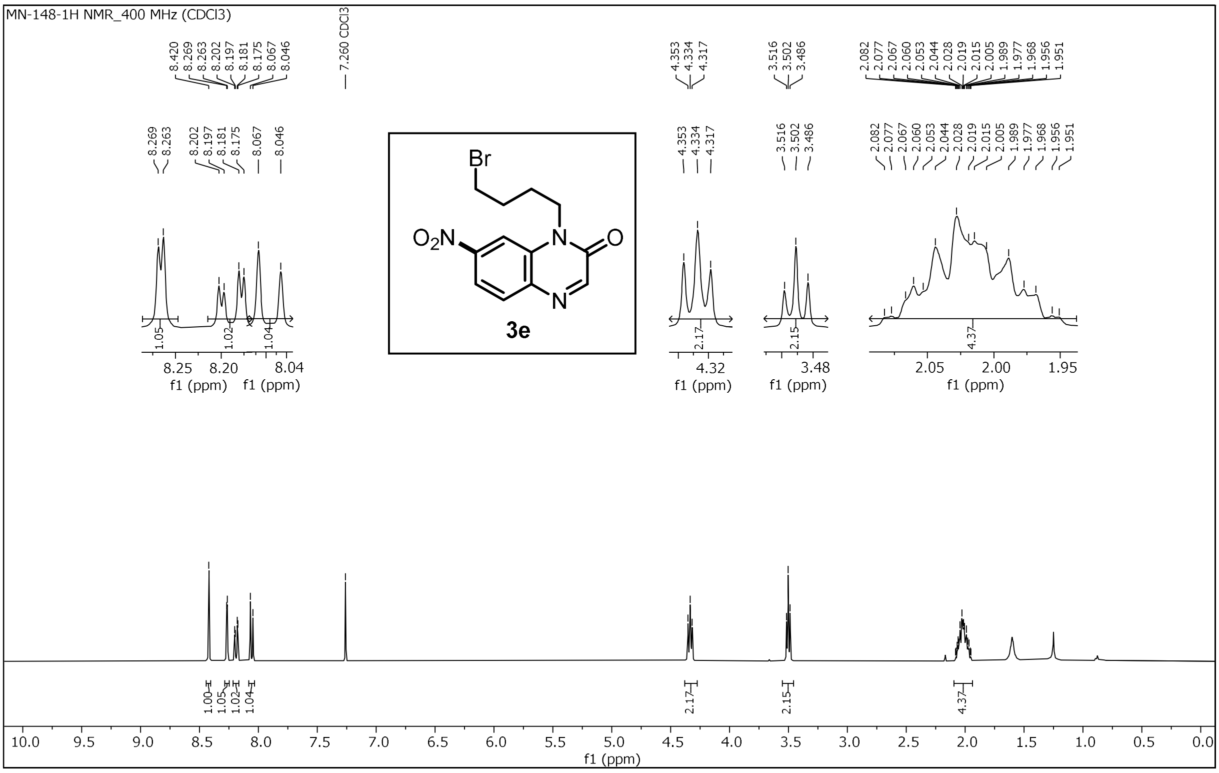
MN-148-1H NMR_400 MHz (CDCl3)
7.260 CDCl3
3e
f1 (ppm)

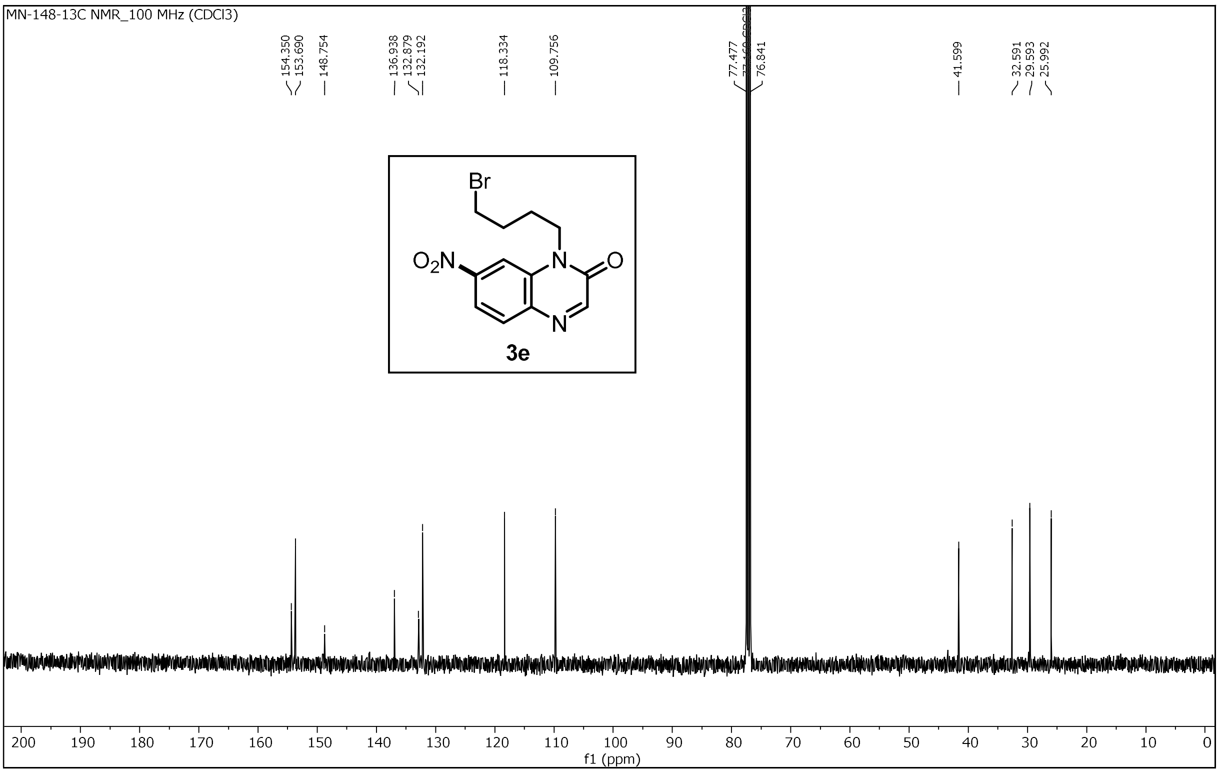
MN-148-13C NMR_100 MHz (CDCl3)
154.350
153.690
148.754
136.938
132.879
132.192
118.334
109.756
77.477
76.841
41.599
32.591
29.593
25.992
3e
f1 (ppm)

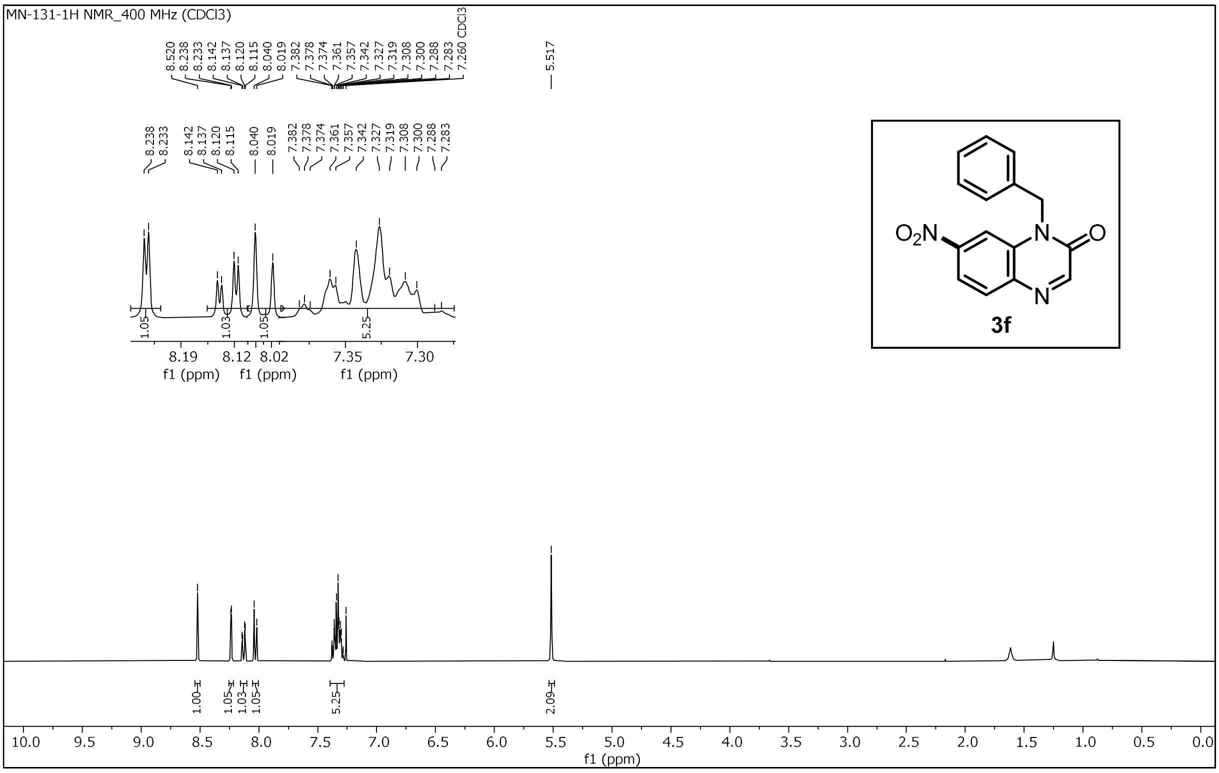
MN-131-1H NMR_400 MHz (CDCl3)
8.520 8.238 8.233 8.142 8.137 8.120 8.115 8.040 8.019 7.382 7.378 7.374 7.361 7.357 7.342 7.327 7.319 7.308 7.300 7.288 7.283 7.260 CDCl3
5.517
1.00 1.05 1.03 1.05 5.25 2.09
f1 (ppm)
O2N
N
O
N
3f

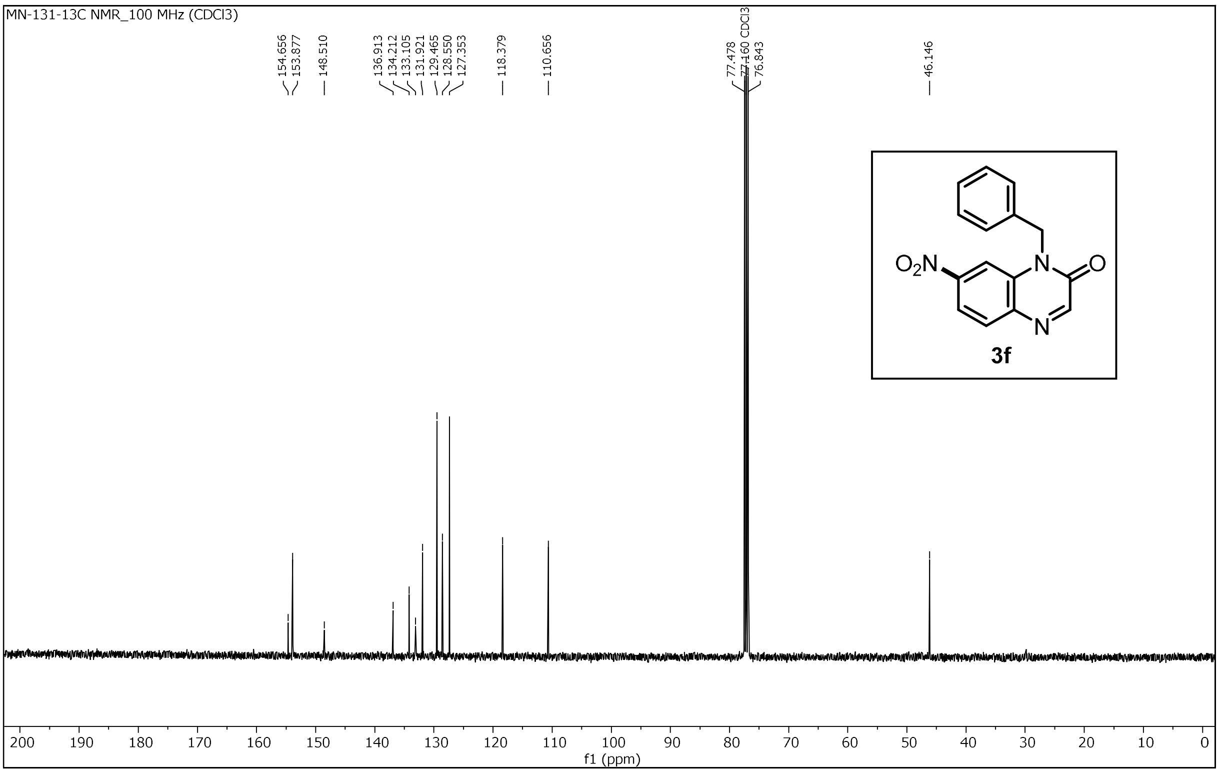
MN-131-13C NMR_100 MHz (CDCl3)
154.656 153.877 148.510 136.913 134.212 133.105 131.921 129.465 128.550 127.353 118.379 110.656
77.478 77.160 CDCl3 76.843
46.146
f1 (ppm)
O2N
N
O
N
3f

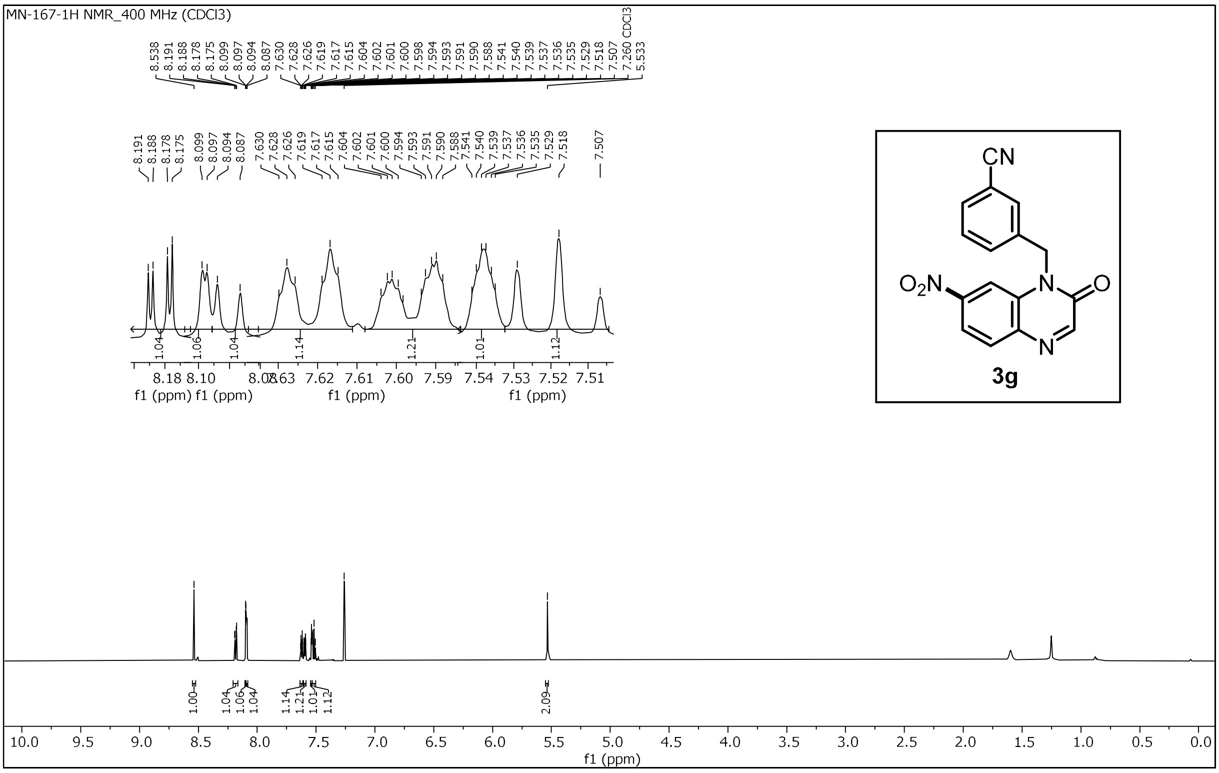
MN-167-1H NMR_400 MHz (CDCl3)
8.538
8.191
8.188
8.178
8.175
8.099
8.097
8.094
8.087
7.630
7.628
7.626
7.619
7.617
7.615
7.604
7.602
7.601
7.600
7.598
7.594
7.593
7.591
7.590
7.588
7.541
7.540
7.539
7.537
7.536
7.535
7.529
7.518
7.507
7.260 CDCl3
5.533
1.00
1.04
1.06
1.04
1.14
1.21
1.01
1.12
2.09
f1 (ppm)
CN
O_2N
N
O
N
3g

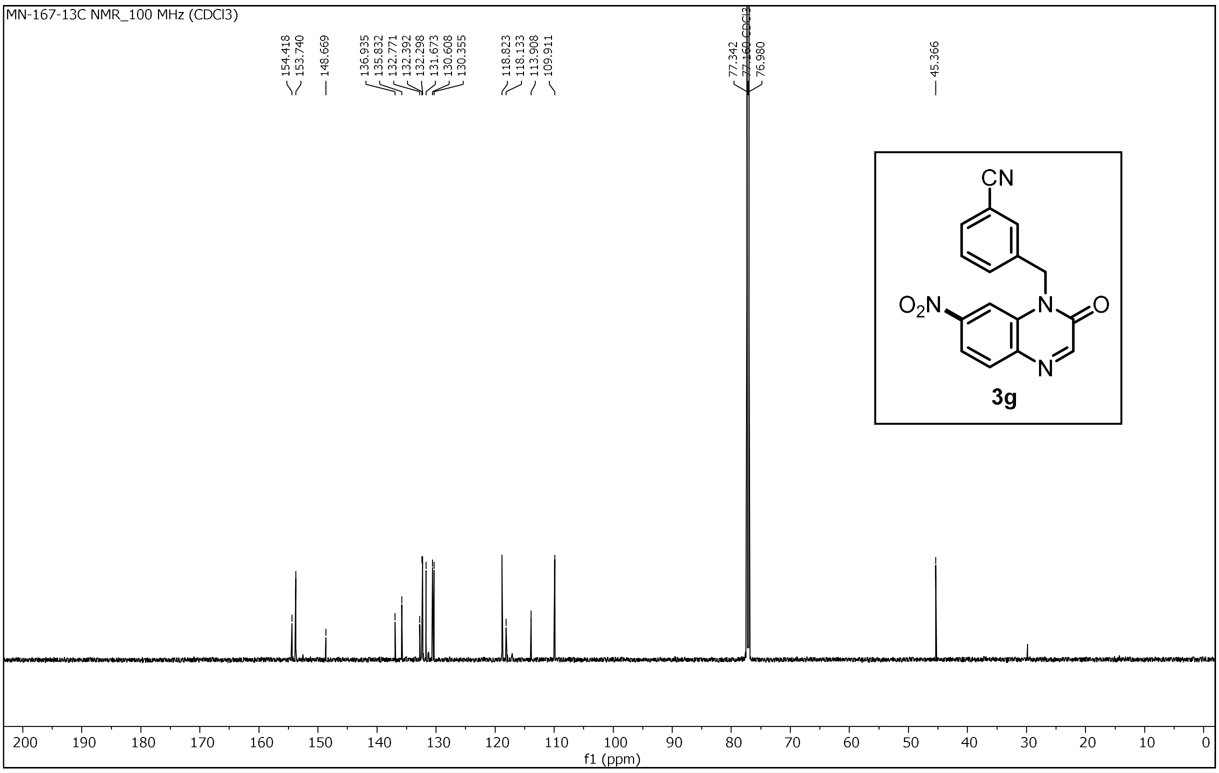
MN-167-13C NMR_100 MHz (CDCl3)
154.418
153.740
148.669
136.935
135.832
132.771
132.392
132.298
131.673
130.608
130.355
118.823
118.133
113.908
109.911
77.342
77.160 CDCl3
76.980
45.366
f1 (ppm)
CN
O_2N
N
O
N
3g

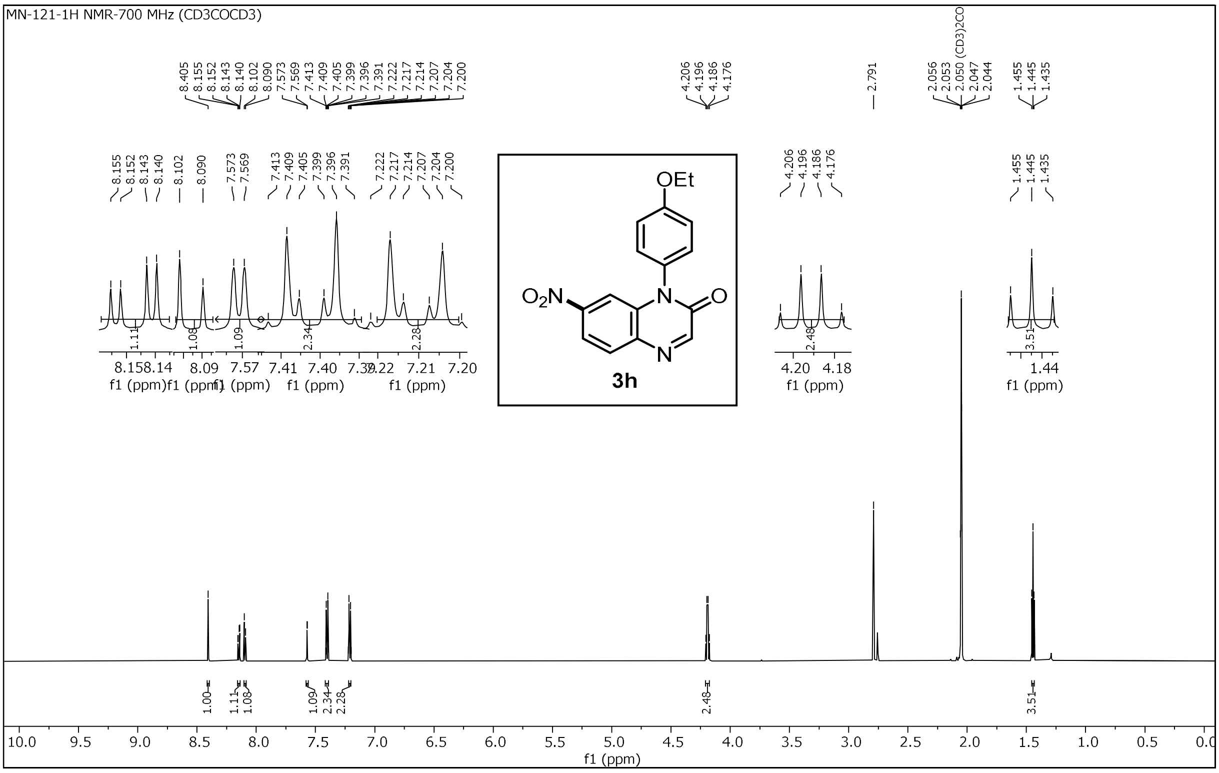
MN-121-1H NMR-700 MHz (CD3COCD3)
OEt
O2N
N
O
N
3h
f1 (ppm)

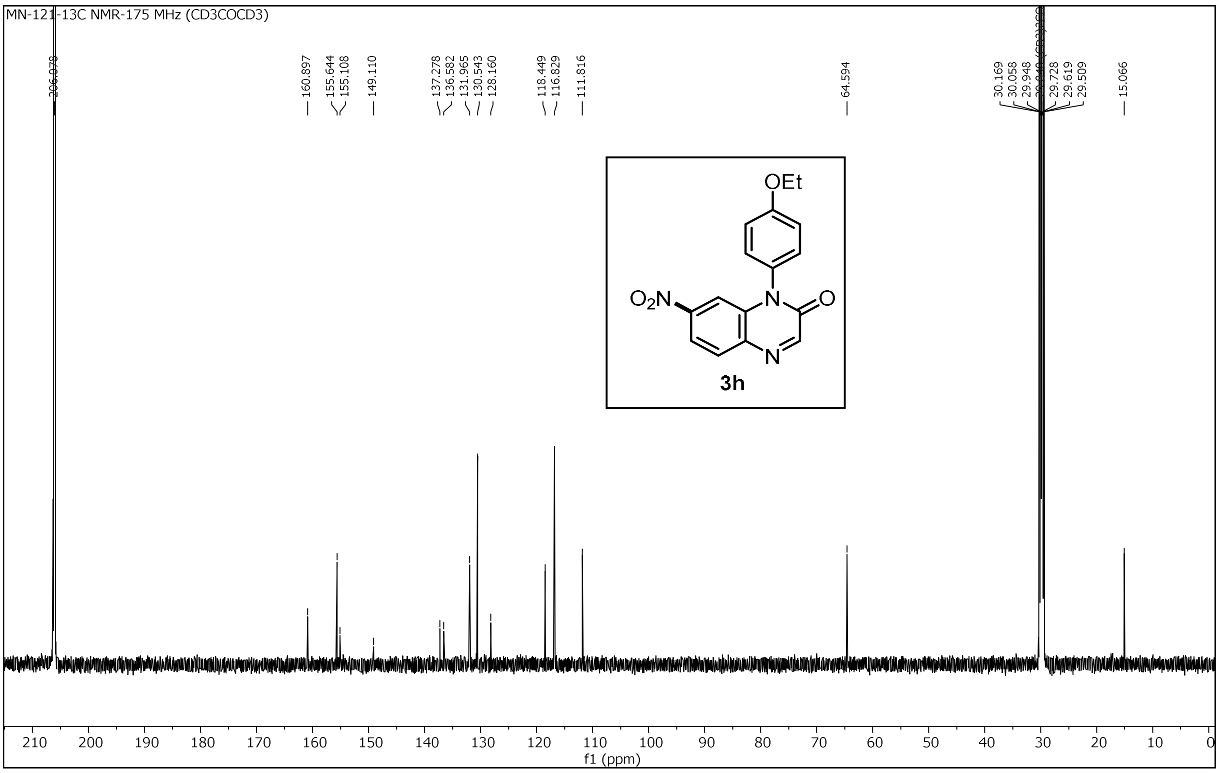
MN-121-13C NMR-175 MHz (CD3COCD3)
OEt
O2N
N
O
N
3h
f1 (ppm)

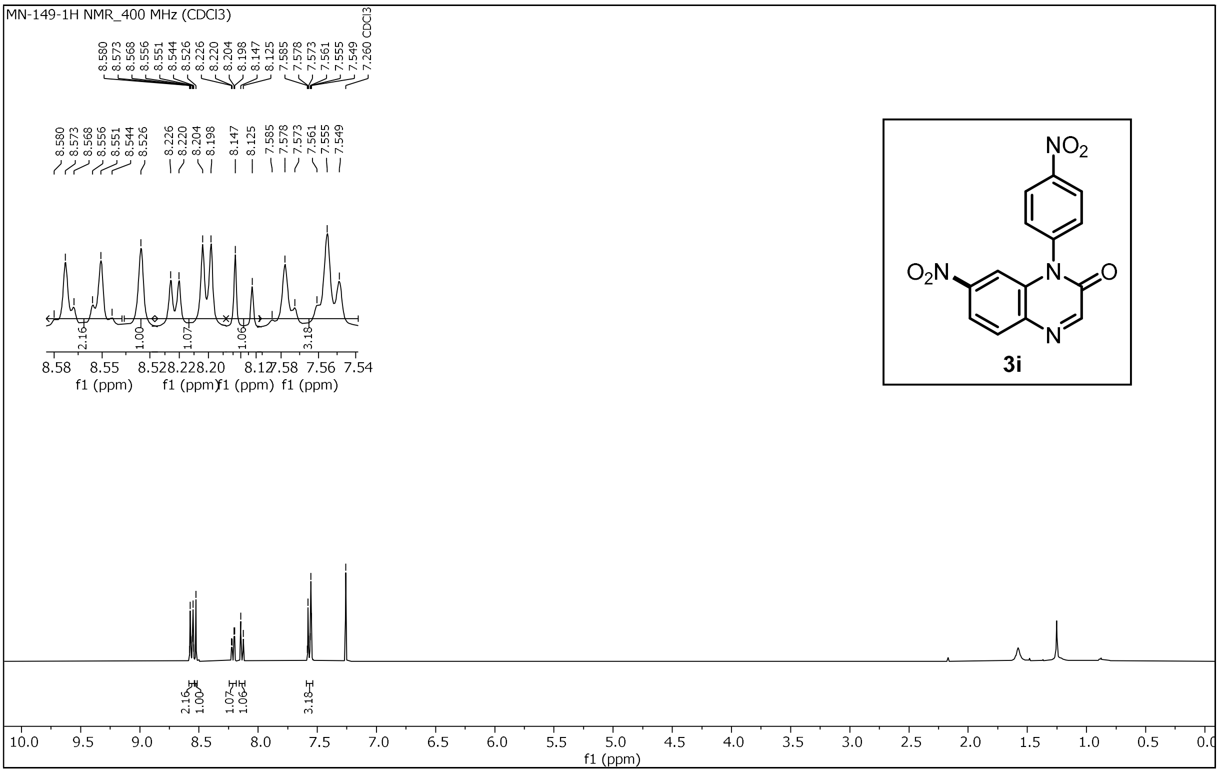
MN-149-1H NMR_400 MHz (CDCl3)
8.580 8.573 8.568 8.556 8.551 8.544 8.526 8.226 8.220 8.204 8.198 8.147 8.125 7.585 7.578 7.573 7.561 7.555 7.549 7.260 CDCl3
2.16 1.00 1.07 1.06 3.18
f1 (ppm)
NO2
O2N
N
O
N
3i

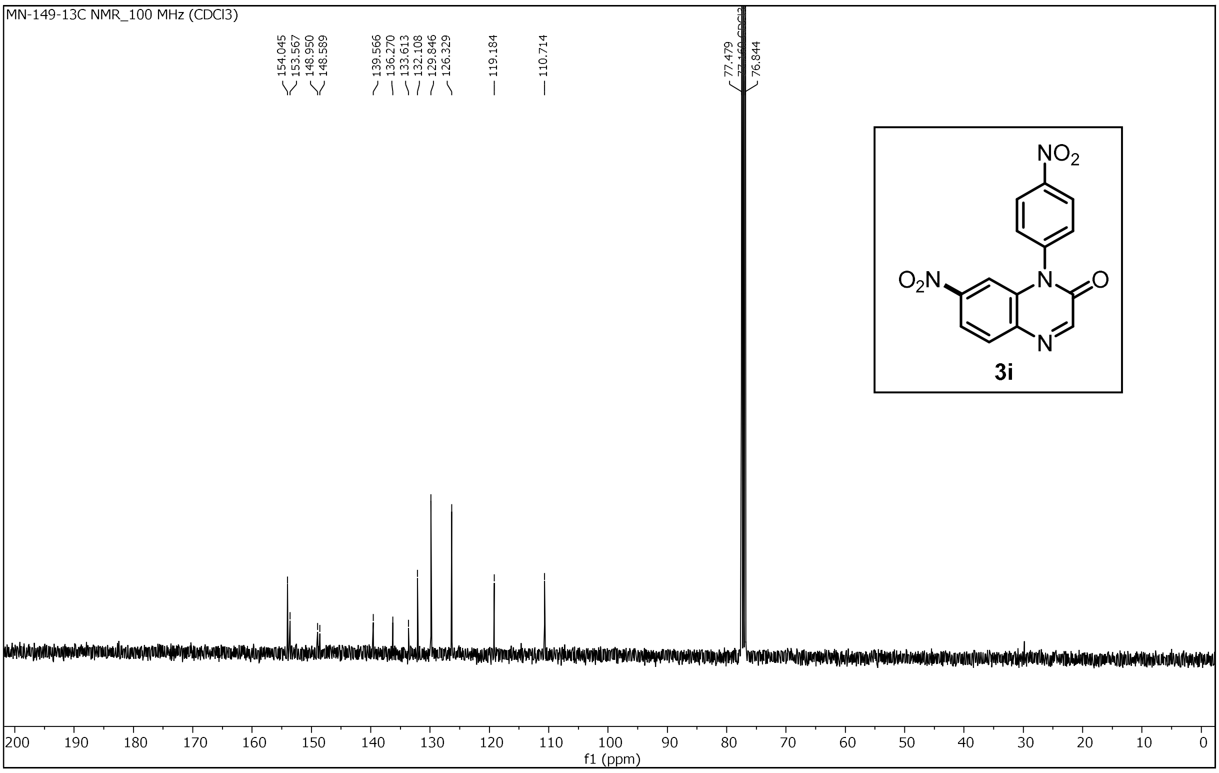
MN-149-13C NMR_100 MHz (CDCl3)
154.045 153.567 148.950 148.589 139.566 136.270 133.613 132.108 129.846 126.329 119.184 110.714 77.479 76.844
f1 (ppm)
NO2
O2N
N
O
N
3i

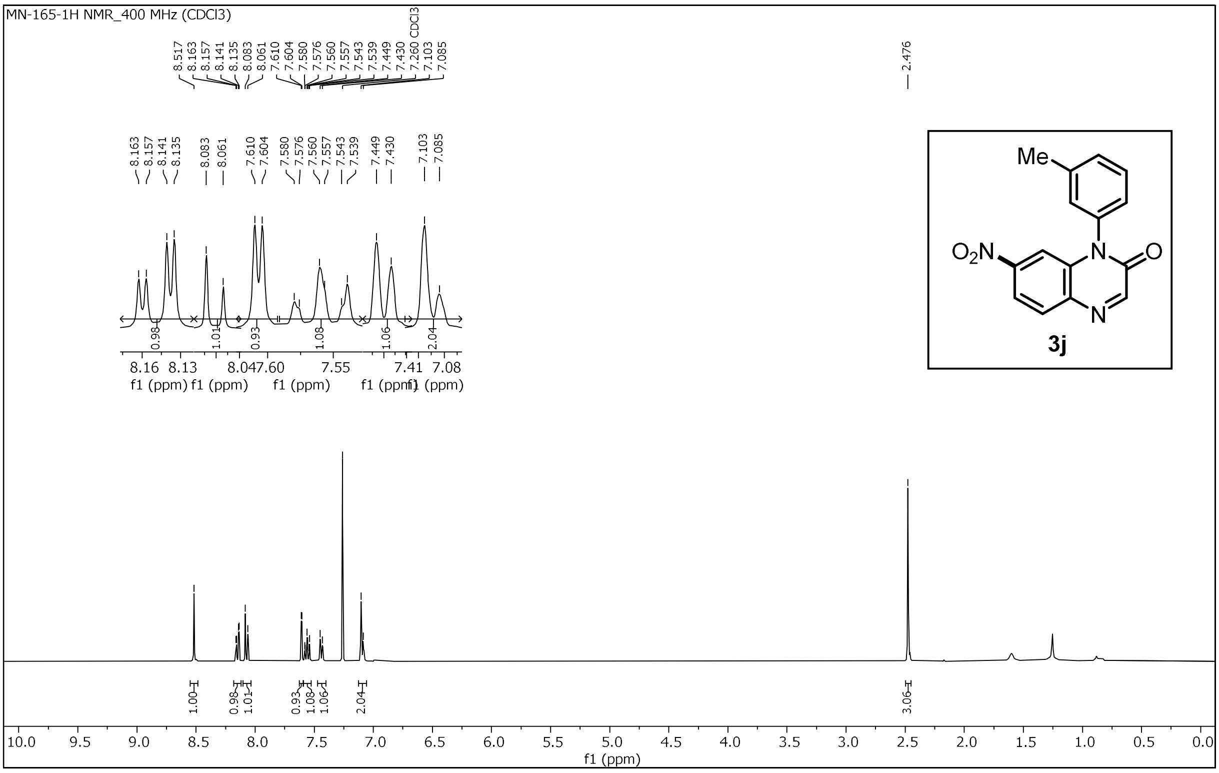
MN-165-1H NMR_400 MHz (CDCl3)
8.517
8.163
8.157
8.141
8.135
8.083
8.061
7.610
7.604
7.580
7.576
7.560
7.557
7.543
7.539
7.449
7.430
7.260 CDCl3
7.103
7.085
2.476
Me
O_2N
N
O
N
3j
1.00
0.98
1.01
0.93
1.08
1.06
2.04
3.06
f1 (ppm)

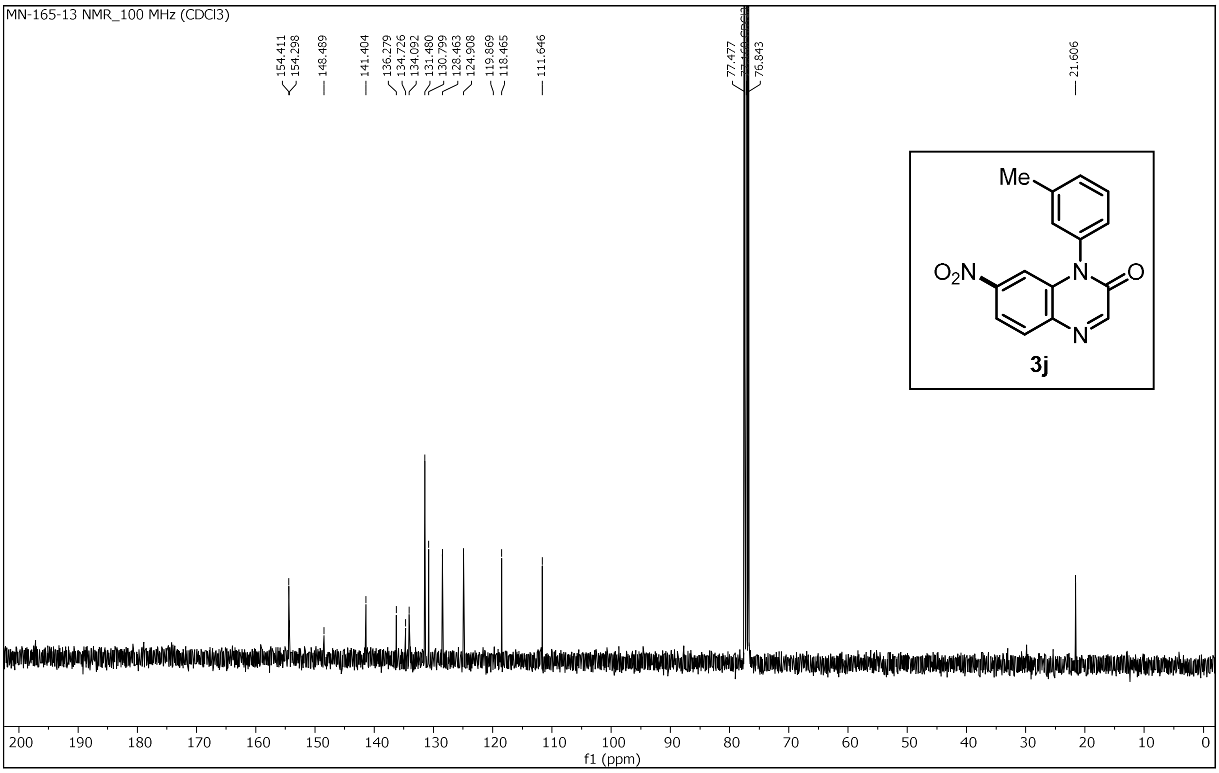
MN-165-13 NMR_100 MHz (CDCl3)
154.411
154.298
148.489
141.404
136.279
134.726
134.092
131.480
130.799
128.463
124.908
119.869
118.465
111.646
77.477
76.843
21.606
Me
O_2N
N
O
N
3j
f1 (ppm)

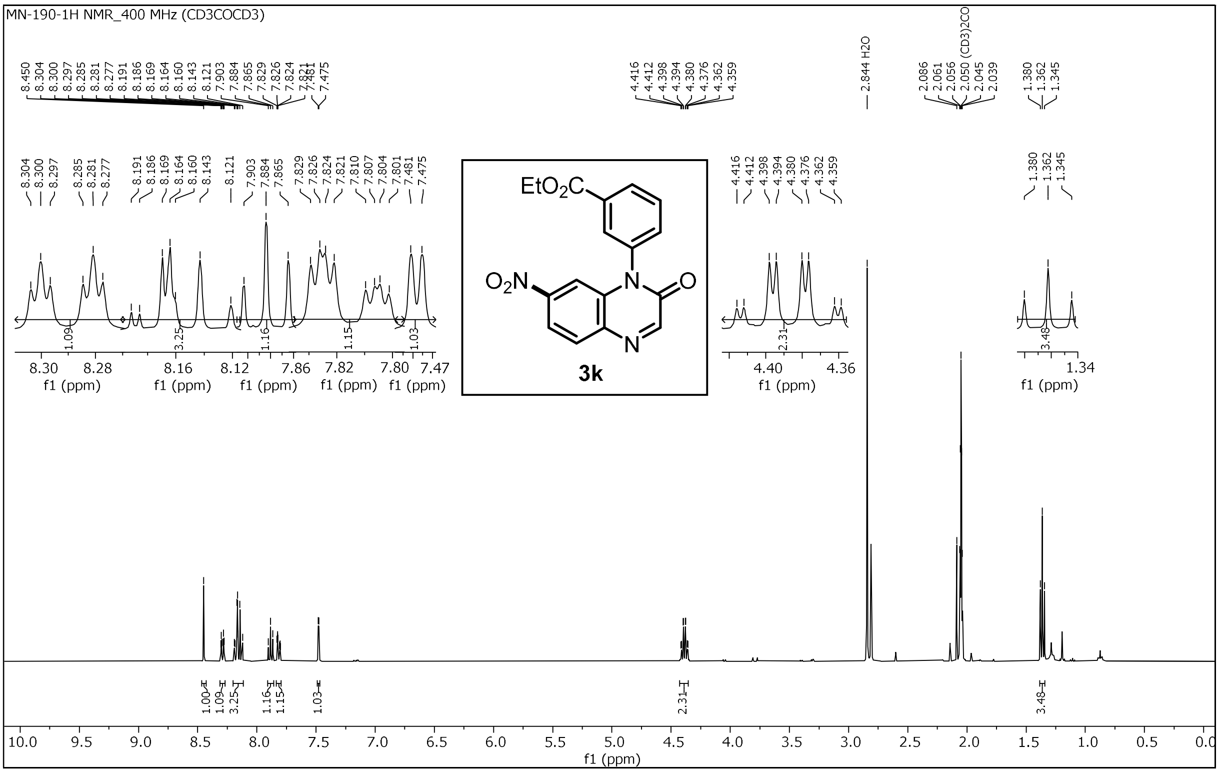
MN-190-1H NMR_400 MHz (CD3COCD3)
8.450 8.304 8.300 8.297 8.285 8.281 8.277 8.191 8.186 8.169 8.164 8.160 8.143 8.121 7.903 7.884 7.865 7.829 7.826 7.824 7.821 7.481 7.475
4.416 4.412 4.398 4.394 4.380 4.376 4.362 4.359
2.844 H2O
2.086 2.061 2.056 2.050 (CD3)2CO 2.045 2.039
1.380 1.362 1.345
EtO2C
O2N
N
O
3k
1.00 1.09 3.25 1.16 1.15 1.03 2.31 3.48
f1 (ppm)

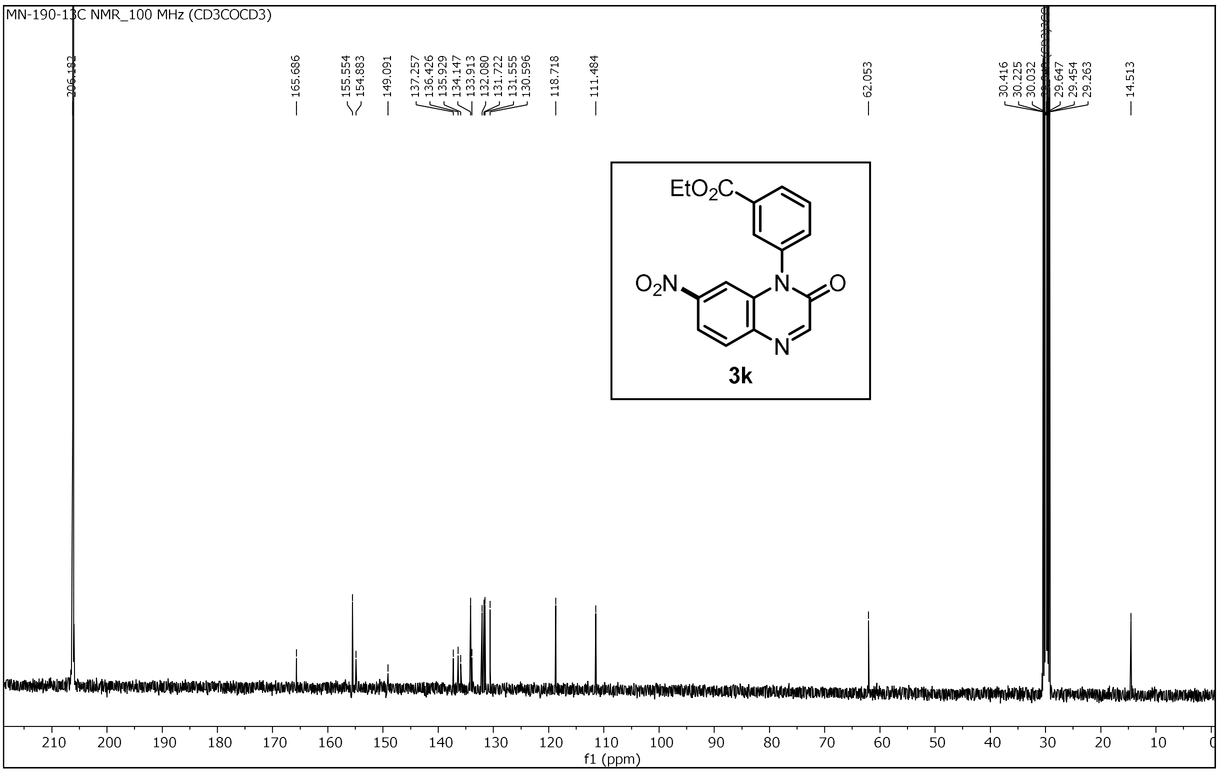
MN-190-13C NMR_100 MHz (CD3COCD3)
206.182
165.686
155.554 154.883
149.091
137.257 136.426 135.929 134.147 133.913 132.080 131.722 131.555 130.596
118.718
111.484
62.053
30.416 30.225 30.032 29.647 29.454 29.263
14.513
EtO2C
O2N
N
O
3k
f1 (ppm)

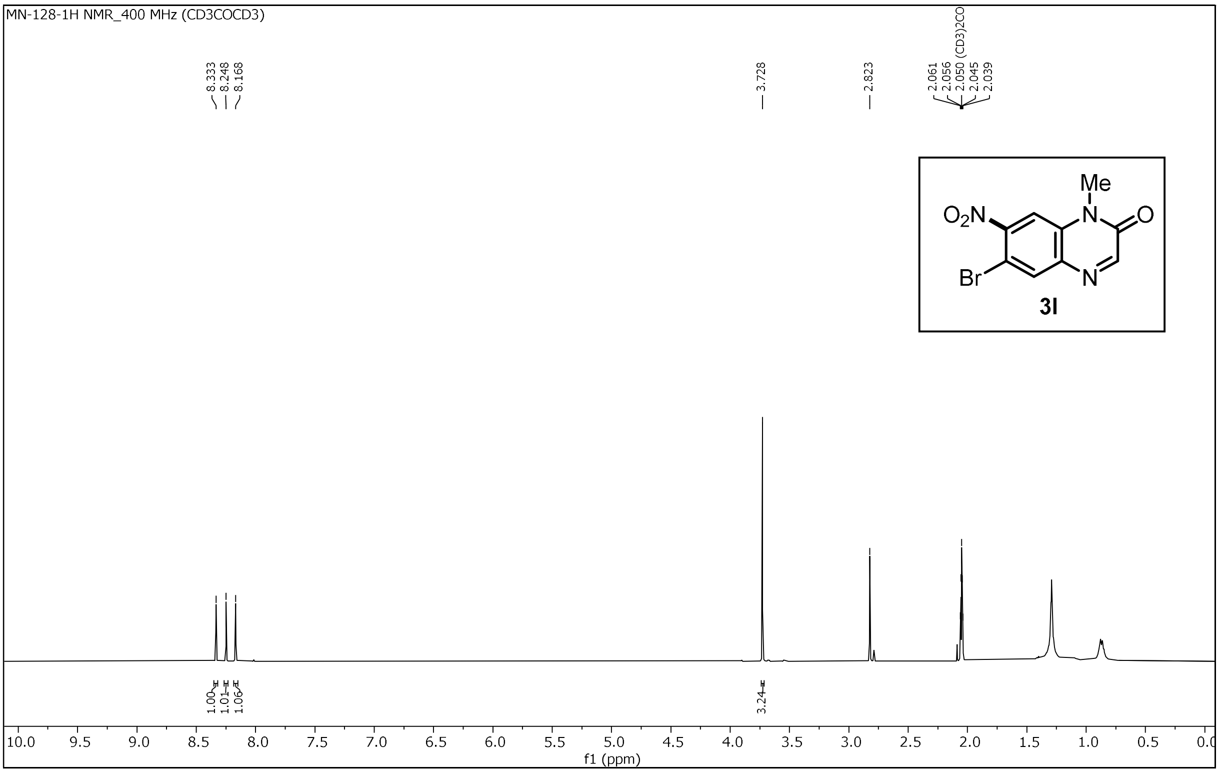
MN-128-1H NMR_400 MHz (CD3COCD3)
8.333
8.248
8.168
3.728
2.823
2.061
2.056
2.050 (CD3)2CO
2.045
2.039
Me
O2N
N
O
Br
N
3l
1.00
1.01
1.06
3.24
f1 (ppm)

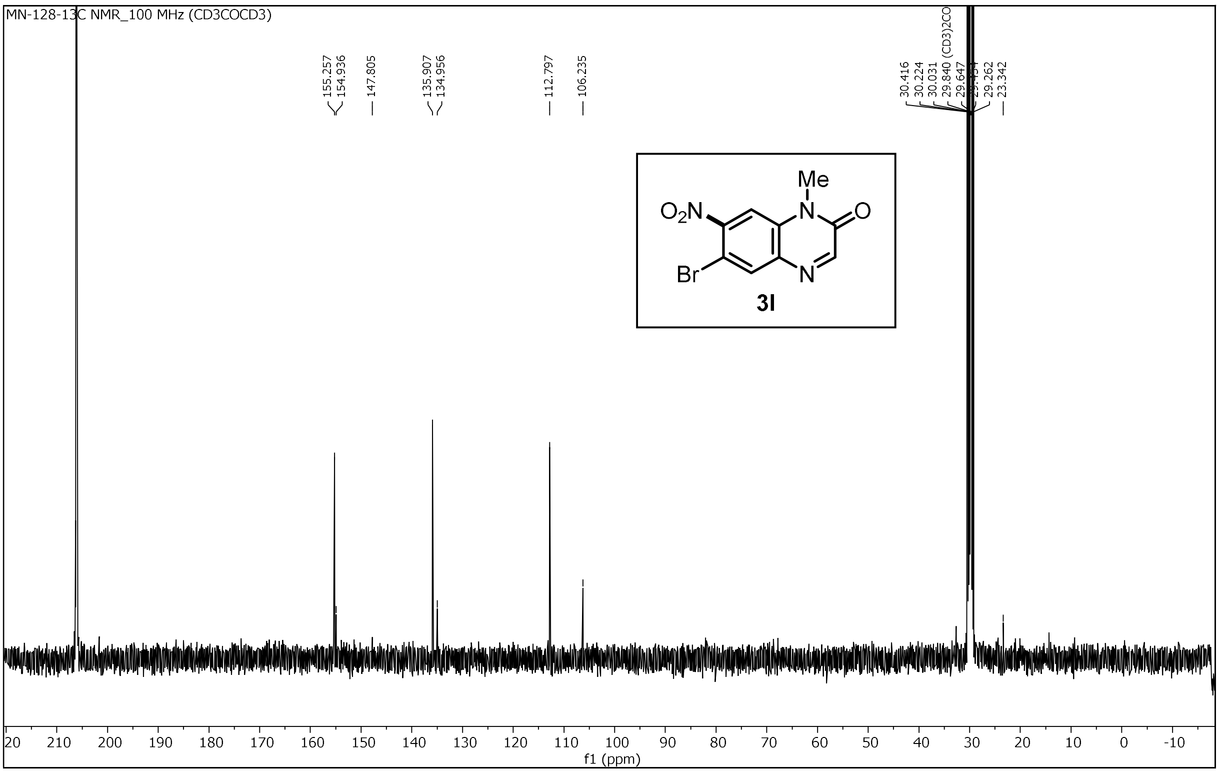
MN-128-13C NMR_100 MHz (CD3COCD3)
155.257
154.936
147.805
135.907
134.956
112.797
106.235
30.416
30.224
30.031
29.840 (CD3)2CO
29.647
29.454
29.262
23.342
Me
O2N
N
O
Br
N
3l
f1 (ppm)

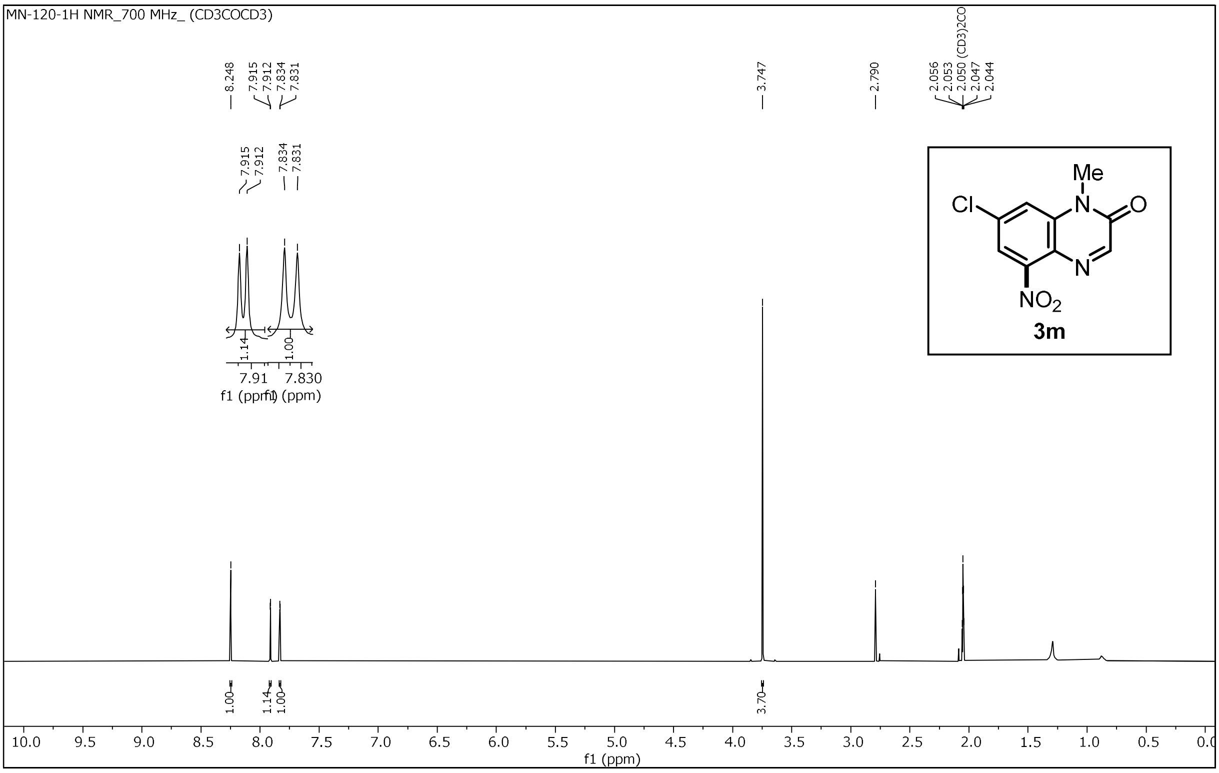
MN-120-1H NMR_700 MHz_ (CD3COCD3)
8.248
7.915
7.912
7.834
7.831
3.747
2.790
2.056
2.053
2.050 (CD3)2CO
2.047
2.044
1.14
1.00
7.91
7.830
f1 (ppm)
1.00
1.14
1.00
3.70
10.0 9.5 9.0 8.5 8.0 7.5 7.0 6.5 6.0 5.5 5.0 4.5 4.0 3.5 3.0 2.5 2.0 1.5 1.0 0.5 0.0
f1 (ppm)
Me
Cl
N
O
N
NO2
3m

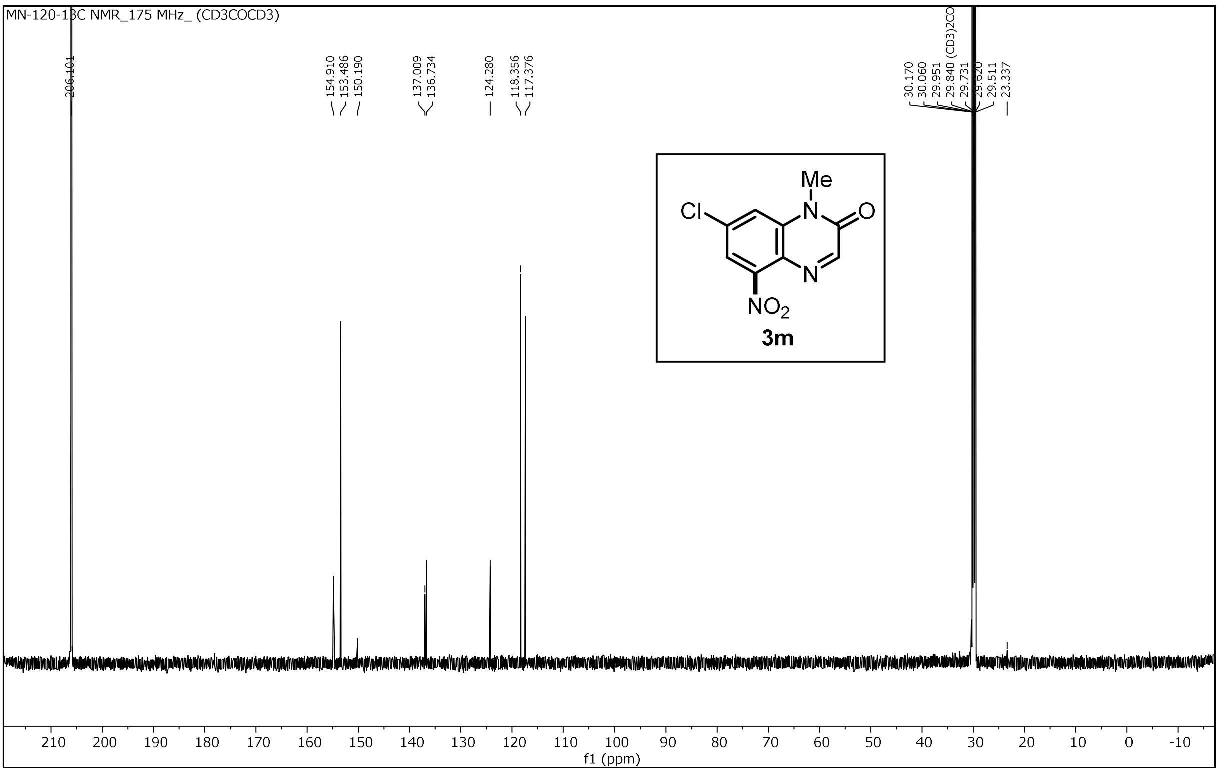
MN-120-13C NMR_175 MHz_ (CD3COCD3)
206.101
154.910
153.486
150.190
137.009
136.734
124.280
118.356
117.376
30.170
30.060
29.951
29.840 (CD3)2CO
29.731
29.620
29.511
23.337
210 200 190 180 170 160 150 140 130 120 110 100 90 80 70 60 50 40 30 20 10 0 -10
f1 (ppm)
Me
Cl
N
O
N
NO2
3m

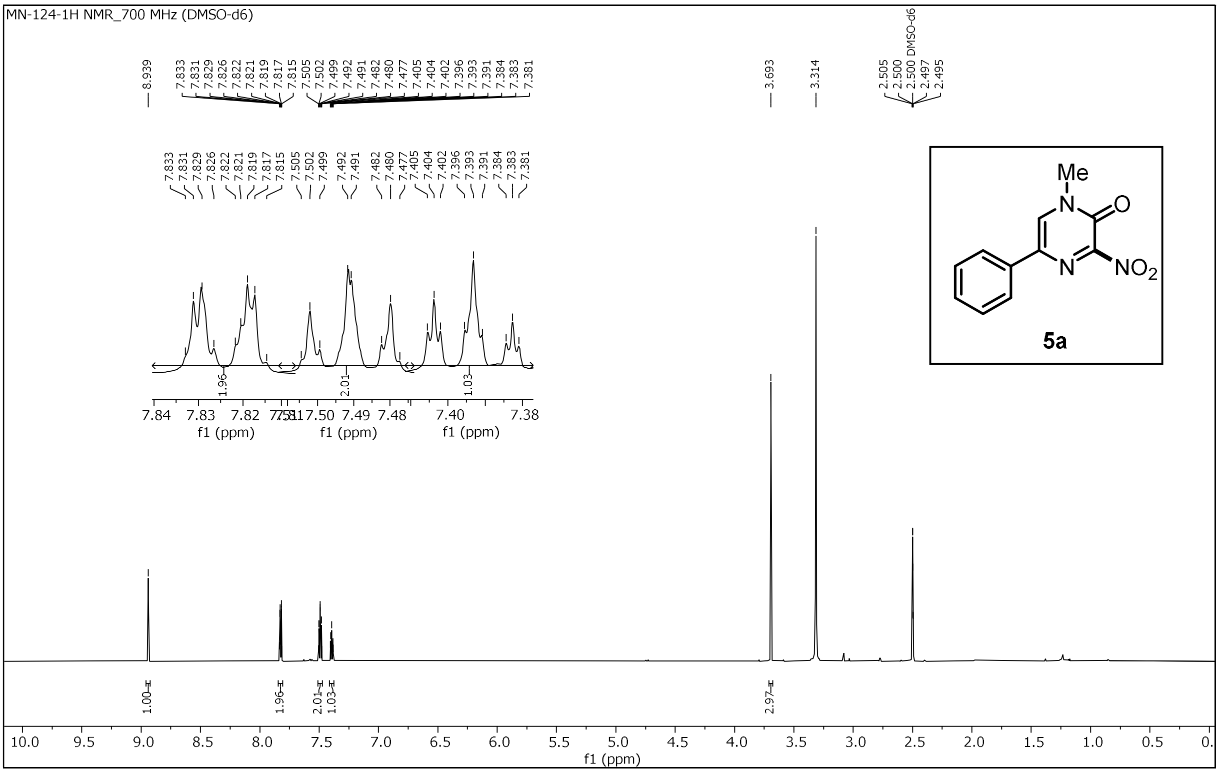
MN-124-1H NMR_700 MHz (DMSO-d6)
8.939
7.833 7.831 7.829 7.826 7.822 7.821 7.819 7.817 7.815 7.505 7.502 7.499 7.492 7.491 7.482 7.480 7.477 7.405 7.404 7.402 7.396 7.393 7.391 7.384 7.383 7.381
3.693
3.314
2.505 2.500 2.500 DMSO-d6 2.497 2.495
Me
N
O
N
NO2
5a
f1 (ppm)

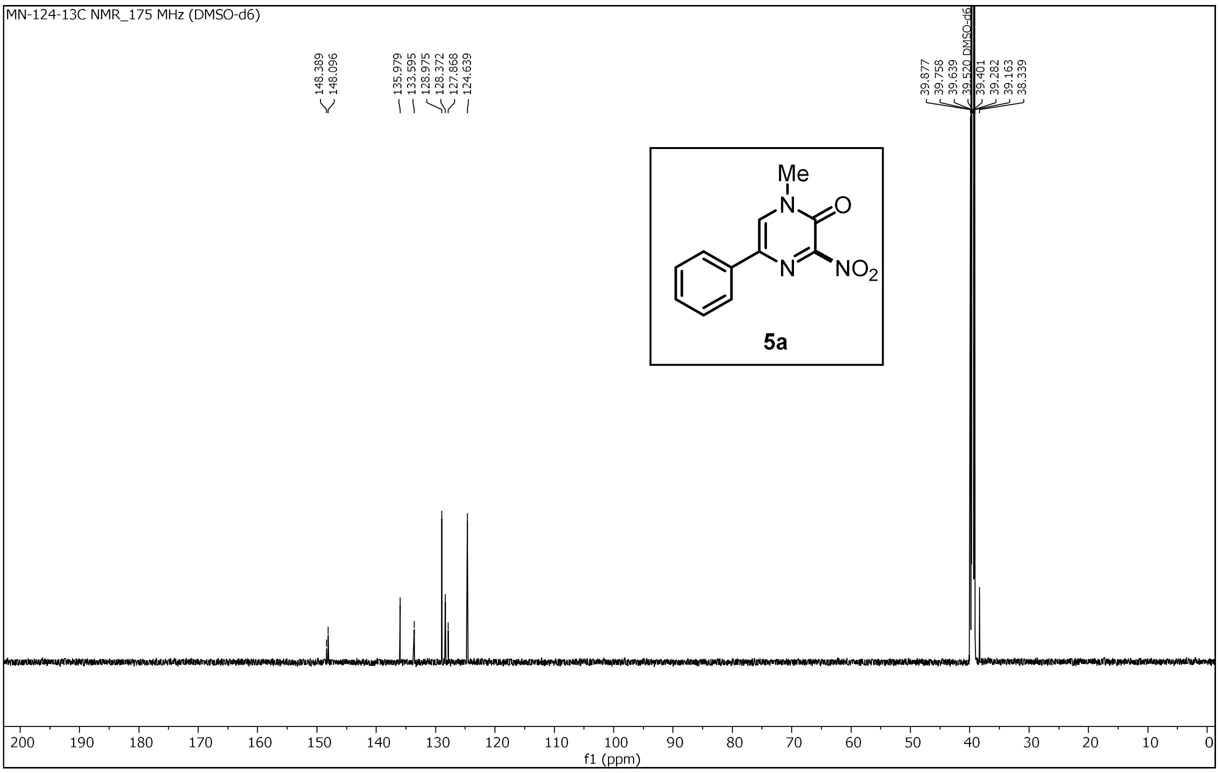
MN-124-13C NMR_175 MHz (DMSO-d6)
148.389 148.096
135.979 133.595 128.975 128.372 127.868 124.639
39.877 39.758 39.639 39.520 DMSO-d6 39.401 39.282 39.163 38.339
Me
N
O
N
NO2
5a
f1 (ppm)

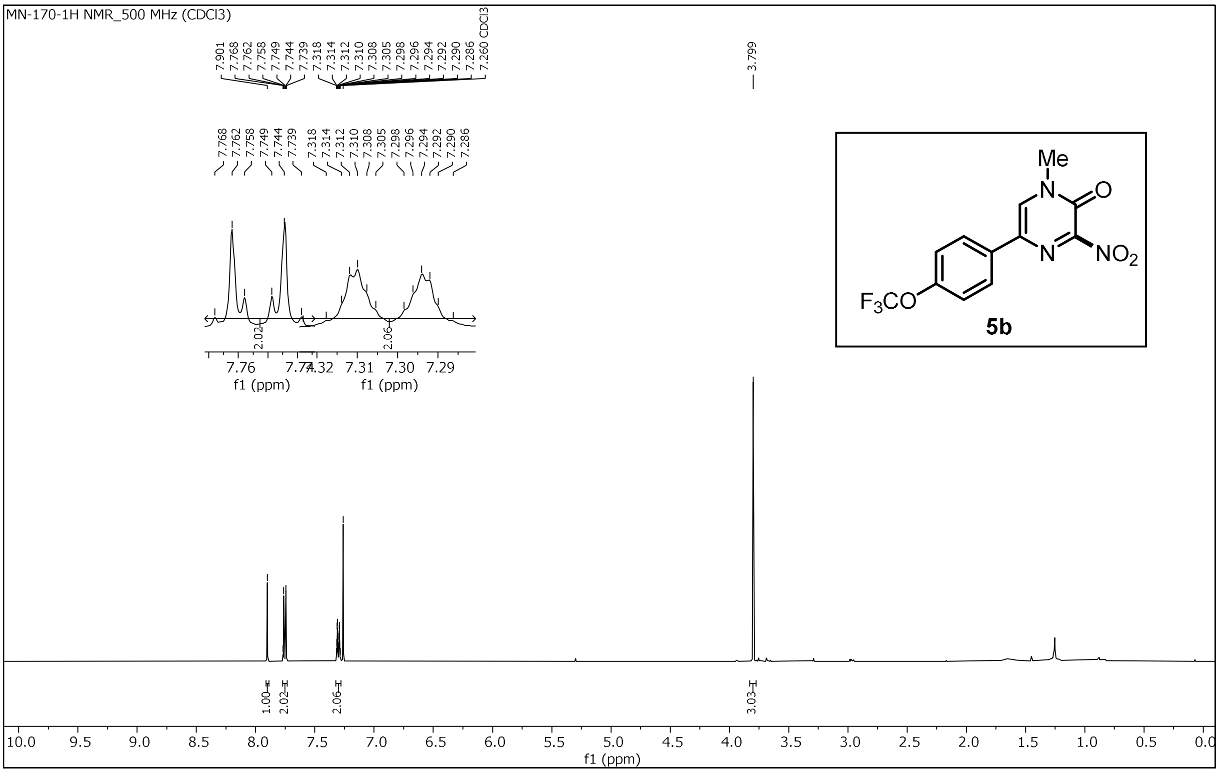
MN-170-1H NMR_500 MHz (CDCl3)
5b
f1 (ppm)

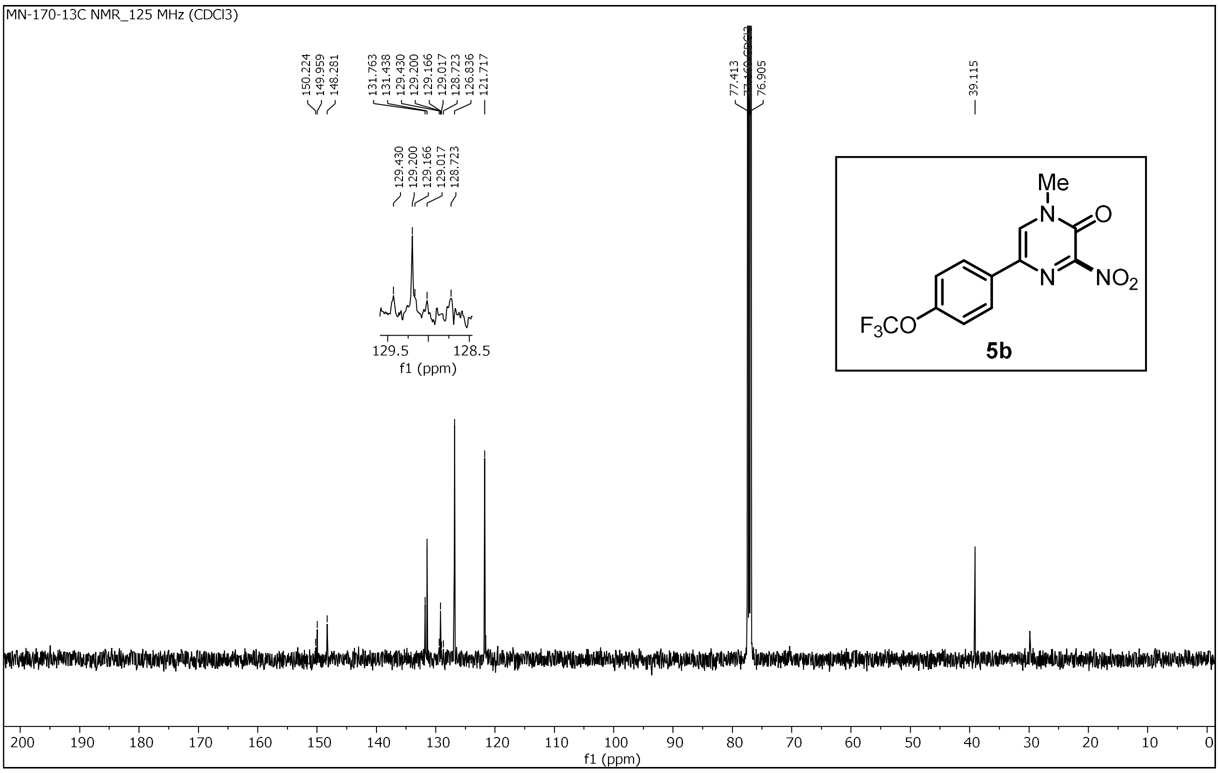
MN-170-13C NMR_125 MHz (CDCl3)
5b
f1 (ppm)

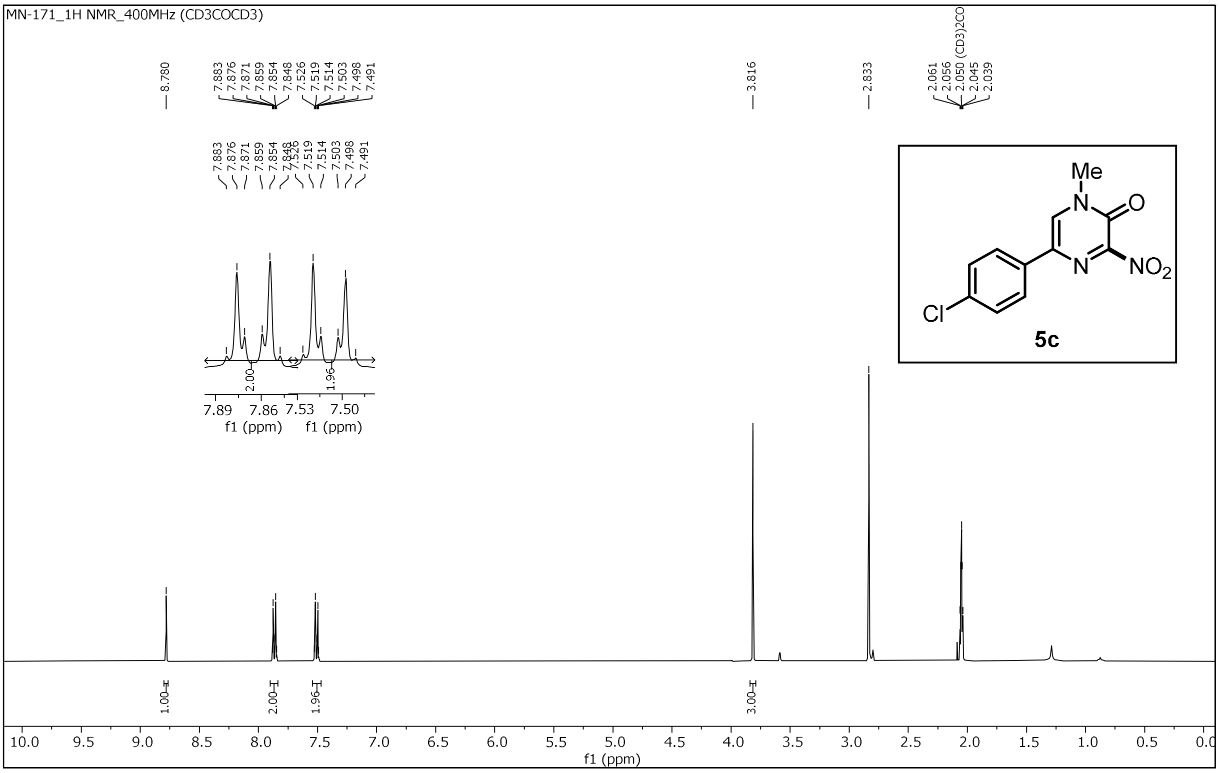
MN-171_1H NMR_400MHz (CD3COCD3)
8.780
7.883 7.876 7.871 7.859 7.854 7.848 7.526 7.519 7.514 7.503 7.498 7.491
3.816
2.833
2.061 2.056 2.050 (CD3)2CO 2.045 2.039
Me
N
O
N
NO2
Cl
5c
2.00
1.96
7.89 7.86 7.53 7.50
f1 (ppm) f1 (ppm)
1.00
2.00
1.96
3.00
10.0 9.5 9.0 8.5 8.0 7.5 7.0 6.5 6.0 5.5 5.0 4.5 4.0 3.5 3.0 2.5 2.0 1.5 1.0 0.5 0.0
f1 (ppm)

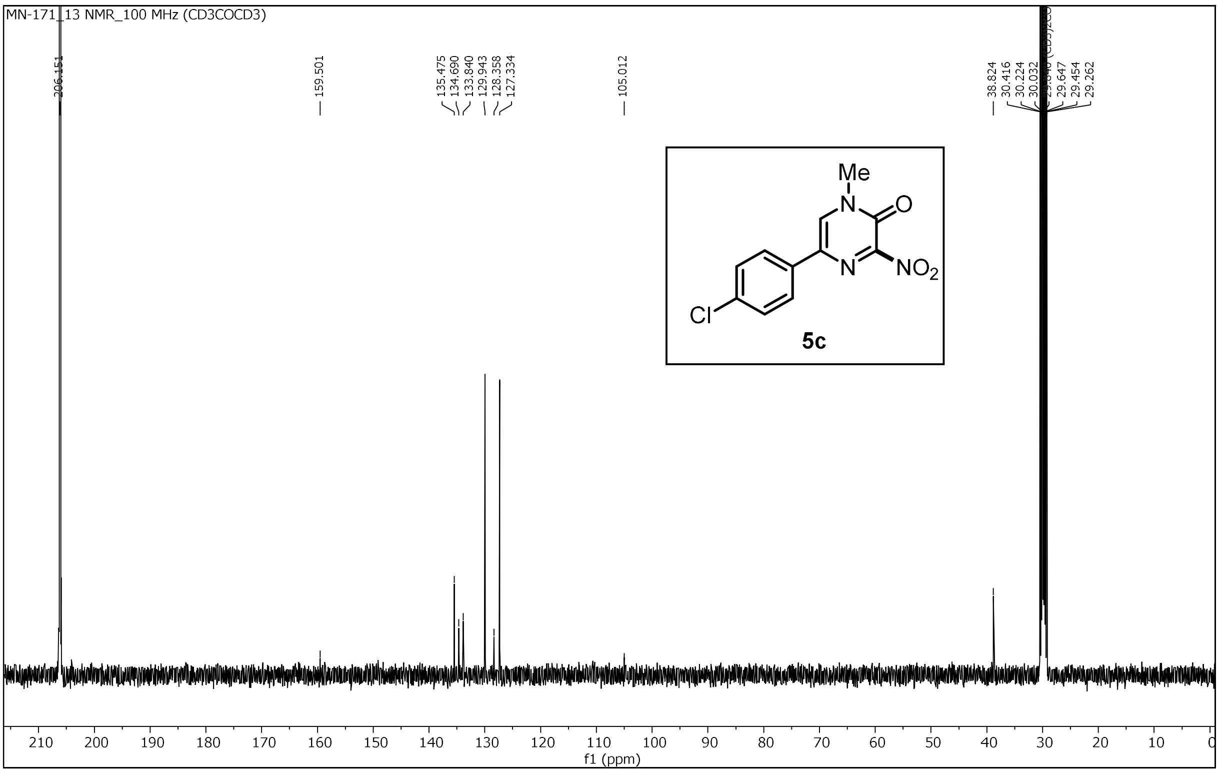
MN-171_13 NMR_100 MHz (CD3COCD3)
206.151
159.501
135.475 134.690 133.840 129.943 128.358 127.334
105.012
38.824 30.416 30.224 30.032 29.647 29.454 29.262
Me
N
O
N
NO2
Cl
5c
210 200 190 180 170 160 150 140 130 120 110 100 90 80 70 60 50 40 30 20 10 0
f1 (ppm)

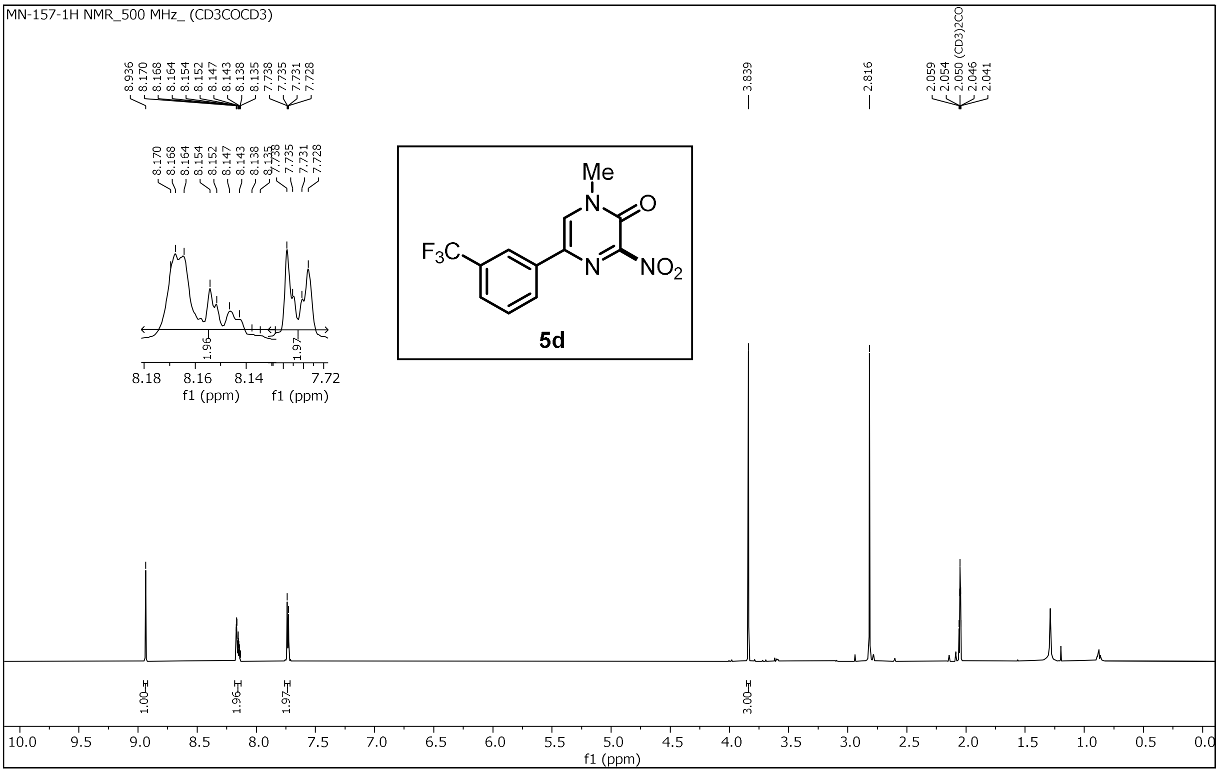
MN-157-1H NMR_500 MHz_ (CD3COCD3)
3.839
2.816
2.050 (CD3)2CO
5d
f1 (ppm)

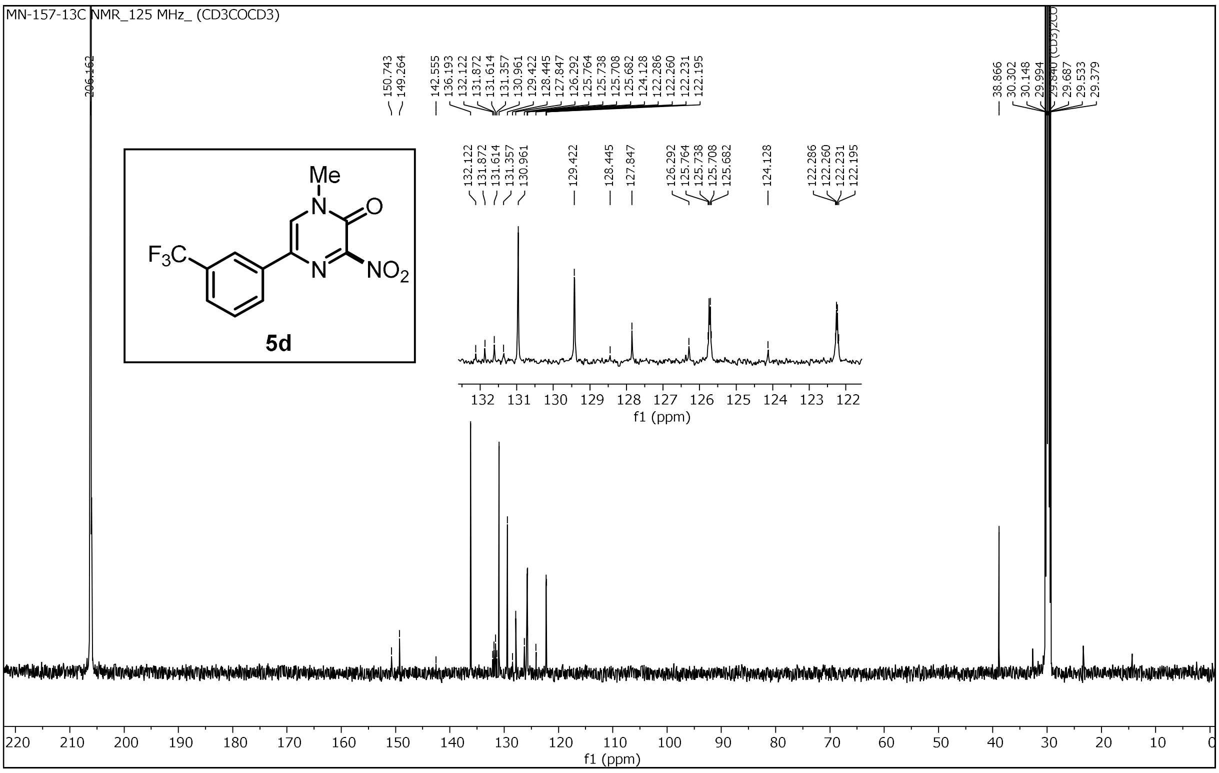
MN-157-13C NMR_125 MHz_ (CD3COCD3)
206.162
150.743
149.264
142.555
136.193
38.866
5d
f1 (ppm)

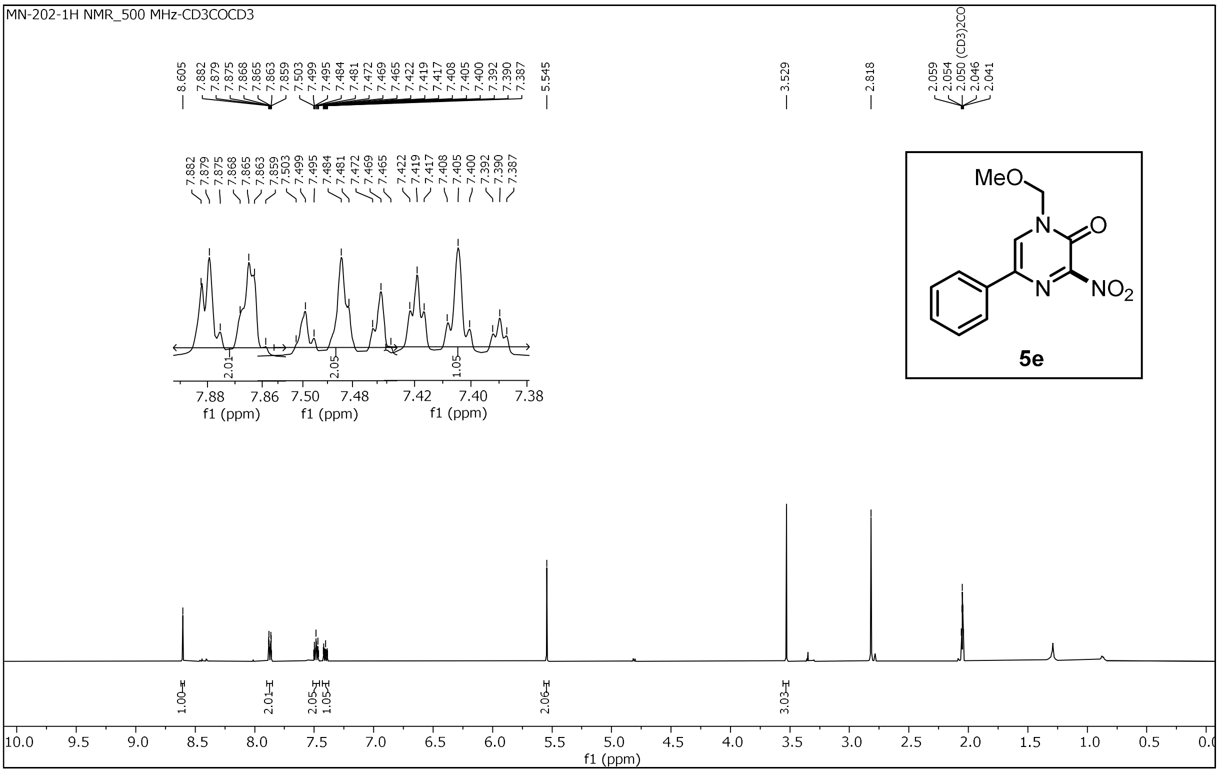
MN-202-1H NMR_500 MHz-CD3COCD3
8.605
7.882
7.879
7.875
7.868
7.865
7.863
7.859
7.503
7.499
7.495
7.484
7.481
7.472
7.469
7.465
7.422
7.419
7.417
7.408
7.405
7.400
7.392
7.390
7.387
5.545
3.529
2.818
2.059
2.054
2.050 (CD3)2CO
2.046
2.041
MeO
N
O
N
NO2
5e
2.01
2.05
1.05
7.88 7.86 7.50 7.48 7.42 7.40 7.38
f1 (ppm)
1.00
2.01
2.05
1.05
2.06
3.03
10.0 9.5 9.0 8.5 8.0 7.5 7.0 6.5 6.0 5.5 5.0 4.5 4.0 3.5 3.0 2.5 2.0 1.5 1.0 0.5
f1 (ppm)

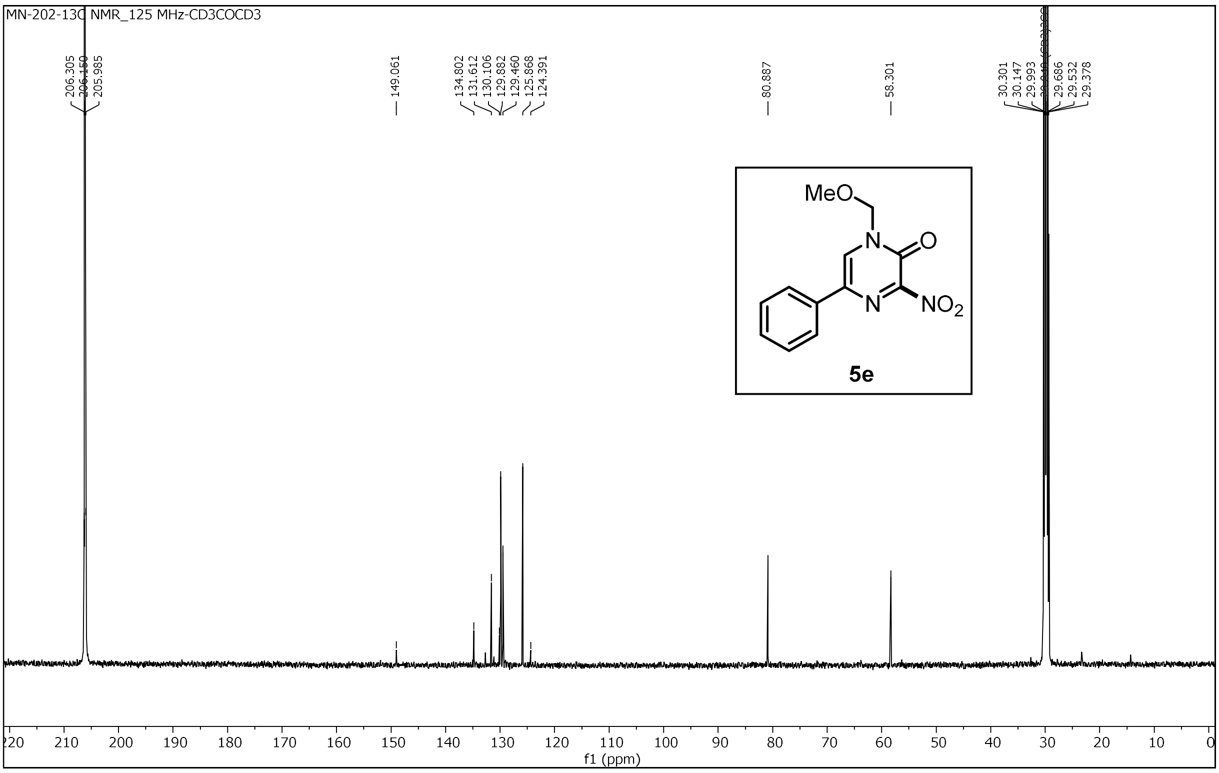
MN-202-13C NMR_125 MHz-CD3COCD3
206.305
206.150
205.985
149.061
134.802
131.612
130.106
129.882
129.460
125.868
124.391
80.887
58.301
30.301
30.147
29.993
29.686
29.532
29.378
MeO
N
O
N
NO2
5e
210 200 190 180 170 160 150 140 130 120 110 100 90 80 70 60 50 40 30 20 10
f1 (ppm)

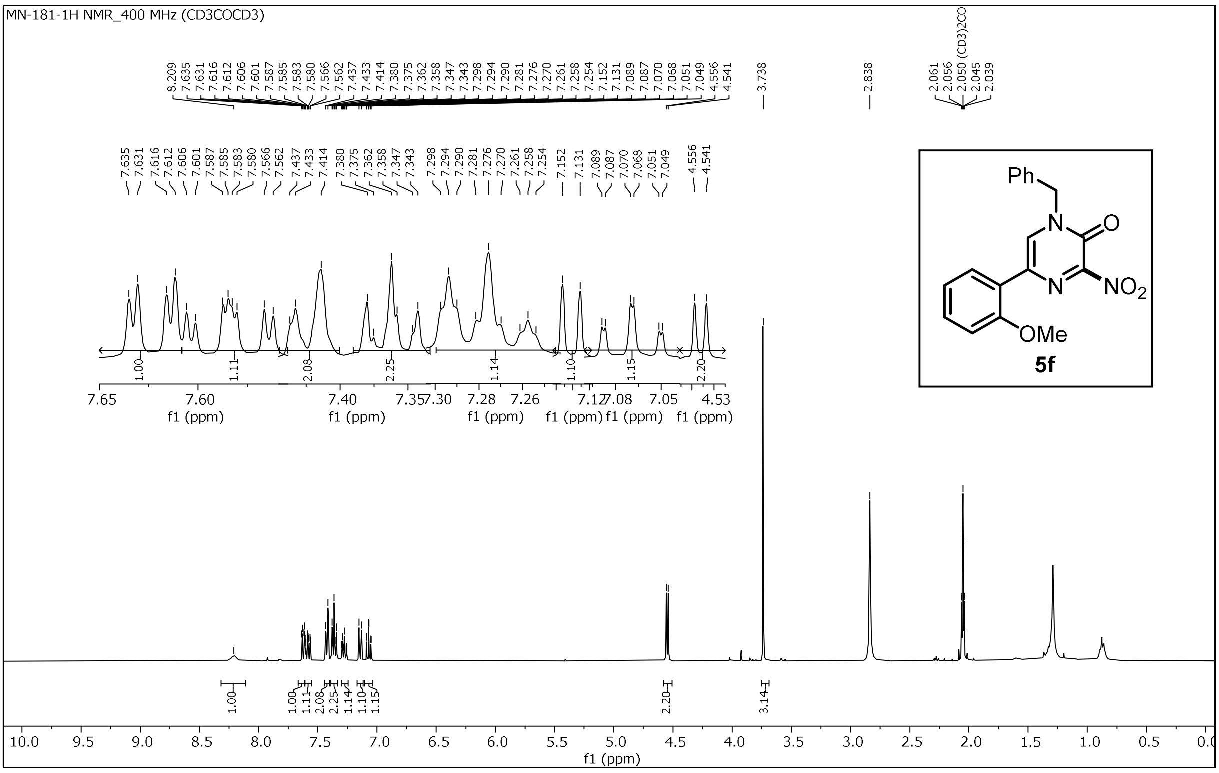
MN-181-1H NMR_400 MHz (CD3COCD3)
Ph
N
O
N
NO2
OMe
5f
f1 (ppm)

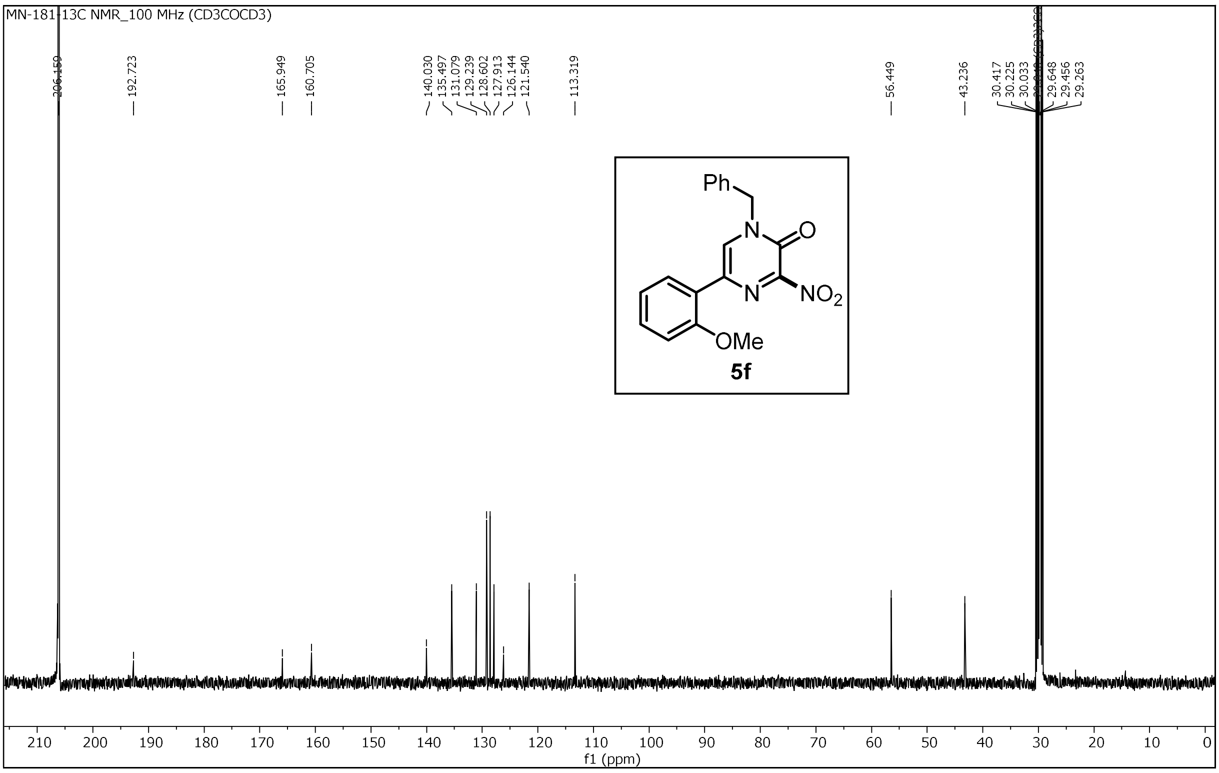
MN-181-13C NMR_100 MHz (CD3COCD3)
Ph
N
O
N
NO2
OMe
5f
f1 (ppm)

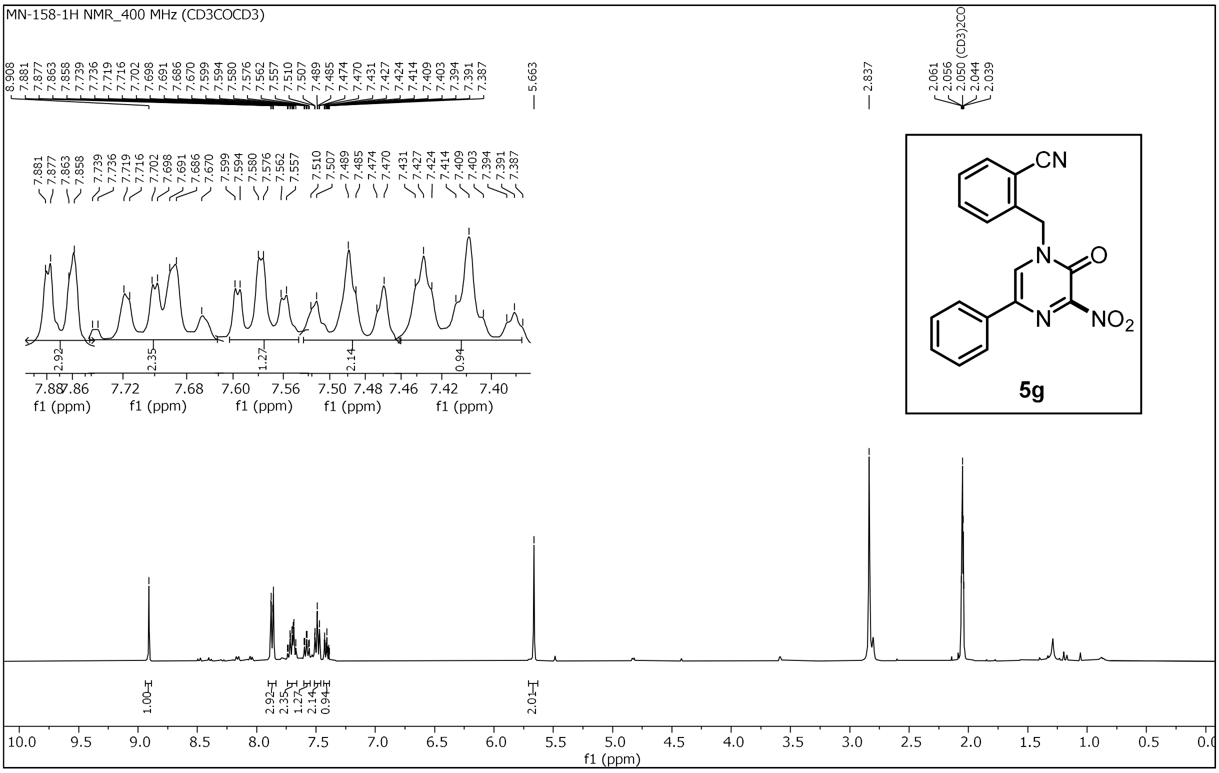
MN-158-1H NMR_400 MHz (CD3COCD3)
CN
N
O
N
NO2
5g
f1 (ppm)

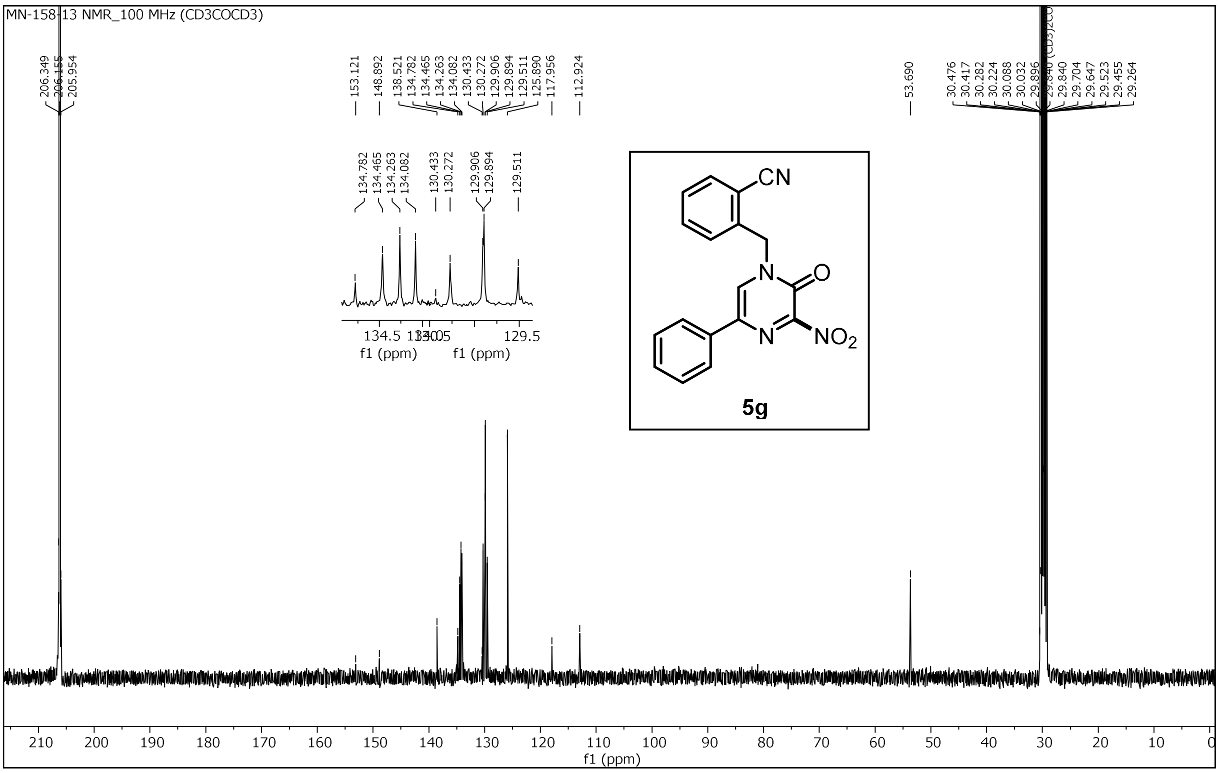
MN-158-13 NMR_100 MHz (CD3COCD3)
CN
N
O
N
NO2
5g
f1 (ppm)

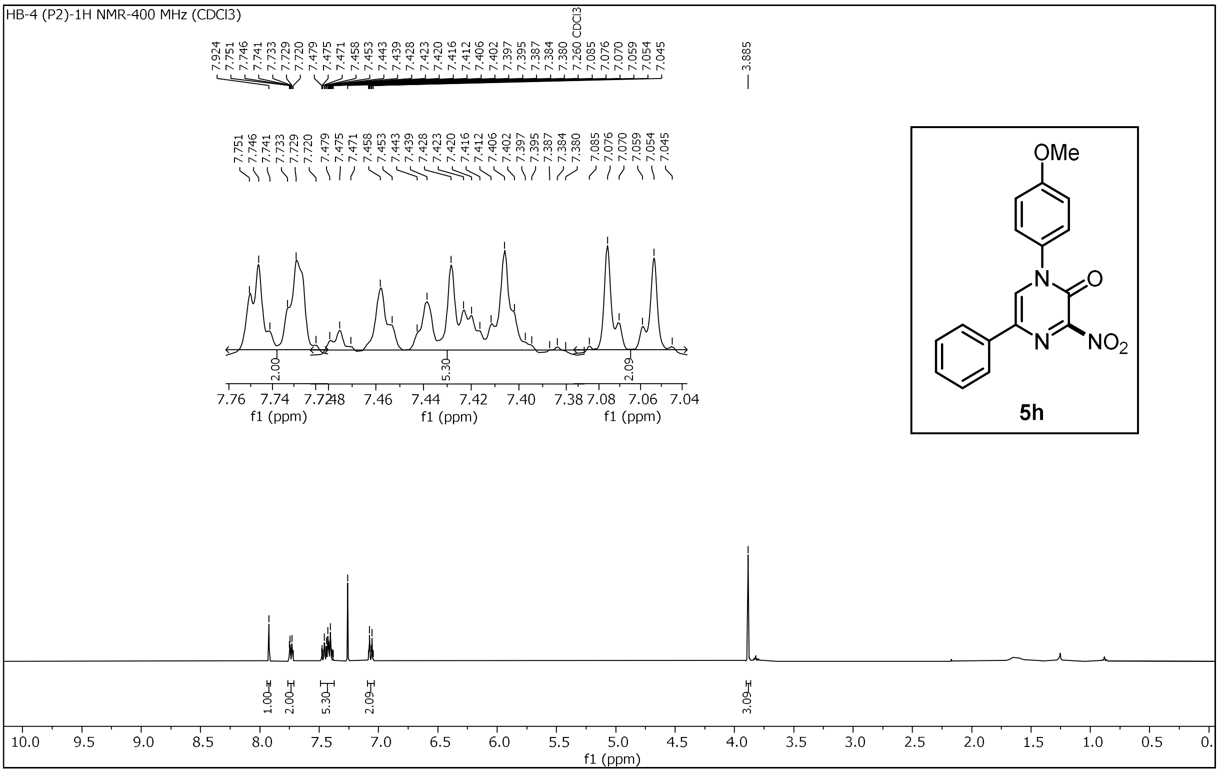

HB-4 (P2)-1H NMR-400 MHz (CDCl3)
3.885
OMe
N
O
N
NO2
5h
f1 (ppm)

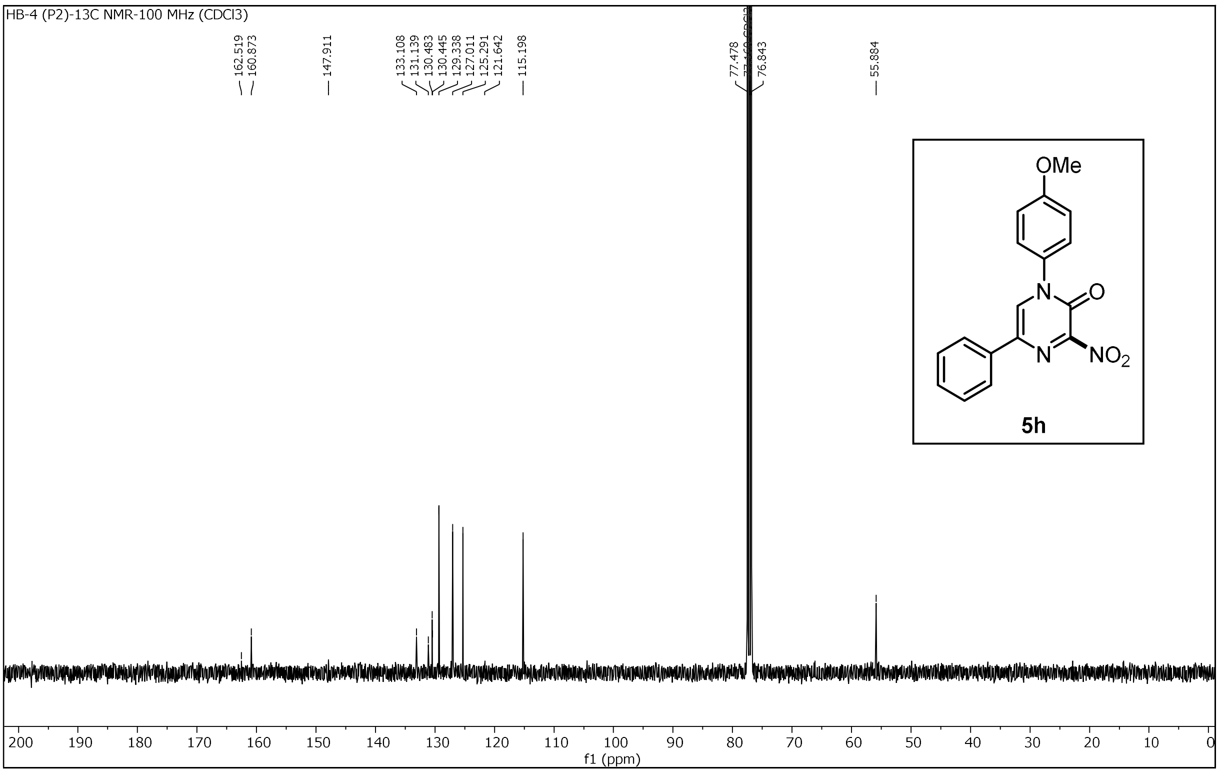

HB-4 (P2)-13C NMR-100 MHz (CDCl3)
162.519
160.873
147.911
133.108
131.139
130.483
130.445
129.338
127.011
125.291
121.642
115.198
77.478
76.843
55.884
OMe
N
O
N
NO2
5h
f1 (ppm)

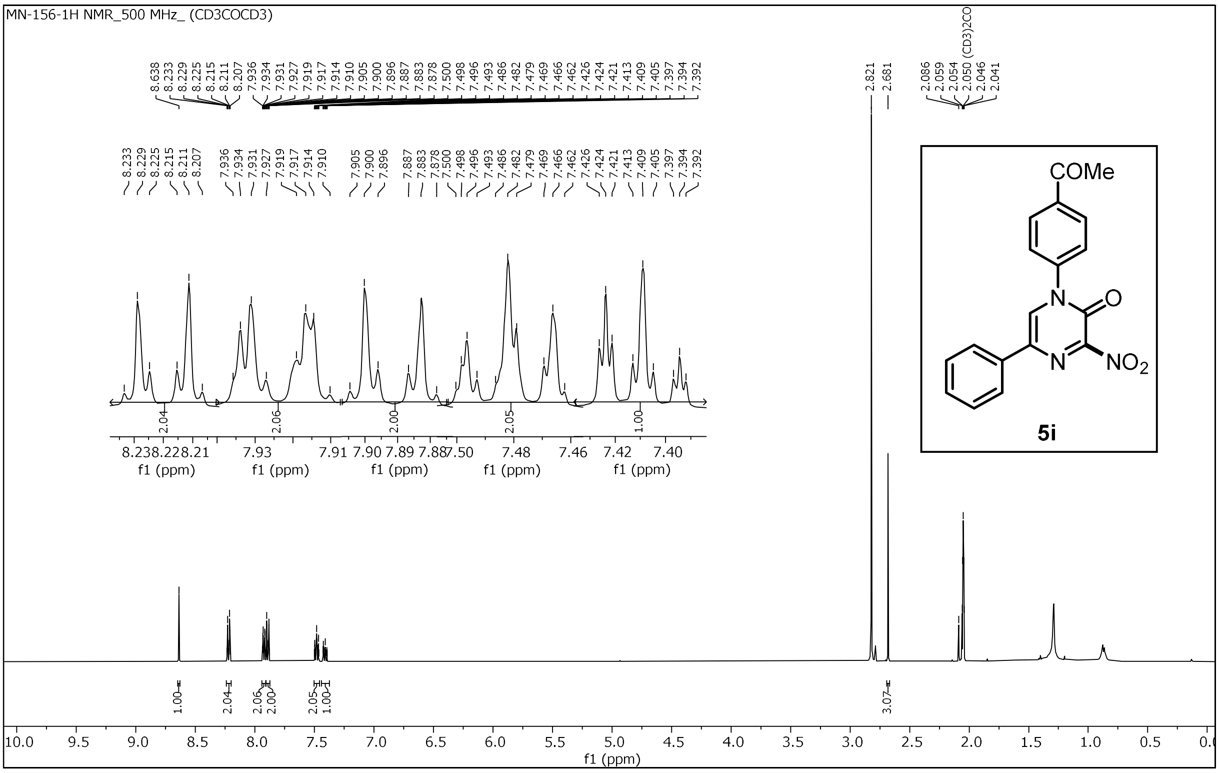
MN-156-1H NMR_500 MHz_ (CD3COCD3)
COMe
N
O
N
NO2
5i
f1 (ppm)

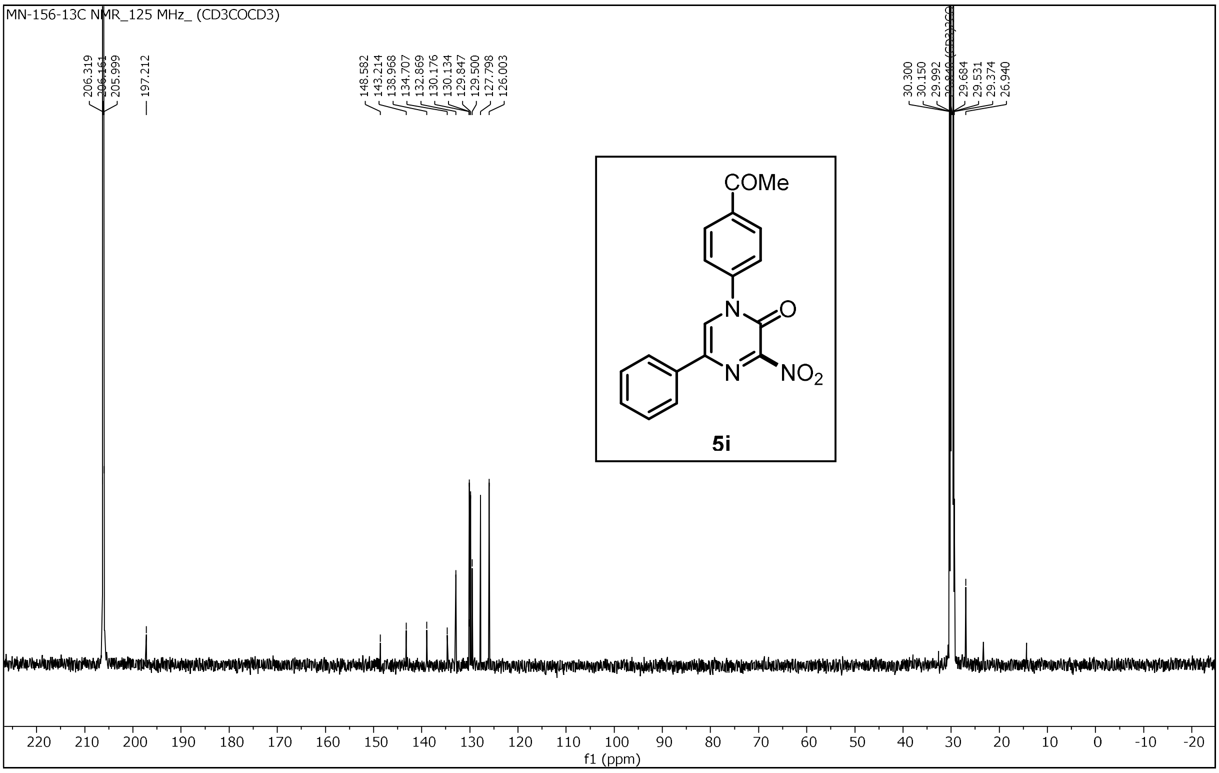
MN-156-13C NMR_125 MHz_ (CD3COCD3)
COMe
N
O
N
NO2
5i
f1 (ppm)

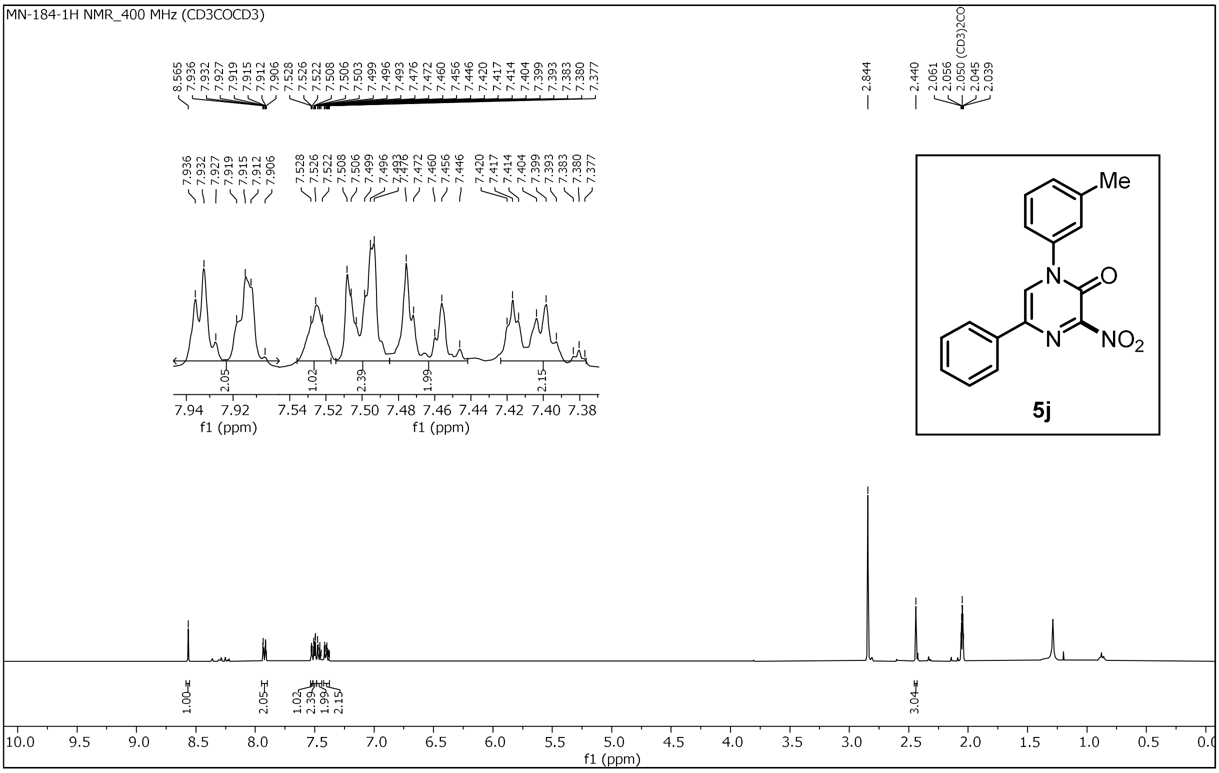
MN-184-1H NMR_400 MHz (CD3COCD3)
5j
Me
N
O
N
NO2
f1 (ppm)

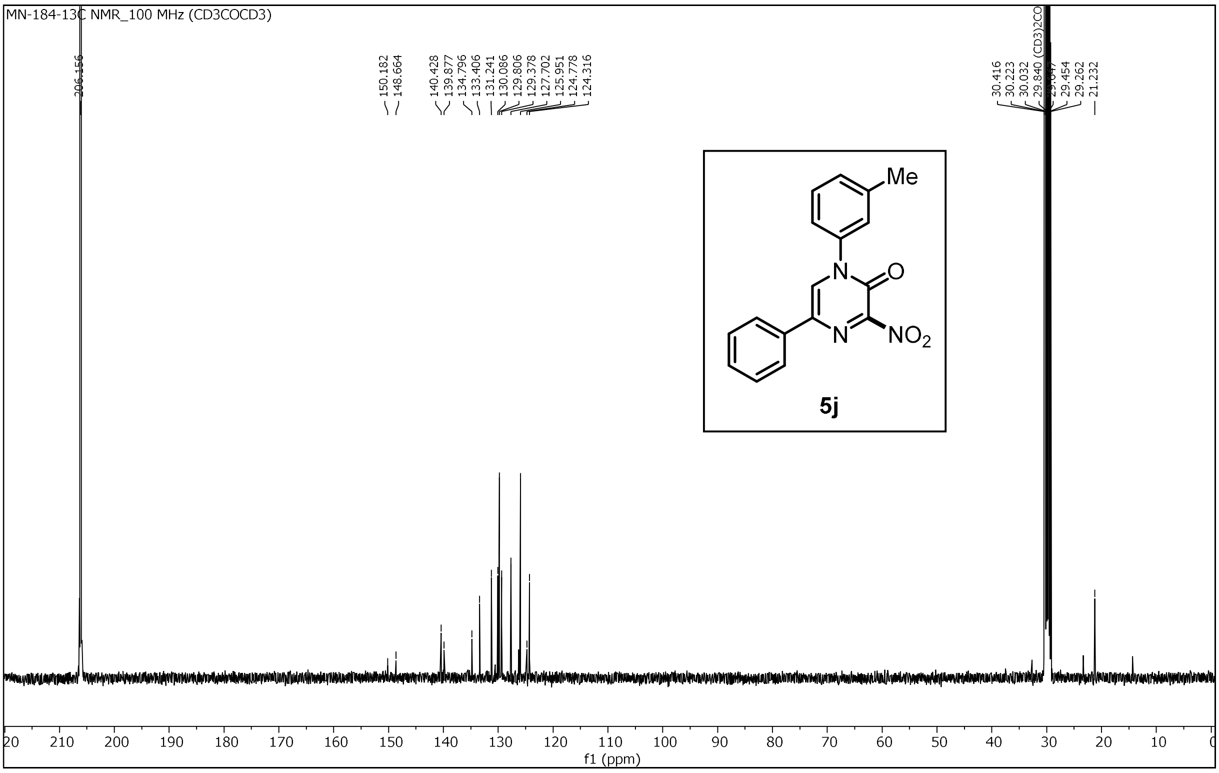
MN-184-13C NMR_100 MHz (CD3COCD3)
5j
Me
N
O
N
NO2
f1 (ppm)

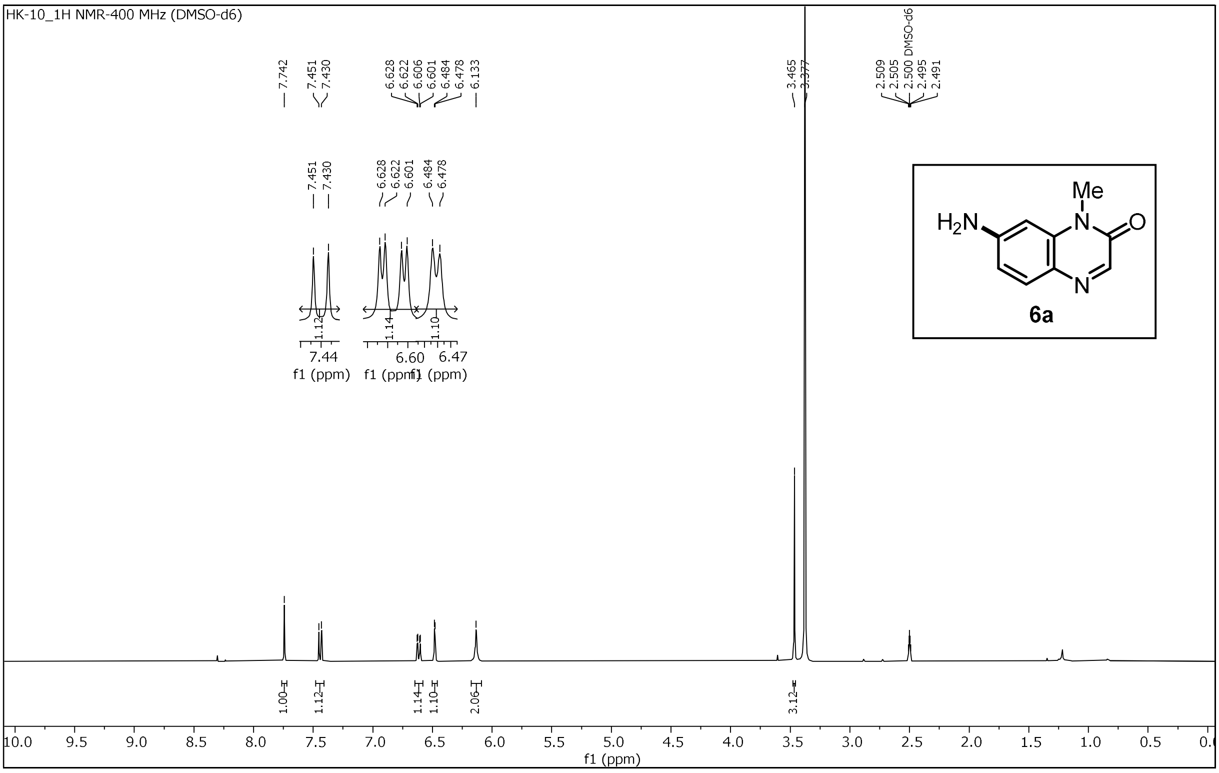
HK-10_1H NMR-400 MHz (DMSO-d6)
7.742
7.451
7.430
6.628
6.622
6.606
6.601
6.484
6.478
6.133
3.465
3.377
2.509
2.505
2.500 DMSO-d6
2.495
2.491
Me
H_2N
N
O
6a
7.44
6.60
6.47
f1 (ppm)
1.00
1.12
1.14
1.10
2.06
3.12
10.0
9.5
9.0
8.5
8.0
7.5
7.0
6.5
6.0
5.5
5.0
4.5
4.0
3.5
3.0
2.5
2.0
1.5
1.0
0.5
f1 (ppm)

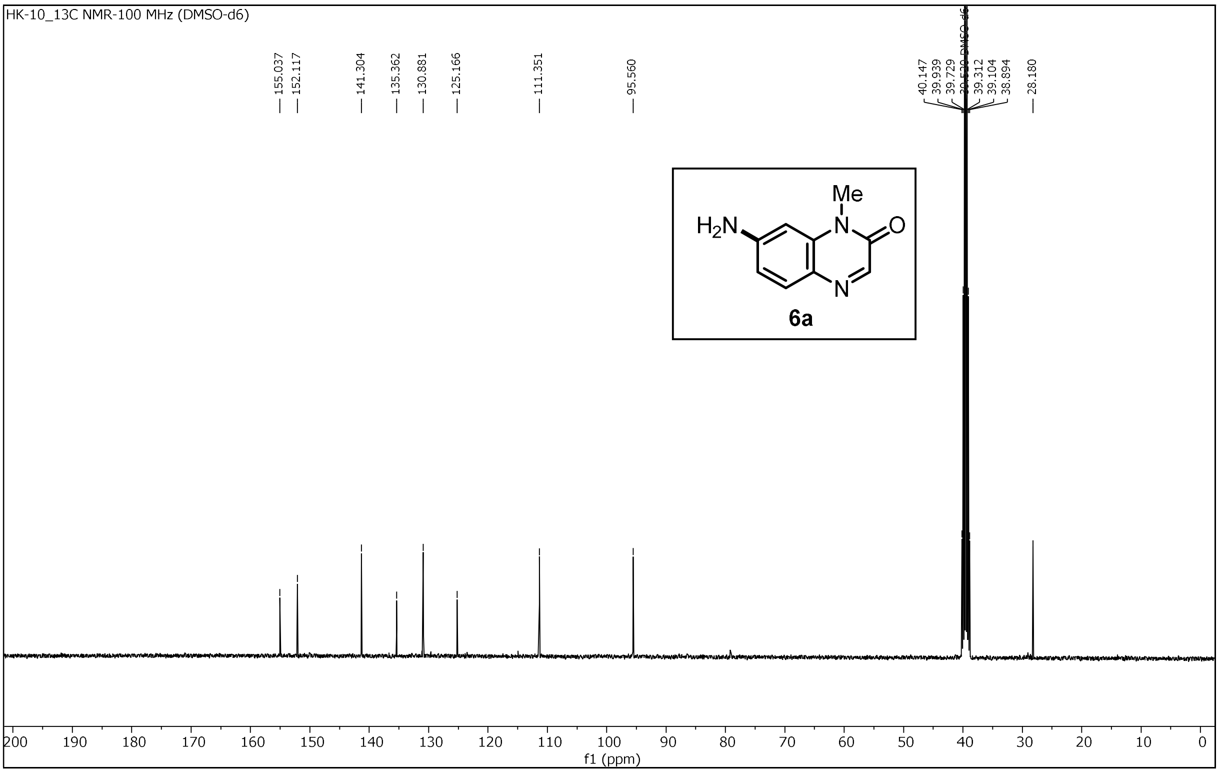
HK-10_13C NMR-100 MHz (DMSO-d6)
155.037
152.117
141.304
135.362
130.881
125.166
111.351
95.560
40.147
39.939
39.729
39.312
39.104
38.894
28.180
Me
H_2N
N
O
6a
200
190
180
170
160
150
140
130
120
110
100
90
80
70
60
50
40
30
20
10
0
f1 (ppm)

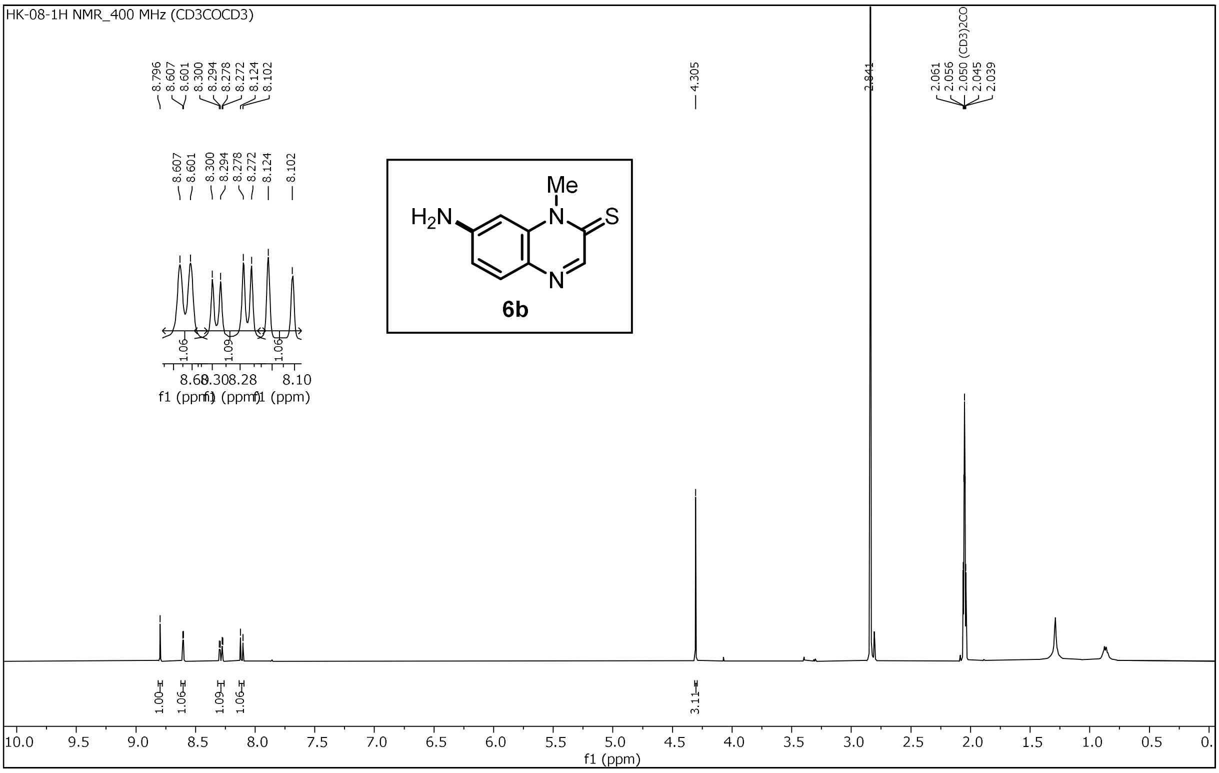
HK-08-1H NMR_400 MHz (CD3COCD3)
8.796
8.607
8.601
8.300
8.294
8.278
8.272
8.124
8.102
4.305
2.841
2.061
2.056
2.050 (CD3)2CO
2.045
2.039
H2N
Me
N
S
N
6b
1.00
1.06
1.09
1.06
3.11
f1 (ppm)

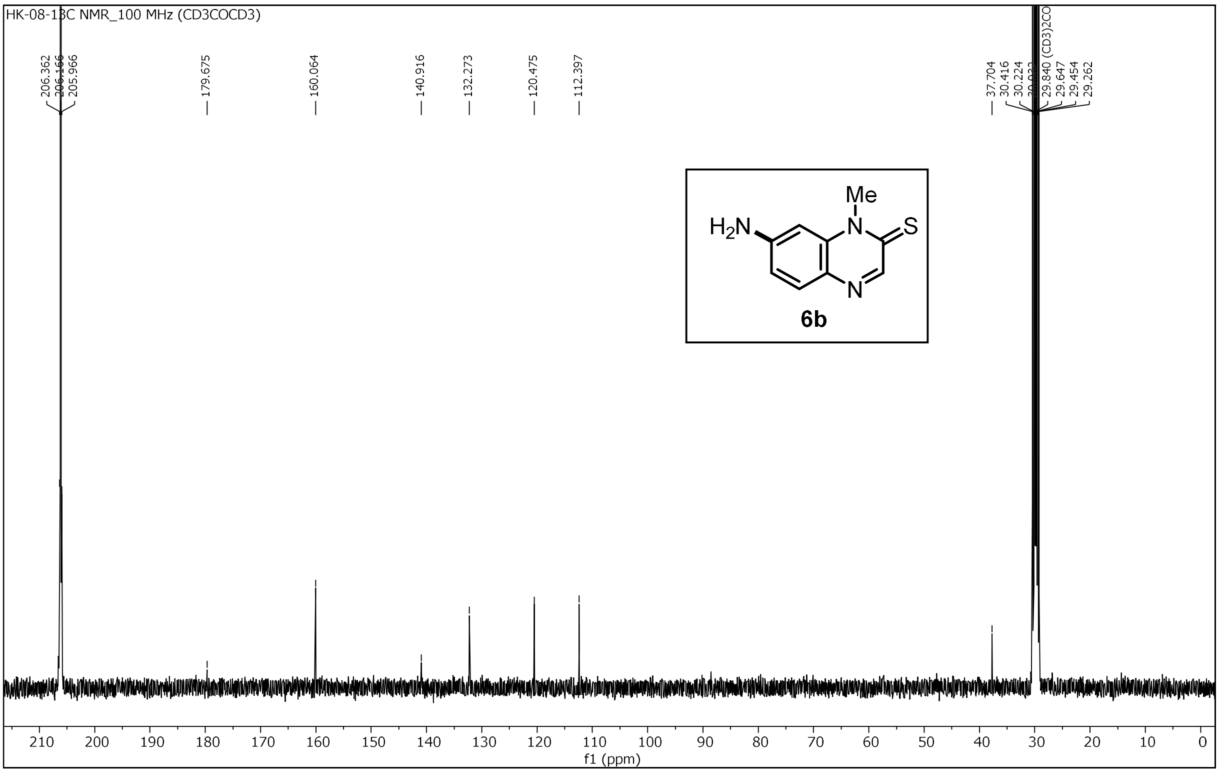
HK-08-13C NMR_100 MHz (CD3COCD3)
206.362
205.966
179.675
160.064
140.916
132.273
120.475
112.397
37.704
30.416
30.224
29.840 (CD3)2CO
29.647
29.454
29.262
H2N
Me
N
S
N
6b
f1 (ppm)

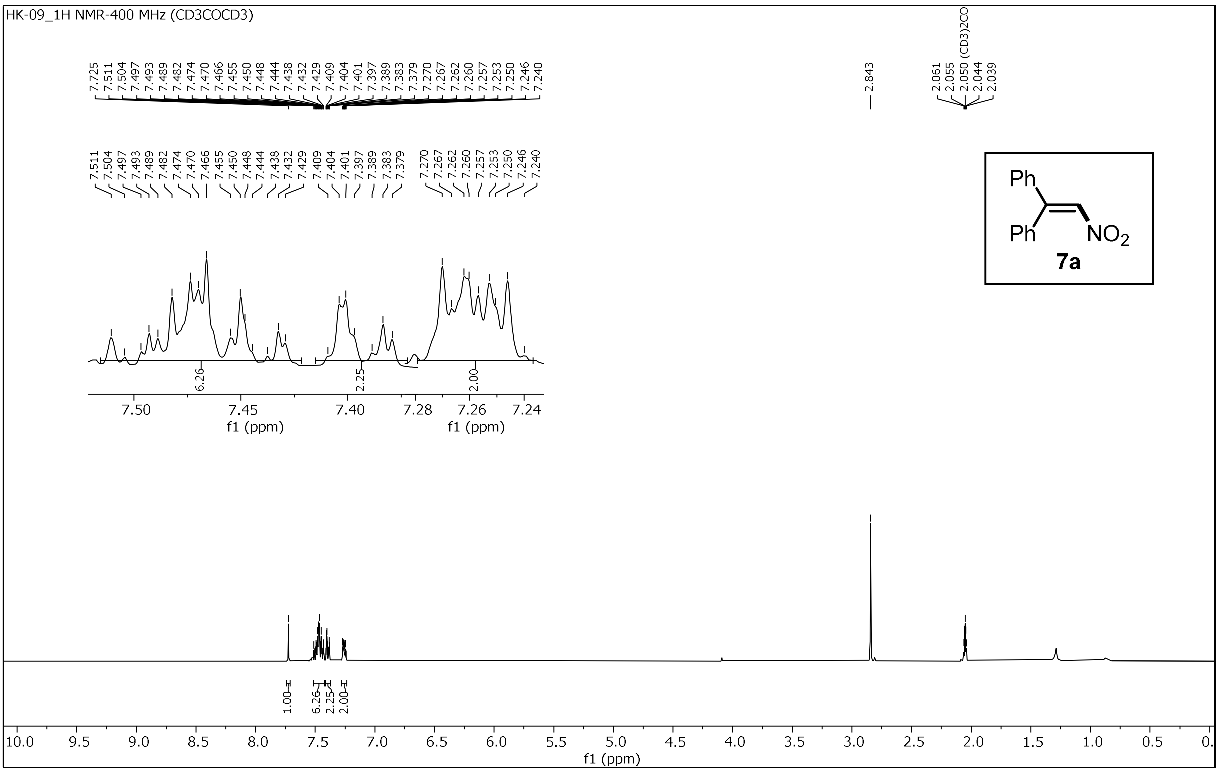
HK-09_1H NMR-400 MHz (CD3COCD3)
2.843
2.061
2.055
2.050 (CD3)2CO
2.044
2.039
Ph
Ph
NO2
7a
6.26
2.25
2.00
1.00
f1 (ppm)

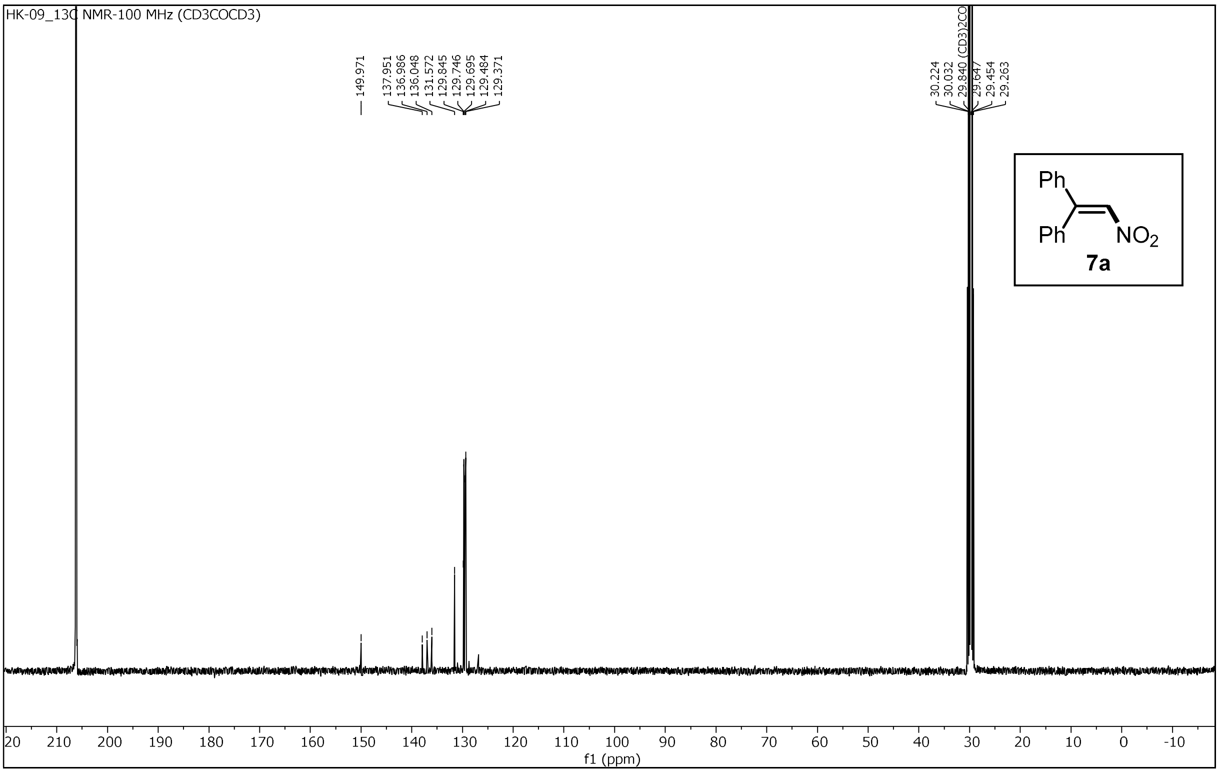
HK-09_13C NMR-100 MHz (CD3COCD3)
149.971
137.951
136.986
136.048
131.572
129.845
129.746
129.695
129.484
129.371
30.224
30.032
29.840 (CD3)2CO
29.647
29.454
29.263
Ph
Ph
NO2
7a
f1 (ppm)

^{19}F NMR spectra of F-containing compounds 5b and 5d

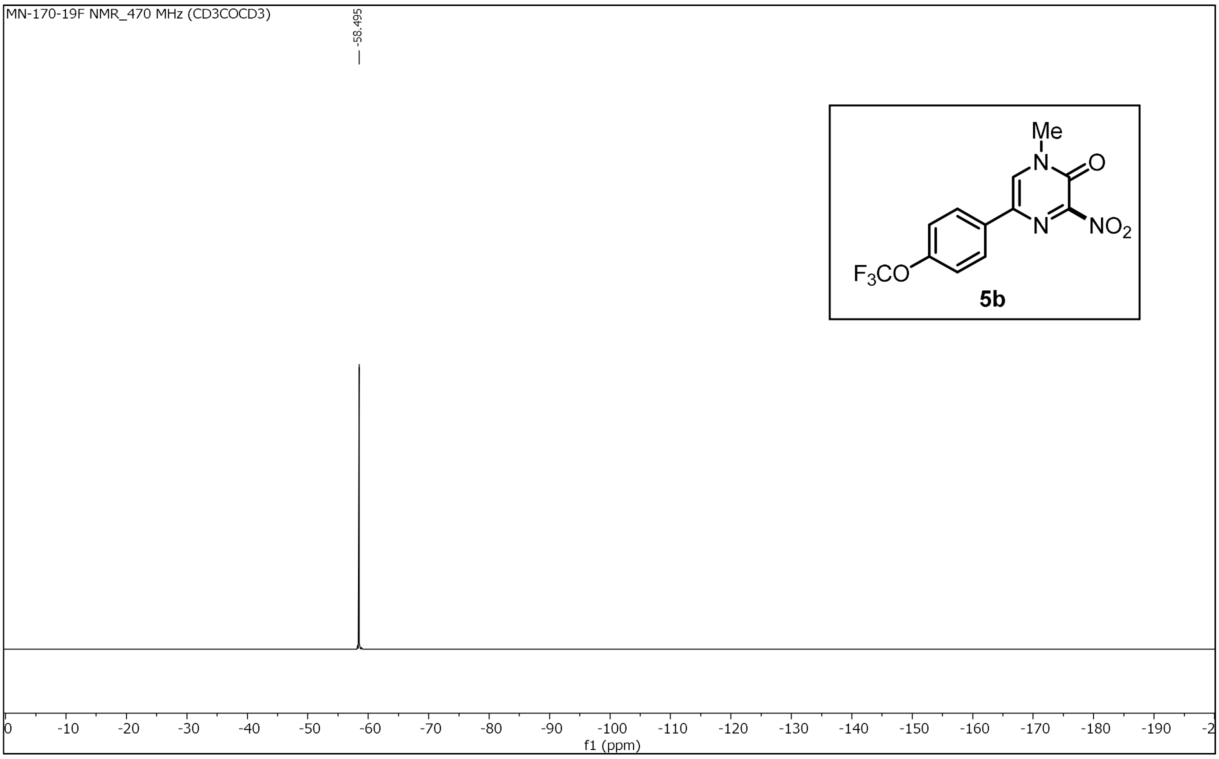

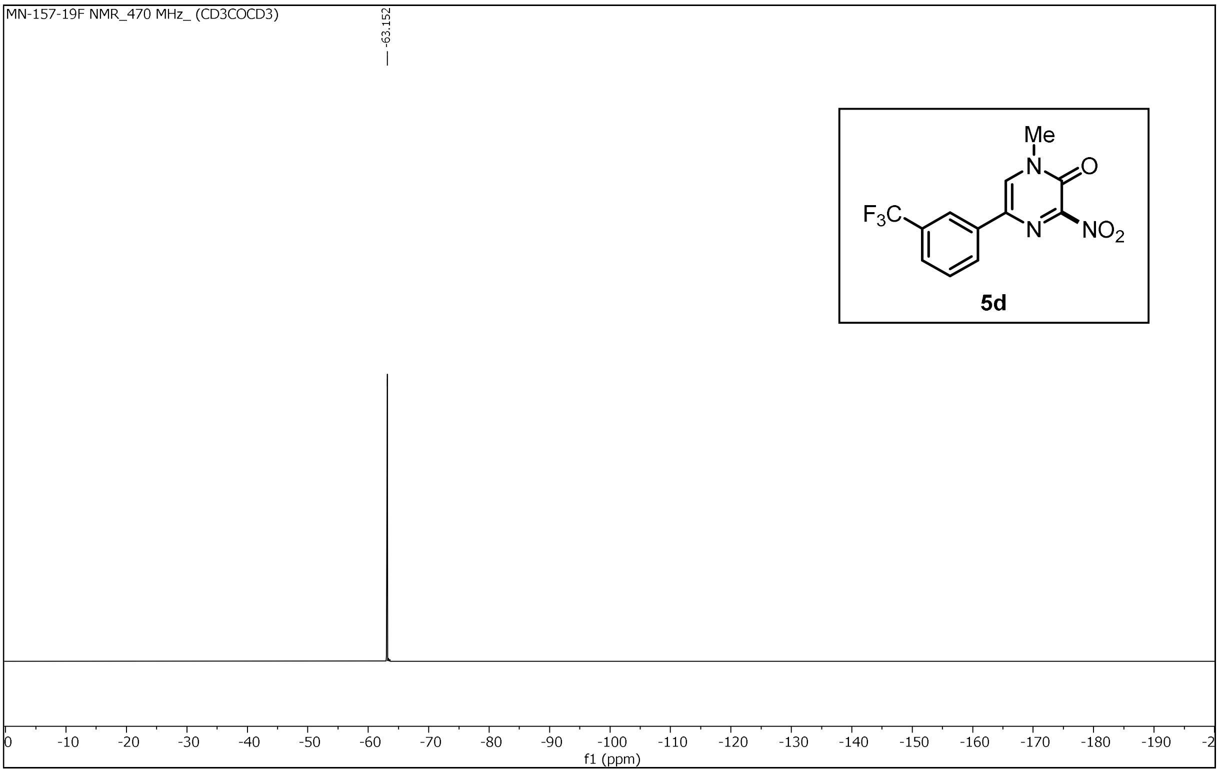

Supplement: Supplementary file 1 — Supplementary file1 (PDF 3470 kb) [file 12272_2021_1351_MOESM1_ESM.pdf]
